# A combined computational strategy of sequence and structural analysis predicts the existence of a functional eicosanoid pathway in *Drosophila melanogaster*

**DOI:** 10.1371/journal.pone.0211897

**Published:** 2019-02-12

**Authors:** Michael Scarpati, Yan Qi, Shubha Govind, Shaneen Singh

**Affiliations:** 1 Brooklyn College of the City University of New York, Brooklyn, New York, United States of America; 2 PhD program in Biology, Graduate Center of the City University of New York, New York, New York, United States of America; 3 PhD program in Biochemistry, Graduate Center of the City University of New York, New York, New York, United States of America; 4 The City College of the City University of New York, New York, New York, United States of America; Universidade Nova de Lisboa Instituto de Tecnologia Quimica e Biologica, PORTUGAL

## Abstract

This study reports on a putative eicosanoid biosynthesis pathway in *Drosophila melanogaster* and challenges the currently held view that mechanistic routes to synthesize eicosanoid or eicosanoid-like biolipids do not exist in insects, since to date, putative fly homologs of most mammalian enzymes have not been identified. Here we use systematic and comprehensive bioinformatics approaches to identify most of the mammalian eicosanoid synthesis enzymes. Sensitive sequence analysis techniques identified candidate *Drosophila* enzymes that share low global sequence identities with their human counterparts. Twenty *Drosophila* candidates were selected based upon (a) sequence identity with human enzymes of the cyclooxygenase and lipoxygenase branches, (b) similar domain architecture and structural conservation of the catalytic domain, and (c) presence of potentially equivalent functional residues. Evaluation of full-length structural models for these 20 top-scoring *Drosophila* candidates revealed a surprising degree of conservation in their overall folds and potential analogs for functional residues in all 20 enzymes. Although we were unable to identify any suitable candidate for lipoxygenase enzymes, we report structural homology models of three fly cyclooxygenases. Our findings predict that the *D*. *melanogaster* genome likely codes for one or more pathways for eicosanoid or eicosanoid-like biolipid synthesis. Our study suggests that classical and/or novel eicosanoids mediators must regulate biological functions in insects–predictions that can be tested with the power of *Drosophila* genetics. Such experimental analysis of eicosanoid biology in a simple model organism will have high relevance to human development and health.

## Introduction

The eicosanoids are a family of biologically active lipids that have been implicated in various signaling pathways, with a central role in mammalian immunity and inflammation [1–3]. The canonical eicosanoid biosynthesis pathway begins with the release of fatty acids, primarily arachidonic acid (AA), from membrane phospholipids when phospholipase A_2_ is activated [4,5]. The canonical pathway then diverges, depending on whether the fatty acid substrate is processed by a cyclooxygenase (COX), lipoxygenase (LOX), or a P450 epoxygenase (P450E). The COX enzymes generate prostanoids (prostaglandins, prostacyclins, and thromboxanes), resolvins, and electrophilic oxo-derivatives (EFOX) of docosahexaenoic acid (DHA) [6–8] whereas the LOX enzymes produce the leukotrienes, hydroperoxyeicosatetraenoic acids (HPETEs), hydroxyeicosatetraenoic acids (HETEs), and lipoxins [9]. There is some cross-talk between the COX and LOX pathways, as both are known to produce hydroxyoctadecadienoic acids (HODEs) [10,11]. The P450 epoxygenase pathway yields epoxyeicosatrienoic acids (EETs) [12]. These downstream products regulate diverse signaling pathways and biological processes [2,3,12–14]

The COX and LOX branches of the eicosanoid pathway are well-characterized in humans. The COX enzymes (prostaglandin G/H synthases) are encoded by *COX-1* (*PTGS1*) and *COX-2* (*PTGS2*) [6]. *COX-1* is constitutively expressed at low levels by most cells. In contrast, *COX-2* is upregulated at sites of inflammation and during tumor progression [6,15]. COX-1 and COX-2 are membrane-associated heme-containing homodimers that catalyze the conversion of an 18–22 carbon polyunsaturated fatty acid (PUFA, typically AA, an ω-6, 20-carbon fatty acid) into prostaglandins G_2_ and H_2_[16]. Although AA is the preferred substrate, the COX enzymes are known to oxygenate eicosapentaenoic acid (EPA, 20:5 n-3), linoleic acid (LA, 18:2 n-6), dihomo-gamma-linolenic acid (DHLA), and docosahexaenoic acid (DHA), with varying efficiencies [16–18]. This initial substrate choice is significant, as it determines whether the downstream prostanoid will tend to be pro- or anti-inflammatory. Fatty acids with two double bonds (e.g., AA) are converted into series-2 prostanoids (*e*.*g*., PGH_2_, PGE_2_), which are associated with pro-inflammatory signaling. In contrast, fatty acid substrates with three or four double bonds (e.g., EPA, DHLA, respectively) yield series-1 and 3 prostanoids (PGH_1_, PGE_3_), which are associated with anti-inflammatory signaling. Some COX products, such as the anti-inflammatory resolvins are synthesized only from EPA and DHA, whereas the pro- and anti-inflammatory 9- and 13-HODEs are generated from LA by COX enzymes [8,10]. Nevertheless, AA is the primary substrate for COX enzymes and so COX signaling tends to be pro-inflammatory. As a result, non-steroidal anti-inflammatory drugs (NSAIDs) which inhibit COX-1/2’s ability to generate pro-inflammatory products, are particularly important. PGH2 is subsequently converted to a variety of downstream eicosanoids, including PGD_2_, PGE_2_, PGF_1α_ PGF_2α_, PGI_2_ (prostacyclin), and the thromboxanes TXA_2_ and TXB_2_. The downstream processing route depends on the availability of enzymes in a given cell type. For example, endothelial cells primarily produce PGI_2_, whereas platelets mainly produce TXA_2_ [19].

The LOX pathway operates independently from the COX pathway, diverging at the initial fatty acid processing stage. In humans, LOX enzymes are encoded by several genes (*ALOX5*, *ALOX12*, *ALOX12B*, *ALOX15*). Mammalian LOX enzymes catalyze the stereospecific dehydrogenation and dioxygenation of a PUFA, such as AA or LA, leading to the formation of a hydroxyperoxide containing a 1-hydroxyperoxy-2-*trans*-4-*cis*-pentadiene fragment [13,20]. The mammalian LOXs have been named according to the position of the carbon at which the oxygen molecule is inserted into AA, the primary fatty acid substrate, *e*.*g*., as 5-, 12-, or 15-LOX) [21]. The primary products are 5S-, 12S-, or 15S-HPETE, which can be further reduced by glutathione peroxidase to hydroxy forms (5-, 12-, 15-HETE), respectively [20,21]. 5-LOX is the only LOX that produces leukotrienes, and it is notable that catalytic activity requires the presence of a second protein (ALOX5AP), though the precise function of Five-Lipoxygenase Activating Protein (FLAP) is not known [20,22]. Following conversion of a fatty acid into 5-HPETE, downstream processing results in the terminal leukotrienes LTB_4_, LTC_4_, LTD_4_, and LTE_4_. The leukotrienes are clinically significant, as they are implicated in the allergic response and maintenance of chronic inflammation. 15-LOX (and to a lesser extent 5-LOX) produce the lipoxins LXA and LXB [23].

CYP450Es convert fatty acid substrates (*e*.*g*., AA) into four EET isomers, 5,6-, 8,9-, 11,12-, and 14,15-EETs, which function as autocrine and paracrine mediators. CYP450Es also convert EPA into epoxy-derivatives, and endocannabinoids containing 11,12- and 14,15-EETs [12]. The CYP450E protein is not examined in this study.

The COX family enzymes are highly conserved across the animal kingdom, with orthologs found in the primitive marine corals as well as the higher vertebrates [24]. Traditional genomic analyses have failed to identify COX orthologs in the known genomes of insects, unicellular organisms, or plants, although prostaglandins, their primary products, have been found in some of these organisms [25–28]. Recent studies have suggested that an insect cyclooxygenase may exist in orthologs of the *Drosophila* gene *Pxt* [27,29,30]. LOX enzymes display an even broader degree of conservation, with orthologs found across the animal kingdom and in a variety of plants [9]. Sequence and biochemical analyses have failed to identify any insect LOX orthologs, with the exception of LOX activity in the primitive insect, *Thermobia domestica* [31] and one report in *D*. *melanogaster* [28]. Thus, there is conflicting information regarding the existence of functional COX or LOX pathway in insects. Given the complexity and biomedical importance of eicosanoid biology, defining the COX and LOX pathways in a model organism is a research priority. Identification of a functionally conserved COX or LOX pathway in *D*. *melanogaster* opens up exciting research avenues for understanding the genetic basis of eicosanoid-mediated inflammation in this well-established and simple model organism.

To this end, we conducted a thorough computational analysis of the genome of *D*. *melanogaster*. While an initial BLAST search revealed candidates for the most conserved enzymes, it failed to identify likely orthologs for some of these enzymes. A more rigorous approach using iterative sequence searches combined with structural modeling revealed a surprising degree of similarity between mammalian and fly enzymes and the apparent conservation of functionally important residues even in these most distant candidate enzymes. Although we could not identify any suitable candidates for lipoxygenase enzymes, other component enzymes of the LOX branch of the pathway were identified. This suggests the possibility that eicosanoid pathway may adopt a non-canonical form in *D*. *melanogaster* and insects in general. Our study challenges the current view that insects lack a functional eicosanoid pathway and opens up tremendous possibilities of utilizing a well-established model organism for eicosanoid research.

## Materials and methods

**Fig 1:**
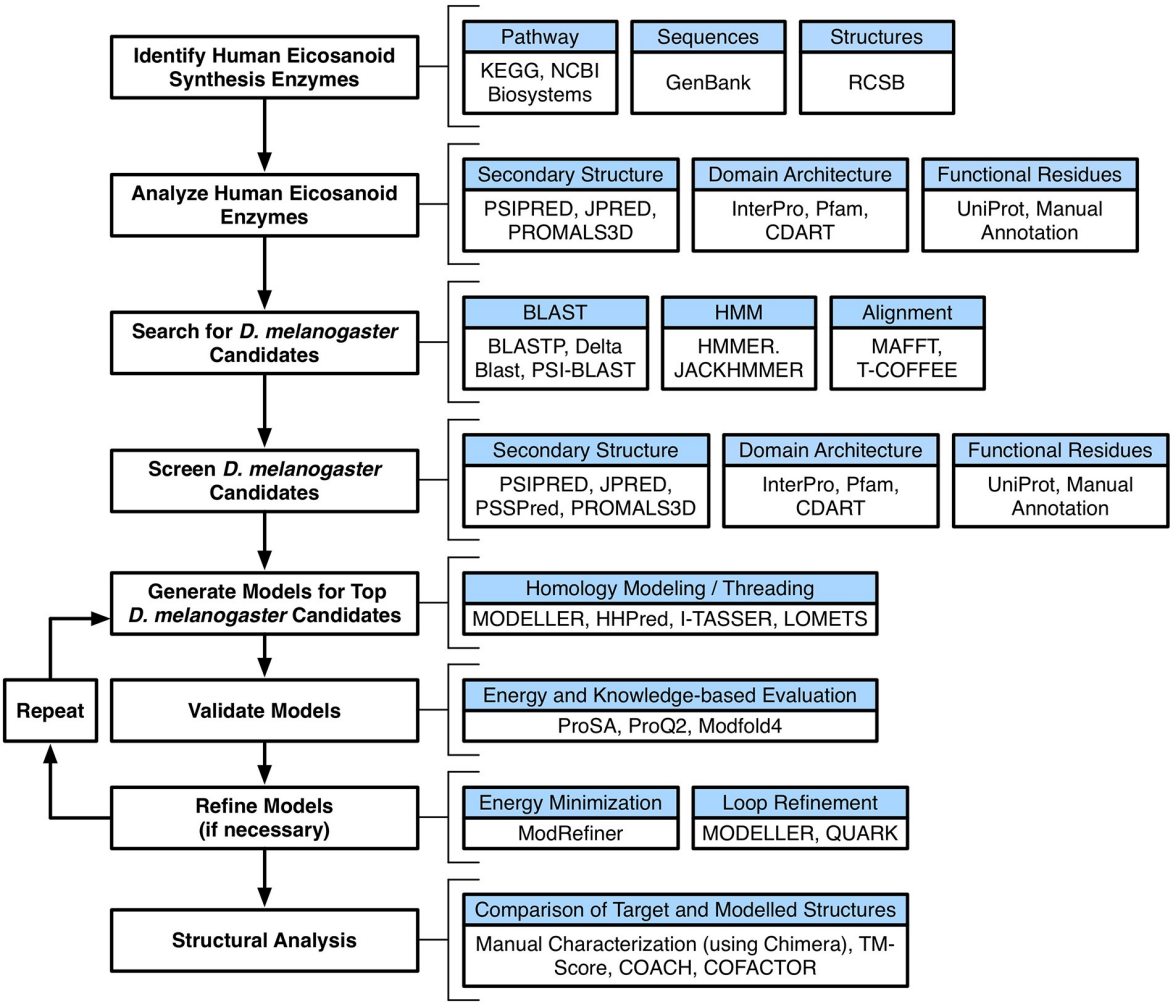
Summary of the workflow and tools used to carry out the present study. A schematic of the workflow devised to identify, model, and characterize various orthologs of the human eicosanoid pathway.

A flowchart summarizing the general protocol of the present study with the various tools used to identify and characterize putative *D*. *melanogaster* orthologs for the classic eicosanoid synthesis enzymes discussed in detail below is shown in Fig 1.

### Identification and characterization of human eicosanoid synthesis enzymes

A canonical eicosanoid synthesis pathway in humans was compiled from the NCBI BioSystems Database using the Eicosanoid Synthesis (BSID: 198888) and Arachidonic Acid pathway maps (BSID: 829971) [32]. Protein sequence(s) for each human canonical pathway gene were obtained from the NCBI RefSeq database [33]. Enzymes selected for this study are listed in Table 1 (see Results section). For each enzyme, either the sole protein product or the major isoform was selected as the representative sequence. Known and high-confidence predicted orthologs for each enzyme were identified using the Kyoto encyclopedia of genes and genomes (KEGG) Orthology database [34]. A multiple sequence alignment (MSA) for each target enzyme with the selected orthologs was then generated using PROMALS3D, followed by manual editing [35]. Domains and active sites on each identified enzyme were annotated using the Pfam, InterPro, CDART, and Uniprot databases [36–38]. Consensus secondary structure profiles were generated using the PsiPred, JPred and PSSPred servers [39–41] to confirm identified functional domains and to ascertain domain boundaries.

**Table 1:** Summary of *D*. *melanogaster* eicosanoid synthesis enzymes candidates.

Human Gene	*D*. *melanogaster* Candidate(s)	Annotation Identifier	Flybase Identifier	Percentage Identity	Percentage Similarity	E-value	Query Coverage	Template(Software) used for modeling the candidate enzyme
PTGS1, PTGS2	CG4009, isoform B	***CG4009***	FBgn0038469	25%	41%	1e-10	49%	Multiple: 4HHR, 3FAQ, 3Q9K, 1CXP, 2GJ1, and 1CVU (I-TASSER)
	Peroxinectin-like (Pxt)	***CG7660***	FBgn0261987	22%	39%	2e-09	59%	
	Cardinal	***CG6969***	FBgn0263986	20%	36%	1e-05	61%	
PTGDS	Neural Lazarillo (Nlaz)	***CG33126***	FBgn0053126	20%*	62%*	9e-13*	97%*	2HZQ, 5WY9(I-TASSER, Modeller)
HPGDS	Glutathione S transferase S1 (GST S1)	CG8938	FBgn0010226	36%	60%	3e-37	98%	^¥^1M0U (Modeller)
PTGIS	CYP450-4D2	***CG3466***	FBgn0011576	23% 26%*	44% 72%*	0.015 3e-70*	34% 93%*	3NXU, 4LXJ (I-TASSER, Modeller)
PTGES	Microsomal glutathione S-transferase-like (MGST-like, isoform A)	CG1742	FBgn0025814	36%	54%	2e-24	97%	4AL0 (Modeller)
PTGES2	Suppressor of Ref(2)p (SupRef(2)p)	CG4086	FBgn0004465	42%	62%	3e-70	73%	1Z9H (Modeller)
PTGES3	CG16817, isoform A	CG16817	FBgn0037728	27%	45%	6e-12	83%	2KMW, 1EFJ (Modeller; I-TASSER)
TBXAS1	CG3616	CG3616	FBgn0015040	31%	49%	6e-69	97%	3NXU, 4LXJ (I-TASSER)
AKR1A1, AKR1B1, AKR1C3	CG6084, isoform D	CG6084	FBgn0086254	49%	68%	5e-94	97%	1AH4 (Modeller)
CBR1	Carbonyl reductase (CBR, isoform B)	CG11200	FBgn0034500	30%	45%	8e-19	95%	4NBV, 3BHJ (Modeller, I-TASSER)
ALOX5, ALOX12, ALOX12B, ALOX15	None identified	N/A	N/A					
ALOX5AP	CG33177	***CG33177***	FBgn0053177	33%*	82%*	6e-20*	88%*	4AL0,2Q7M (Modeller, I-TASSER)
LTA4H	CG10602, isoform C	CG10602	FBgn0032721	44%	62%	4e-179	99%	4GAA (Modeller)
LTC4S	CG33178, isoform A	***CG33178***	FBgn0053178	28%*	61%*	4e-4*	84%*	4AL0, 2PNO (Modeller, I-TASSER)
GGT1	Gamma-glutamyl transpeptidase (GGT, isoform A)	CG6461	FBgn0030932	45%	58%	4e-133	92%	2E0W(Modeller)
DPEP1	CG6154, isoform C	CG6154	FBgn0039420	49%	65%	3e-125	88%	1ITU (Modeller)
HPGD	CG18814	CG18814	FBgn0042137	34%	53%	9e-35	76%	2GDZ (Modeller)
GPX1	PHGPx, isoform A	CG12013	FBgn0035438	35%	52%	6e-32	90%	2F8A (Modeller)
CPA1	CG18585, isoform A	CG18585	FBgn0031929	35%	55%	9e-82	98%	2V77 (Modeller)

Note: Statistics for each identified candidate gene are based on BLASTP and/or JACKHMMER results (when marked with an asterisk; the percentage identity and similarity refer to the match to the HMM profile and not the actual sequence) and are current as of November 2018. The candidates are denoted in the Annotation identifier column as high-confidence (regular font), mid-range (underlined), and distant (***italicized and bold font***). Gene annotation IDs (distinct from gene symbols) are represented in a common way: a species-specific 2 letter prefix followed by a four or five digit integer. CG is the 2-letter prefix for *D*. *melanogaster* for protein-coding genes.

^¥^The template 1M0U is the crystal structure for a fragment of this protein (position 48–249). The N-terminal region was built using MODELLER’s *ab initio* methods.

### Identification of potential eicosanoid synthesis enzymes in *D*. *melanogaster*

We used several different sequence analysis algorithms to identify candidate *Drosophila* orthologs for each human enzyme. Specifically, each human enzyme reference sequence was queried against a subset of the *D*. *melanogaster* NCBI non-redundant sequence database using PSI-BLAST, DELTA-BLAST or JACKHMMER [42–44]. For this, each human enzyme reference sequence was queried against a subset of the *D*. *melanogaster* NCBI non-redundant sequence database. DELTA-BLAST is a more sensitive variant of the traditional BLAST algorithm that incorporates domain information from pre-constructed position specific scoring matrices (PSSMs) in order to improve detection of homology [42]. JACKHMMER is an alternative sequence analysis and alignment tool that operates in an iterative fashion by generating a profile hidden Markov model (HMM) for a query protein, searching the target sequence database, and then updating the HMM to include sequences that score above a given cut-off value [44]. For this study, specialized BLAST and HMMer algorithms were leveraged as part of a combined approach to identify candidates which may be remote homologs for the human eicosanoid synthesis enzymes.

Each search was conducted with three iterations using the default parameters. *D*. *melanogaster* candidates with statistically significant full-length alignments to the query were selected after each round. These candidates were then analyzed using the same secondary structure and domain analysis tools as discussed above. Each candidate was then aligned against the previously generated MSA (containing the human enzyme and known/predicted orthologs) in order to screen out hits that lacked required functional residues and/or those with substantially dissimilar secondary structure or domain architecture.

### *In silico* modeling of *D*. *melanogaster* candidates

Full-length three-dimensional models were generated for each top-scoring *D*. *melanogaster* candidate protein thus identified using the HHpred, MODELLER, LOMETS and I-TASSER software packages [45–48] HHpred performs sensitive searches to detect protein homology, function, and structure prediction based on HMM–HMM comparison and uses MODELLER to create the models. MODELLER, a homology modeling tool, predicts the tertiary structure of a protein based on satisfaction of spatial restraints derived from sequentially similar templates whose structure is known (*i*.*e*., a sequence-sequence comparison). In contrast, I-TASSER utilizes a multi-threading algorithm (*i*.*e*., a sequence-structure comparison) to identify suitable templates followed by an iterative template fragment assembly approach based on replica-exchange Monte Carlo simulations to generate the full length model; unaligned primary sequence regions are built using *ab initio* methods. LOMETS is a meta-server that generates and ranks modeling results produced using several alternative threading algorithms which are executed in parallel. The accuracy of homology modeling depends largely on the target-template alignment, with ≥40% sequence identity generally yielding high quality models and 25% sequence identity being the lower threshold for an acceptable model [49]. However, threading based modeling tools are generally less sensitive to sequence identity differences and some packages (e.g., I-TASSER) are known to generate high quality models using templates sharing as little as 20–30% sequence identity with the target sequence [50,51].

Each *D*. *melanogaster* candidate sequence was searched against the Protein Data Bank database of published structures [52] using PSI-BLAST and JACKHMMER [43,44]. Potential templates with ≥30%, full-length sequence identity were aligned against the candidate using PROMALS3D, MAFFT, and T-Coffee [35,53,54] and evaluated. If a suitable single full-length template was not found, either due to a lack of coverage or low sequence identity within the aligned range, I-TASSER was used to generate a composite model based on several templates. In each case, the initial model was used to generate a refined full atomic model using ModRefiner, which also optimizes sidechain placement [55]. All of the refined models were then evaluated using the ProSa, ProQ2 and MODFOLD4 servers [56–58]. Loops and unstructured N- and C-terminal regions in these models were optimized using *ab initio* methods (e.g., MODELLER’s loop routines or QUARK), refined using ModRefiner and reevaluated as necessary [48,59]. Unsuitable models were rejected and rebuilt using alternatives templates according to the preceding protocol. Models were analyzed using the surface property tools in the Chimera and PyMol visualization and structural modeling packages, in addition to online tools including COFACTOR, and COACH [60–63]TM-Score was used to perform structural comparisons of the models with the known target human enzymes [64].

## Results

### Identification and characterization of candidate eicosanoid synthesis enzymes in *D*. *melanogaster*

A purely sequence-based analysis of the *D*. *melanogaster* genome (Release 6.04, February 24th, 2015) using traditional sequence analysis tools (*e*.*g*., BLASTP) failed to identify potential orthologs for some of the human eicosanoid synthesis enzymes. However, using a more sensitive approach based on iterative HMMER searches, we uncovered potential *D*. *melanogaster* candidates that may function as eicosanoid synthesis enzymes. A majority of these candidates were shown to adopt a highly similar tertiary structure with the human target enzymes. Template based modeling approaches produce models that are structurally similar to the templates used, so the modeled enzymes were validated using evaluation techniques that confirm that the primary sequence is compatible with its modeled three dimensional structure. To rule out potential false positives, the structure was evaluated carefully for conservation of the necessary catalytic residues in a structural context as well. A summary of our findings is provided in Table 1.

#### Group 1: The high scoring matches

Eight putative orthologs identified in this search have particularly high confidence matches: CG1742, CG4086, CG6084, CG10602, CG6461, CG6154, CG12013 and CG18585. These proteins share at least 30% sequence identity with the human enzyme, possess the same overall fold, and display conservation of one or more key functional residue needed for activity.

The first gene in this set, CG1742 (Microsomal glutathione S-transferase (MGST) -like, isoform A, NP_524696.1), encodes a protein of 152 residues and is predicted to have a single domain, Membrane Associated Proteins in Eicosanoid and Glutathione metabolism (MAPEG, Pfam ID: PF01124) spanning residues18 to 148 amino. This gene has also been identified in a bioinformatics screen for MAPEG family member [65]. This protein displays 36% identity and 54% similarity to the sequence of human prostaglandin E synthase (PTGES, NP_004869.1). The human PTGES (PS-1) is an integral membrane protein that operates as a homotrimer and is known to catalyze the oxidoreduction of prostaglandin endoperoxide H_2_ (PGH_2_) to prostaglandin E_2_ (PGE_2_) [66]. PTGES is characterized by the presence of a single MAPEG domain, which spans the region 16–146. PTGES (*e*.*g*., PDB ID: 4AL0; [67]) and is characteristic of several proteins belonging to the MAPEG family which includes MSGT and PTGES proteins [68]. This sequence is annotated as “MSGT-like” but the predicted structure of CG1742 is nearly identical and displays an RMSD 1.15 Å over 152 residues when superimposed with PTGES. PTGES, earlier referred to as MGST1-L1[69] is a glutathione-binding protein, and residues R38, R70, E77, R110, Y117, R126, and Y130 of human PTGES are associated with enzymatic activity as well as binding to glutathione [66,70]. Structural superposition reveals that CG1742 has a fully conserved set of these key functional residues, (R40, R71, E78, R111, Y118, R128, and F132). With respect to the last residue, a tyrosine to phenylalanine substitution is likely to be functionally equivalent as phenylalanine and tyrosine have side chains with a single aromatic ring of similar volume. Phenylalanine differs only in that it lacks the hydroxyl group in the ortho position on the benzene ring. Since previous studies suggest that the aromatic ring of Y130 is likely important for PGH2-binding, an analog for this structural element is provided by F132 [70]. Details of modeled fly candidate PTGES and its comparison with human PTGES are provided in S1 Fig and a similar format is followed for all the candidate orthologs identified.

The second gene in this set, CG4086 (Suppressor of ref(2)P sterility (Su(P)), NP_524116.2) encodes a 417 residue protein. CG4086 displays 42% identity and 62% similarity to the sequence of human prostaglandin E synthase 2 (PTGES2, NP_079348.1). PTGES2 is membrane-associated, as opposed to PTGES which is an integral membrane protein, though both catalyze the same reaction [71]. Despite the functional similarities, PTGES2 possesses a substantially different domain architecture and tertiary structure. The human PTGES2 possesses a GST-N3 domain (Pfam ID: PF13417) spanning amino acids 104–175 and a GST-C3 (Pfam ID: PF14497) domain spanning amino acids 201–368. CG4086 shares a highly similar domain architecture with the same domains spanning amino acids 125–184 and 248–396 respectively. PTGES2’s catalytic activity requires an essential residue, C110 [72]. The crystal structure of truncated PTGES2 has been published (PDB ID: 1Z9H; [73]), with the N-terminal membrane-associated region omitted (residues 1 to 99). Superposition of this truncated structure against the predicted structure of CG4086 reveals an RMSD of 0.814 Å over 377 residues. CG4086’s C133 overlaps with PTGES2’s C110, suggesting a match for this catalytic residue (S2 Fig).

The third gene in this set, CG6084, encodes a 316 amino acid protein that is a high confidence match for the human Alcohol dehydrogenase NADP(+) (AKR1B1”, NP_001619.1). This enzyme, along with enzymes encoded by the related genes in this family (*e*.*g*., AKR1B15 and AKR1C3), catalyzes the NADPH-dependent reduction of a variety of aromatic and aliphatic aldehydes to their corresponding alcohols. In particular, AKR1B1 and AKR1B15 are known to function as prostacyclin F synthases, converting prostaglandin endoperoxide H_2_ (PGH_2_) to prostaglandin F_2α_ (PGF_2α_) [74,75]. CG6084 and AKR1B1 share identical domain architectures [a single aldo/keto reductase family (Pfam ID: PF00248) domain], as well as 49% identity and 68% similarity at the level of primary structure. Three residues required for AKR1B1’s catalytic activity are Y48, K77 and H110 [76,77]. CG6084 possesses analogs for this triad of residues in the form of Y50, K79 and H112. In addition, the structural overlap of the predicted structure for CG6084 and human AKR1A1 (PDB ID: 2ALR; [78]) displays an RMSD of 0.847 Å over 316 residues (S3 Fig).

The fourth gene in this set, CG10602 encodes a protein with 613 residues that displays 44% identity and 62% similarity to human leukotriene A4 hydrolase (LTA4H, NP_000886.1). LTA4H is an epoxide hydrolase that catalyzes the hydrolysis of the epoxide LTA4 to the diol, LTB4. This catalytic activity requires the presence of a zinc ion, which is coordinated by the catalytic triad of H296, H300 and E319. Residues E297, D376 and Y384 are also reportedly essential for efficient catalysis [79]. LTA4H’s domain architecture consists of Peptidase family M1 (Pfam ID: PF01433) spanning 13–387 and a Leukotriene A4 hydrolase, C-terminal domain (Pfam ID: PF09127) spanning amino acids 464–608. CG10602 shares an identical domain organization as well as the critical residues at H293, H297, E316 (the zinc triad) and E294, D374 and Y382. The tertiary structure similarity is readily apparent, as evidenced by a superposition of human LTA4H (PDB ID: 3B7U; [80]) and the predicted structure of CG10602, which results in an RMSD of 0.891 Å over 611 residues (S4 Fig).

The fifth gene in this set, CG6461, encodes a 579 residue protein (Gamma-glutamyltranspeptidase, NP_573303.1) that shares 45% identity and 58% similarity to human Gamma-glutamyltranspeptidase 1 (GGT1, NP_001275762.1). GGT1 cleaves the gamma-glutamyl bond of extracellular glutathione (gamma-Glu-Cys-Gly), glutathione conjugates, and other gamma-glutamyl compounds. With respect to eicosanoid synthesis, GGT1 catalyzes the conversion of leukotriene C4 (LTC4) to leukotriene D4 (LTD4). GGT1 operates as a heterodimer consisting of small and large subunits which are produced from the same precursor that undergoes autocatalytic cleavage to produce the mature form of the enzyme. The only reported catalytic residue is T381, which corresponds to T382 in CG6461. GGT1 residues R107, T399 and E420 are believed to play a role in glutamate binding [81]. CG6461 possesses putative analogs for each of these residues, namely R107, T400 and E421, respectively. CG6461 and GGT1 also share identical domain architecture, with Gamma-glutamyltranspeptidase (Pfam ID: PF01019) domains spanning the majority of each protein. Superimposed, the human structure (PDB ID: 4GDX; [82]) and CG6461’s predicted structure displays an RMSD of 1.126 Å over 569 residues (S5 Fig).

The sixth gene in this set, CG6154 (“DPEP,” NP_733146.2), encodes a 434 residue metallopeptidase that shares 49% identity and 65% similarity with human Dipeptidase, renal (DPEP1, NP_004404.1). Both enzymes contain a single Membrane dipeptidase (Pfam ID: PF01244) domain spanning the majority of the protein. DPEP1 is known to regulate leukotriene activity by catalyzing the conversion of leukotriene D_4_ (LTD4) to leukotriene E_4_ (LTE4) using zinc as a cofactor. To that end, the residues H36, D38, E141, H214 and H235 are reported to be essential for coordinating the zinc ions required for catalysis [83]. Aligned against CG6154, these residues correspond to H71, D73, E184, H257 and H278. This high degree of similarity extends to the tertiary structure, as the superimposed structure of human DPEP1 (PDB ID: 1ITQ; [83]) and the predicted structure of CG6154 display an RMSD of 0.523 Å over 411 residues (S6 Fig).

The seventh gene in this set, CG12013 (“glutathione peroxidase,” NP_728870.1), encodes a 169 residue selenoprotein that is 35% identical and 52% similar to human glutathione peroxidase 1 (GPX1, NP_000572.2) and both contain a single glutathione peroxidase domain (Pfam ID: PF00255). As part of the eicosanoid pathway, GPX-1 converts the 12(S)-HpETE produced by lipoxygenase-12 (12-LOX) into 12(S)-HETE [84]. GPX1 requires a selenocysteine residue (U49) for catalysis [85]. CG12013’s amino acid sequence indicates that a cysteine residue is present at the corresponding position in this enzyme. However, this residue may in fact be a selenocysteine, as this substitution is unlikely to be detected by sequencing alone. Similarly, the tryptophan and glutamine residue that complete the catalytic triad are also conserved. A structural alignment of human GPX1 (PDB ID: 2F8A; unpublished, Structural Genomics Consortium (SGC)) and the predicted structure of CG12013 reveals an RMSD of 0.699 Å over 169 residues (S7 Fig).

The eighth and final gene in this set, CG18585 (Carboxypeptidase A (CPA), NP_609132.1) encodes a 422 amino acid protein that is 35% identical and 55% similar to human Carboxypeptidase A1 (CPA1, NP_001859.1). CPA1 is a metallocarboxypeptidase that requires a zinc atom as a cofactor for catalysis. CPA1 has been shown to convert the potent leukotriene C4 (LTC4) to the less potent leukotriene F4 (LTF4) by hydrolysis of an amide bond, suggesting that CPA1 serves to reduce inflammation [86]. The residues H179, E182, H306 and E380 are required for catalysis and coordinating zinc. These residues correspond to H178, E181, H305 and E382 in CPA. Aligned, the structure of human CPA1 (PDB ID: 3FJU; [87]) and the predicted structure of CG18585 display an RMSD of 0.753 Å over 419 residues (S8 Fig).

#### Group 2: The midrange candidates

Five candidates were identified in the search based on approaches focused more on structural similarity than underlying sequence conservation. Members of this set share at least 20% sequence identity with the human target but share similarity at the fold level and possess at least a partial set of conserved functional residue.

The first gene in this set, CG8938 (GST-S1, NP_725653.1) encodes a 249 residue glutathione-S-transferase enzyme, which displays 36% identity and 60% similarity to human Hematopoietic Prostaglandin D synthase (HPGDS, NP_055300.1). HPGDS, alternatively known as Prostaglandin-H2 D-isomerase, is a glutathione-requiring prostaglandin D synthase. HPGDS operates as a homodimer, which can optionally be activated by Ca^2+^ and Mg^2+^ to increase catalytic efficiency to 150% of the basal level. Coordination of the metallic ion ligands is coordinated by D93, D96 and D97 [88]. CG8938 and GST-S1 share an identical domain architecture, with an N-terminal GST-N domain (Pfam ID: PF02798) and a C-terminal GST-C domain (Pfam ID: PF00043). Similarly, CG8938 possesses matches for two of the three aspartic residues required for enhanced catalytic activity, at positions D139 and D143 (corresponding to D93 and D97 in HPGDS). At the corresponding position aligned to D96, CG8938 instead has an asparagine (N142). However, mutagenesis studies by Inouie *et al*. have shown that a D to N substitution at this same position in HPGDS actually increases PGD_2_ synthesis [88]. In addition to this sequence-based evidence, superposition of the predicted CG8938 structure and human HPGDS (PDB ID: 1IYI; [88]) reveals an RMSD of 1.045 Å over 160 residues (S9 Fig).

The second gene in this set, CG16817 (NP_649925.1) encodes a 184 residue protein, which displays 27% identity and 45% similarity to human Prostaglandin E synthase 3 (PTGES3, NP_006592.3). In contrast to PTGES1 and PTGES2 discussed above, PTGES3 is a cytosolic protein. However, all three enzymes share the same catalytic function, i.e., oxidoreduction of prostaglandin endoperoxide H_2_ (PGH_2_) to prostaglandin E_2_ (PGE_2_) [89]. PTGES3 and CG16817 share an identical domain architecture, with the bipartite CHORD-containing proteins and SGT1 (CS) Domain (Pfam ID: PF04969) spanning the N-terminus to the middle of the protein. The C-terminal region of PTGES3 is notable for showing a compositional bias towards aspartic acid and glutamic acid residues in the range spanning 108 to 160. Currently, only one crystal structure for PTGES3 has been published (PDB ID: 1EJF; [90]) and this structure is limited to a truncated version of the protein spanning residue 1 to 110, *i*.*e*., the acidic C-terminal region has not been crystalized. An alignment of PTGES3 and CG16817 shows that CG16817 also displays a compositional bias towards acidic residues at its C-terminus, though it is unclear what role this bias serves in either protein. We were unable to generate a full-length model of CG16817 due to a lack of any suitable template structure for the C-terminal acidic region. However, the truncated CG16817 model (residues 1 to 113) shows an RMSD of 0.823 Å compared to PTGES3 over 160 residues (S10 Fig).

The third gene in this set, CG11200 (Carbonyl reductase (CBR), NP_611471.1) encodes a 355 residue protein that displays 30% identity and 45% similarity to human carbonyl reductase 1 (CBR1, NP_001748.1). CBR1 is a NADPH-dependent reductase with broad substrate specificity, *e*.*g*., it can convert PGE_2_ to PGF_2α_. It has been reported that CBR1 binds to NADP via N90 and that Y194 serves as a proton acceptor for the reaction [91]. A corresponding match for both of these residues can be found in CG11200 at positions N154 and Y233. An alignment of human CBR1 (PDB ID: 3BHJ; [92]) against the predicted model for CG11200 shows an RMSD of 1.153 Å over 277 residues (S11 Fig).

The fourth gene in this set, CG18814 (NP_652673.2), encodes a 267 amino acid protein that displays 34% identity and 53% similarity to human 15-hydroxyprostaglandin dehydrogenase NAD(+)(HPGD, NP_000851.2). HPGD catalyzes the conversion of the 15-hydroxyl group of prostaglandins into a keto group, which strongly reduces the biologic activity of these molecules. As a result, HPGD is considered the primary enzyme responsible for degradation of prostaglandins. It has been reported that N91, S138, Q148 and Y151 are required for catalysis [93]. CG18814 residues N89, S137 and Y150 of CG18814 align with these catalytic residues and are oriented at overlapping or directly adjacent positions in close proximity in the structural alignment of the CG18814 model against the structure of HPGD. CG18814 lacks an obvious analog for Q148, which is located in a flexible loop region. However, CG18814 possesses a structurally-aligned loop region which contains Q142. Q148 and Q142 are located within approximately 5Å of each other and oriented similarly in a static superposition of the predicted model and crystal structure and may be functionally analogous. Moreover, both proteins have an identical domain architecture, with a short-chain dehydrogenase domain (Pfam ID: PF00106) spanning from the N-terminus to approximately residue 180. Superposition of human HPGD (PDB ID: 2GDZ; [93]) and the predicted structure of CG18814 reveals an RMSD of 0.715 Å over 266 residues (S12 Fig).

The fifth and final gene in this set, CG3616 (CYP450-9c1, NP_523850.1) encodes a cytochrome P450 oxidase, which displays 31% identity and 49% similarity to human Thromboxane A synthase (TBXAS1, NP_001052.2). TBXAS1 localizes to the endoplasmic reticulum membrane, where it catalyzes the conversion of PGH_2_ to thromboxane A_2_ (TBA_2_) [94]. TBXAS1 is categorized as a cytochrome p450 family (CYP450) enzyme based on sequence similarity. This categorization is illustrated by the fact that the only domain identified in TBXAS1 is a Cytochrome P450 domain (Pfam ID: PF00067) spanning most of the protein. CYP450 enzymes are highly conserved in eukaryotes, where they catalyze reactions in drug metabolism and synthesis of cholesterol, steroids and other lipids [95]. However, this ubiquitous nature of CYP450 enzymes makes it difficult to identify potential orthologs for any one specific enzyme within the family. CG3616 was selected as a candidate from the set of eight fly candidates with similar sequences after taking into account the initial sequence comparison, the transmembrane helix pattern (calculated using TOPCONS [96]), and the alignment score against a multiple sequence alignment of known or high confidence TBXAS1 orthologs. A structural superposition of a modeled human TBXAS1 (no structure available) with the predicted model of CG3616 showed an RMSD of 0.699 Å over 518 residues (S13 Fig).

#### Group 3: The most distant candidates

Many of the putative orthologs identified in *D*. *melanogaster*, although divergent in sequence from the human target, are similar enough that traditional methods for analyzing and aligning sequences can be utilized. However, we identified 6 sequences that could not be detected by or showed low sequence identity and/or low query coverage using traditional sequence alignment algorithms such as BLASTP and therefore, required more intensive analysis.

The search for a cyclooxygenase ortholog proved to be one of these difficult cases. As noted above, it is widely accepted that *D*. *melanogaster* lacks a cyclooxygenase ortholog. However, there have been sporadic reports of prostaglandin detected in *D*. *melanogaster* extracts over the years [27]. The human genome encodes three major cyclooxygenase isozymes via two loci. PTGS1 encodes COX-1 (a constitutive cyclooxygenase), whereas PTGS2 encodes COX-2 (which is inducible and expressed in a tissue-specific manner). A truncated and poorly characterized isoform of PTGS1, COX-3 is also expressed in certain cells, and reportedly demonstrates reduced prostaglandin synthesis activity relative to COX-1 [97]. COX-1 and COX-2 operate as homodimers and are characterized by two domains: An N-terminal EGF-like domain (Pfam ID: IPR000742) and an animal heme peroxidase domain (Pfam ID: PF03098). The former allows for association with the cell and nuclear membrane while the latter is responsible for the cyclooxygenase activity. The COX enzymes initiate the prostaglandin synthesis pathway by converting arachidonic acid, or other polyunsaturated fatty acid (PUFA) substrates, into the unstable intermediate PGG_2_ followed by PGH_2_. During this process, it has been reported that a small amount of the PUFA substrate is converted into a racemic mixture of 15-Hydroxyicosatetraenoic acids (*i*.*e*., 15-HETEs), which may be processed into lipoxins, a poorly understood class of anti-inflammatory eicosanoids. The COX-1 and COX-2 enzymes contain two active sites: a heme with peroxidase activity, responsible for the reduction of PGG_2_ to PGH_2_, and a cyclooxygenase site, where arachidonic acid is converted into the hydroperoxy endoperoxide prostaglandin G_2_ (PGG_2_) [6]. Extensive studies have been performed on the sheep ortholog of COX-1(NP_001009476.1; PDB ID: 1CQE), which is a proxy for the human orthologs [98]. These studies reveal that cyclooxygenase activity is mediated by Y385, which forms a radical capable of abstracting a hydrogen from the PUFA substrate (*e*.*g*., carbon-13 of arachidonic acid). H207 and H388 are also required for the dual peroxidase and cyclooxygenase functions of these enzymes [99,100]. Other functional residues identified in the literature include R120, Q203, V349, and S530 [101]. R120 interacts with C-1 of the PUFA substrate, arachidonic acid [102]. Q203 is conserved among mammalian COX enzymes, though the function of this residue is currently unknown. V349 is believed to play a role in substrate specificity; mammalian COX enzymes share this residue and show a substrate preference for arachidonic acid, while invertebrate COX enzymes that have a leucine at this position display specificity for linoleic acid [103] and S530 acetylation is the basis for COX-2 inhibition by aspirin [99].

**Fig 2:**
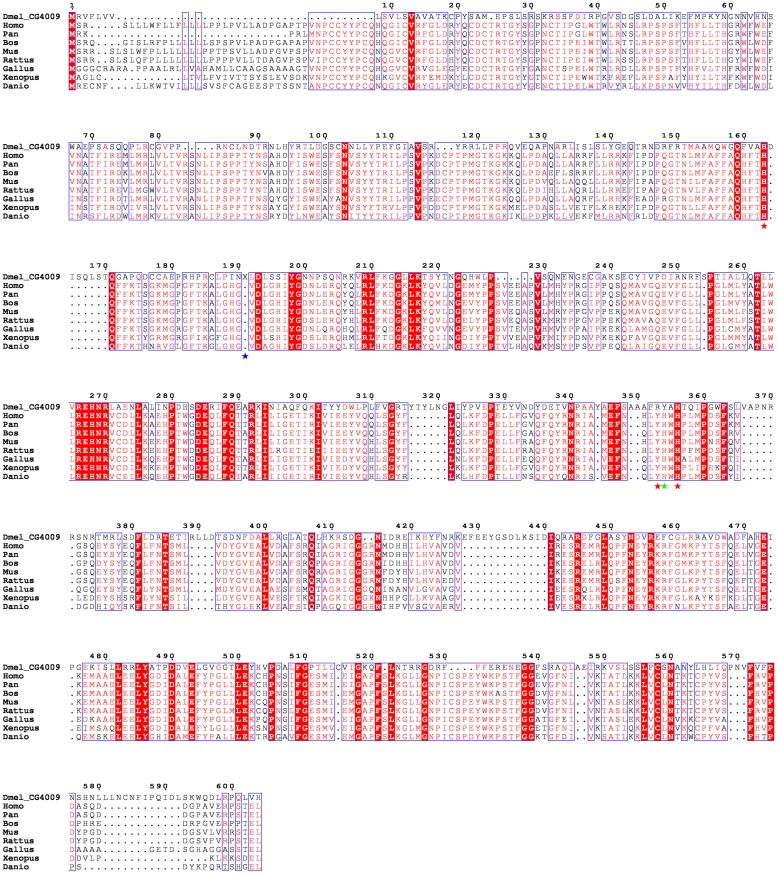
A multiple sequence alignment of the *D*. *melanogaster* candidate CG4009 with human PTGS1 and known or high confidence predicted orthologs. Red asterisks below the MSA denotes conserved key catalytic residues. The green asterisk marks the putative analog for the tyrosine. The blue asterisk denotes an insertion of 42 residues in CG4009 and reads X in the alignment.

Although an initial screen of *D*. *melanogaster* using BLASTP reported similar sequences with local matches, further investigation was needed to confirm if these candidates possessed the requisite features of a COX enzyme. Iterative HMMER searches confirmed four potential candidates: CG4009 (NP_650588.2), CG6969 (Cardinal, NP_651081.1), CG7660 (Pxt, NP_650648.3) and CG3477 (Pxd, NP_996223.1). Each of these candidates was added to a previously generated multiple sequence alignment containing PTGS1 and known or high confidence predicted orthologs using MAFFT as illustrated for CG4009 in Fig 2. The presence or absence of functional residues at or near aligned positions was noted. Similarly, the candidates were analyzed to determine the secondary structure and domain architecture of each protein. Following this filtering step, only three proteins remained as viable candidates for further study: CG4009, CG6969, and CG7660. CG7660 has been shown to demonstrate COX-like activity that plays an important role in Drosophila reproduction [29]. All three candidates appear to have the requisite catalytic triad and share a similar domain architecture, though they each lack the N-terminal EGF-like domain. Putative analogs for the H206, Y384 and H387 catalytic triad appear to be present on CG4009 at H163, Y399, H401 and on CG7660 at H222, Y564, H568, respectively.

Structural models of CG4009, CG6969, and CG7660 were built and validated as described above. PTGS1 (PDB ID: 3N8V; [104]) and the predicted structure for CG4009 both display a cyclooxygenase fold, and the predicted catalytic domains are superimposable with an RMSD of 2.660 Å over 599 residues (S14 Fig). CG7660 and CG6969 each display a similar tertiary structure and with predicted catalytic domains that overlap with PTGS1 with an RMSD of 2.810 and 2.942 Å, respectively. The predicted structures differ from PTGS1 at their N-termini, which is expected given the absence of the EGF-like domain, though the CG4009 structure is marginally closer. However, these three structures present the necessary catalytic residues in a similar orientation as in PTGS1 and so likely possess cyclooxygenase activity.

The search for a potential ortholog for PTGDS also proved to be difficult. PTGDS is a second prostaglandin-D-synthase, which operates in conjunction with the isozyme HPGDS (discussed previously). BLAST-based searching failed to identify any *D*. *melanogaster* candidates for the human enzyme (NP_000945.3). However, iterative HMMER searches suggested CG33126 (Neural Lazarillo (NLaz), NP_787960.1) merits further scrutiny despite having only 12% identity and 31% similarity to PTGDS based on a structure based sequence alignment. Both proteins share similar domain architecture, namely a Lipocalin domain (Pfam ID: PF00061), which is known to take the form of a beta barrel structure. Structural superposition revealed that human PTGDS (PDB ID: 5WY9; unpublished) and the CG33126 model overlap with an RMSD of 2.084 Å over 190 residues and that CG33126 has an aligned match for PTGDS’s catalytic cysteine (C65) at position C67 [105]. CG33126 contains an N-terminal alpha helix that is absent from PTGDS, however this helix is predicted to be a signal peptide. PTGDS contains a similar N-terminal signal peptide that is cleaved during processing *i*.*e*., omitted from the 5WY9 crystal structure (S15 Fig).

Prostacyclin synthase (PTGIS) is another enzyme that does not have a clear ortholog in *D*. *melanogaster*. PTGIS catalyzes the isomerization of PGH_2_ to prostacyclin, the only prostaglandin with a bicyclic ring structure [106]. PTGIS encodes a 500 amino acid enzyme characterized by a cytochrome p450 family domain ranging from position 30 to 494. The N-terminus is predicted to contain a 200 amino acid signal sequence. BLAST and iterative HMMER searches reveal multiple high-scoring full length matches among the various CYP450 enzymes encoded in the *D*. *melanogaster* genome. However, CG3466 (CYP450-4d2, NP_525043.1) was identified as the top candidate based upon similarity of its secondary structure profile. CG3466 possesses an aligned match for PTGIS’s heme axial ligand (C441) at position C449. PTGIS (PDB ID: 2IAG; [107]) and the modeled CG3466 are superimposable with an RMSD of 1.213 Å over 500 residues, despite sharing only 14% identity and 30% similarity at the sequence level gauged from a structure based sequence alignment (S16 Fig).

Leukotriene C4 synthase (LTC4S), is a 150 amino acid enzyme that catalyzes the conjugation of leukotriene A4 with reduced glutathione to form leukotriene C4. BLAST searches against *D*. *melanogaster* fail to identify an obvious ortholog. However, an iterative HMMER search identified the 165 amino acid protein CG33178 (NP_788904.1) as a potential candidate. The two proteins share full length identity of 18% and similarity of 31% discerned from a structure based sequence alignment and have an identical domain architecture in the form of a single MAPEG domain (Pfam ID: PF01124) spanning the bulk of the protein. Studies of the crystal structure of LTC4S (PDB ID: 2PNO; [108]) have revealed that it is an integral membrane protein composed of four transmembrane helices and that it functions as a homotrimer. The predicted model of CG33178 displays a similar fold, consisting of a bundle of four alpha helices, and it superimposes on 2PNO with an RMSD of 1.257 Å over 150 residues (S17 Fig). CG33178 also displays potential equivalents for each of LTC4S’s catalytically relevant residues (R30, R31 and R104) at positions R51, R53 and R139.

Arachidonate 5-lipoxygenase-activating protein (ALOX5AP, NP_001620.2), the final target protein in this set, is required for leukotriene synthesis by arachidonate 5-lipoxygenase (ALOX5). It has been reported that ALOX5AP functions by binding ALOX5 to the membrane and loading polyunsaturated fatty acid substrates (e.g., arachidonic acid) onto ALOX5 for conversion into leukotriene A_4_. Like LTC4S above, ALOX5AP is a MAPEG family protein that functions as a membrane-associated homotrimer. BLAST searches for a potential ortholog do not identify a clear match in *D*. *melanogaster*, though iterative HMMER searches reveal CG33177 and CG33178 as potential candidates. Both are MAPEG family proteins with highly similar sequences and predicted tertiary structures. CG33178 displays greater sequence similarity to LTC4S and analogs for the catalytically relevant residues and so was assigned as the top candidate for LTC4S. As a result, CG33177 was selected as the top candidate for a potential ortholog for ALOX5AP. The CG33177 structural model displays 13% identity and 28% similarity with human ALOX5A based on a structural overlap (PDB ID: 2Q7M; [109]) with an RMSD of 1.024 Å over 161 residues (S18 Fig).

## Discussion

**Fig 3:**
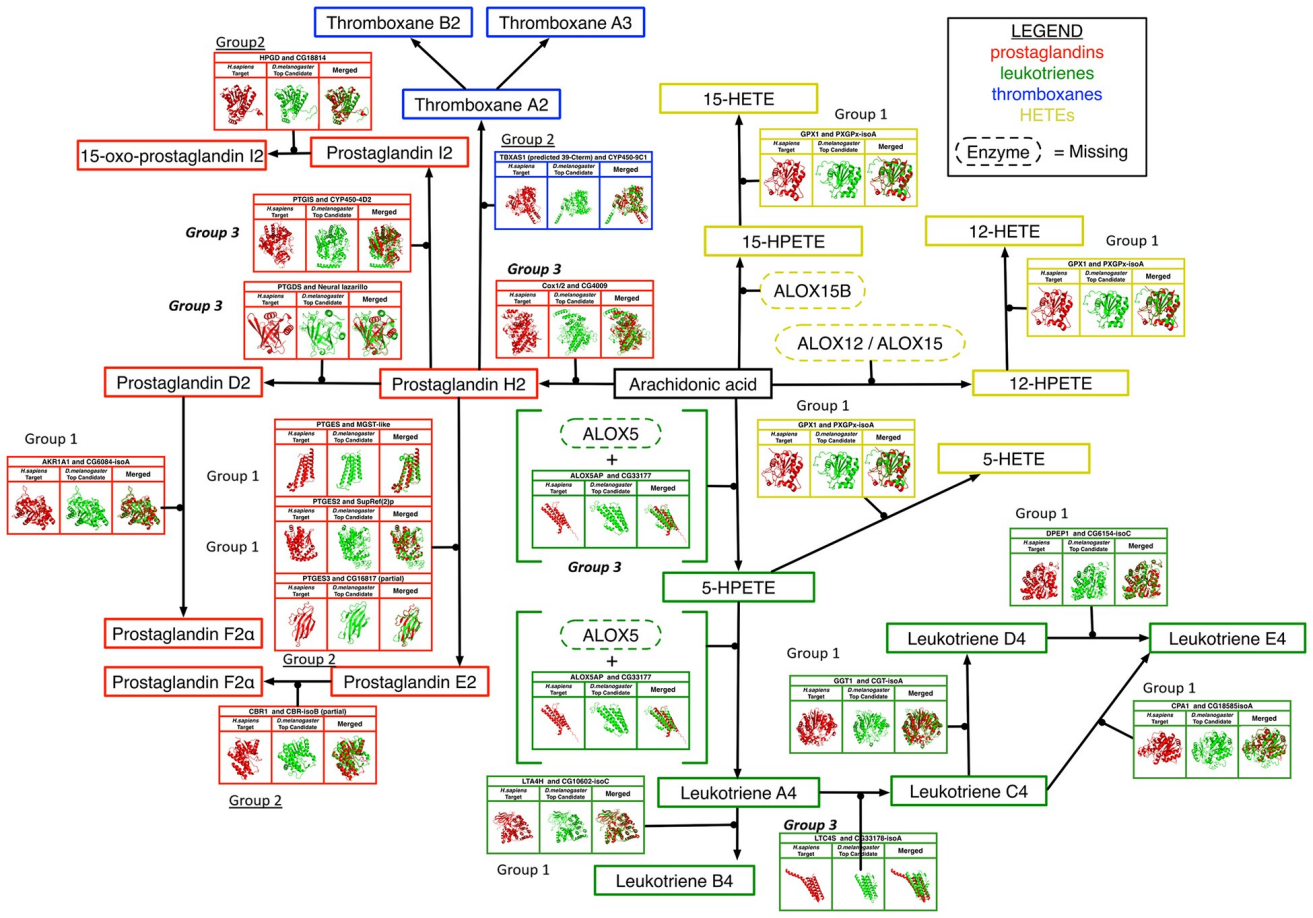
A theoretical eicosanoid synthesis pathway in *D*. *melanogaster*. A graphical overview of fly candidates in the putative pathway shown alongside the human enzymes as well as superposed with it. Boxed names represent the intermediates or end products (Red: Prostaglandins; Blue: Thromboxanes; Green: Leukotrienes; Yellow: HETEs). Enzymes for which orthologs could not be identified are represented by dashed ovals. The three candidate groups are marked next to the enzymes in a similar manner as Table 1: high-confidence (regular font; Group 1), mid-range (underlined; Group 2), and distant (***italicized and bold font; Group 3***).

A schematic of a putative *D*. *melanogaster* eicosanoid synthesis pathway based on our analysis is shown in Fig 3. Notably, the pathway appears to account for a full complement of prostaglandin synthesis enzymes. A functional thromboxane synthesis pathway may also be present, as potential orthologs for thromboxane A synthase have been identified. However, given the structural and sequence similarity of TBXA synthase to the numerous functionally unrelated cytochrome P450 oxidase in the *D*. *melanogaster* genome, speculation must be reserved pending experimental validation.

The existence of a leukotriene synthesis arm of the pathway could not be fully resolved since a thorough search of the *D*. *melanogaster* genome has failed to identify a potential lipoxygenase, which is critical for the initial processing of the PUFA substrate into leukotriene intermediates. Interestingly, potential orthologs exist for each of the downstream leukotriene processing enzymes. Based upon our current understanding of the mammalian pathway, a functional lipoxygenase is necessary for HETE intermediates and the corresponding final products (*e*.*g*., lipoxins and other non-canonical eicosanoids) [110].

Our data challenges that view that *D*. *melanogaster* lacks a functional eicosanoid synthesis pathway [110–112] and suggests that it may in fact possess potential orthologs for some or majority of the eicosanoid synthesis enzymes. These candidates may have thus far eluded detection because of high sequence divergence compared to the mammalian enzymes. However, the modeling results from our study, suggest that these candidates share the same overall fold and matches for the known or predicted catalytic residues. The prediction of eicosanoid synthesis enzymes may explain the *in vivo* effects of aspirin and prostaglandins in oogenesis [29] and possibly the existence of eicosanoid-like compounds in *D*. *melanogaster* [27,28,113]. Our results however raise many questions: (a) What are the exact roles of individual enzymes; do they catalyze the reactions their structures predict they should? (b) What are the intermediate and final end products of the pathway? (c) What are the principal physiological events these intermediate and final biosynthetic products control and how similar are the signaling mechanisms by which their effects are mediated in cells and tissues?

The apparent absence of lipoxygenase may be explained in part by the multiple cyclooxygenase genes in the *D*. *melanogaster* genome where one of more of these may only or also possess lipoxygenase like activity. Dual function cyclooxygenases can generate prostaglandins and lipoxygenase products (e.g. HPETEs). For example, mammalian cyclooxygenases can produce limited amounts of 11- and 15-HPETE as a by-product in addition to PGH [114–116] and aspirin-acetylated COX-2 has been shown to produce 15R-HETEs [117]. Similar activity by one of the putative *D*. *melanogaster* COX orthologs could provide upstream processing needed for 11- and 15-HPETE, though this fails to provide the 5-HPETE intermediate needed for downstream leukotriene synthesis. Alternatively, 5-HETEs may be produced using an unconventional mechanism by the COX candidates or perhaps by an unidentified enzyme.

The existence of structurally conserved downstream leukotriene synthesis enzymes suggests that the upstream intermediates are likely present in some form. *Drosophila* may possess a CYP450 enzyme capable of synthesizing 5-HETE; lipoxygenase-like CYP450s have been identified that produce 5-, 8-, 9-, 11-, 12- and 20-HETEs [118,119]. Recent studies have reported on bacterial lipoxygenases which are highly divergent from animal and plant lipoxygenases, both in terms of overall sequence conservation and the presence of expected catalytic residues [120]. There is also a possibility that *D*. *melanogaster* and other insect expresses a functional 5-LOX that differs radically from the canonical profile of a lipoxygenase.

The existence of a functional eicosanoid synthesis pathway requires both the presence of the requisite synthetic enzymes and suitable substrates. The mammalian eicosanoids are primarily derived from 20-carbon PUFAs (arachidonic acid, eicosapentaenoic acid and dihomo-γ-linolenic acid), as well as from 18-carbon PUFAs (γ-linolenic acid, α-linolenic acid and linoleic acid). In the vertebrate pathway, the 18-carbon fatty acids linoleic acid and α-linolenic acid can be processed into the 20-carbon PUFAs arachidonic acid and eicosapentaenoic acid, respectively, via a three-step process involving a Δ6 desaturase, an elongase, and, a Δ5 desaturase. These 20-carbon PUFAs are then capable of being processed by COX or LOX to generate the canonical eicosanoids. 18-carbon PUFAs (*e*.*g*., linoleic acid) may also be processed by 15-LOX into 9- and 13-HODEs, a subfamily of non-canonical eicosanoids.

According to previous studies *Drosophila* appear to lack homologs for the mammalian Δ5 and Δ6 desaturases based upon sequence analyses, and so it should be unable to synthesize 20–22 carbon PUFAs from essential fatty acid precursors [112]. Furthermore, flies raised in the absence of 20-carbon PUFAs for several generations remain healthy, implying that 20-carbon PUFAs are non-essential [112]. Thus, it is generally accepted that *Drosophila* rely on PUFAs obtained from its vegetarian diet, which is generally limited to the essential 18-carbon fatty acids linoleic acid (18:2n-6) and α-linolenic acid (18:3n-3). Based on the current understanding that *D*. *melanogaster* lacks Δ5 and Δ6 desaturase activity, it would be expected that the 18-carbon PUFAs obtained from its diet are processed into HODEs as opposed to eicosanoid derivatives of the 20-carbon PUFAs. Recent studies have validated this hypothesis, and confirmed the presence of 9- and 13-HODEs in *D*. *melanogaster* extracts (Govind lab, unpublished work); [121]. However, given the reports of prostaglandin-like molecules in *D*. *melanogaster* extracts, it is probable that additional lipid processing occurs to produce compounds similar to the canonical eicosanoids but the biosynthetic components have not been identified yet. Further computational and experimental work is needed to fully resolve these issues.

Altogether, the present study suggests that *D*. *melanogaster* may possess a functional equivalent for the mammalian eicosanoid synthesis pathway. Our results highlight the need for customizing advanced prediction methods to identify orthologs, and to build structural models that predict the requisite biochemistry for function. These findings, combined with other recent biochemical studies, suggest the possibility that *D*. *melanogaster* likely utilizes eicosanoid signaling. However, further experimental studies will be needed to be address the exact role and physiological context of these biosynthetic enzymes. The importance of our study lies in opening up this powerful model organism for the study of eicosanoid signaling, in the context of inflammation as well as non-inflammation related physiology.

## Supporting information

S1 Fig Sequence and structural details of the modeled fly PTGES candidate. A. Domain architecture of PTGES and CG1742 and known/predicted functional residues B. Pairwise alignment of CG1742 and 4AL0 generated from structural superposition showing shared secondary structure elements and known/predicted functional residues (marked with a red asterisk) C. Pairwise alignment of CG1742 and 4AL0 generated from structural superposition with conserved residues highlighted using the physiochemical color scheme (CLUSTALX) D. Validation of the CG1742 model: ProQ2 quality score mapped to a 3D model of CG1742 (left); ProSA global quality score ranking (middle) and per-residue quality graph (right) E. PTGES (4AL0, cyan-blue) superimposed on the predicted structure of CG1742 (green-red) with potential matches for conserved functional residues highlighted F. Summary of features shared by PTGES and potential *D*. *melanogaster* ortholog CG1742.
(PDF)

S2 Fig Sequence and structural details of the modeled fly PTGES2 candidate. A. Domain architecture of PTGES2 and CG4086 and known/predicted functional residues B. Pairwise alignment of CG4086 and 1Z9H generated from structural superposition showing shared secondary structure elements and known/predicted functional residues (marked with a red asterisk) C. Pairwise alignment of CG4086 and 1Z9H generated from structural superposition with conserved residues highlighted using the physiochemical color scheme (CLUSTALX) D. Validation of the CG4086 model: ProQ2 quality score mapped to a 3D model of CG4086 (left); ProSA global quality score ranking (middle) and per-residue quality graph (right) E. Truncated PTGES2 (1Z9H, cyan-blue) superimposed on the predicted structure of CG4086 (green-red) with potential matches for conserved functional residues highlighted F. Summary of features shared by PTGES2 and potential *D*. *melanogaster* ortholog CG4086.
(PDF)

S3 Fig Sequence and structural details of the modeled fly Prostacyclin F synthase candidate. A. Domain architecture of AKR1A1 and CG6084 and known/predicted functional residues B. Pairwise alignment of CG6084 and 2ALR generated from structural superposition showing shared secondary structure elements and known/predicted functional residues (marked with a red asterisk) C. Pairwise alignment of CG6084 and 2ALR generated from structural superposition with conserved residues highlighted using the physiochemical color scheme (CLUSTALX) D. Validation of the CG6084 model: ProQ2 quality score mapped to a 3D model of CG6084 (left); ProSA global quality score ranking (middle) and per-residue quality graph (right) E. AKR1A1 (2ALR, cyan-blue) superimposed on the predicted structure of CG6084 (green-red). RMSD: 0.847 Å with potential matches for conserved functional residues highlighted F. Summary of features shared by Prostacyclin F synthase and potential *D*. *melanogaster* ortholog CG6084.
(PDF)

S4 Fig Sequence and structural details of the modeled fly LTA4H candidate. A. Domain architecture of LTA4H and CG10602 and known/predicted functional residues B. Pairwise alignment of CG10602 and 3B7U generated from structural superposition showing shared secondary structure elements and known/predicted functional residues (marked with red asterisks) C. Pairwise alignment of CG10602 and 3B7U generated from structural superposition with conserved residues highlighted using the physiochemical color scheme (CLUSTALX) D. Validation of the CG10602 model: ProQ2 quality score mapped to a 3D model of CG10602 (left); ProSA global quality score ranking (middle) and per-residue quality graph (right) E. LTA4H (3B7U, cyan-blue) superimposed on the predicted structure of CG10602 (green-red) with potential matches for conserved functional residues highlighted F. Summary of features shared by LTA4H and potential *D*. *melanogaster* ortholog CG10602.
(PDF)

S5 Fig Sequence and structural details of the modeled fly GGT1 candidate. A. Domain architecture of GGT1 and CG6461 and known/predicted functional residues B. Pairwise alignment of CG6461 and 4GDX generated from structural superposition showing shared secondary structure elements and known/predicted functional residues (marked with red asterisks) C. Pairwise alignment of CG6461 and 4GDX generated from structural superposition with conserved residues highlighted using the physiochemical color scheme (CLUSTALX) D. Validation of the CG6461 model: ProQ2 quality score mapped to a 3D model of CG6461 (left); ProSA global quality score ranking (middle) and per-residue quality graph (right) E. GGT1 (4GDX, cyan-blue) superimposed on the predicted structure of CG6461 (green-red) with potential matches for conserved functional residues highlighted F. Summary of features shared by GGT1 and potential *D*. *melanogaster* ortholog CG6461.
(PDF)

S6 Fig Sequence and structural details of the modeled fly DPEP1 candidate. A. Domain architecture of DPEP1 and CG6154 and known/predicted functional residues B. Pairwise alignment of CG6154 and 1ITQ generated from structural superposition showing shared secondary structure elements and known/predicted functional residues (marked with red asterisks) C. Pairwise alignment of CG6154 and 1ITQ generated from structural superposition with conserved residues highlighted using the physiochemical color scheme (CLUSTALX) D. Validation of the CG6154 model: ProQ2 quality score mapped to a 3D model of CG6154 (left); ProSA global quality score ranking (middle) and per-residue quality graph (right) E. DPEP1 (1ITQ, cyan-blue) superimposed on the predicted structure of CG6154 (green-red) with potential matches for conserved functional residues highlighted F. Summary of features shared by DPEP1 and potential *D*. *melanogaster* ortholog CG6154.
(PDF)

S7 Fig Sequence and structural details of the modeled fly GPX1 candidate. A. Domain architecture of GPX1 and CG12013 and known/predicted functional residues B. Pairwise alignment of CG12013 and 2F8A generated from structural superposition showing shared secondary structure elements and known/predicted functional residues (marked with red asterisk; in the GPX1 crystal structure, the selenocysteine is mutated to a glycine) C. Pairwise alignment of CG12013 and 2F8A generated from structural superposition with conserved residues highlighted using the physiochemical color scheme (CLUSTALX) D. Validation of the CG12013 model: ProQ2 quality score mapped to a 3D model of CG12013 (left); ProSA global quality score ranking (middle) and per-residue quality graph (right) E. GPX1 (2F8A, cyan-blue) superimposed on the predicted structure of CG12013 (green-red) with potential matches for conserved functional residues highlighted F. Summary of features shared by GPX1 and potential *D*. *melanogaster* ortholog CG12013.
(PDF)

S8 Fig Sequence and structural details of the modeled fly CPA1 candidate. A. Domain architecture of CPA1 and CG18585 and known/predicted functional residues B. Pairwise alignment of CG18585 and 3FJU generated from structural superposition showing shared secondary structure elements and known/predicted functional residues (marked with red asterisks) C. Pairwise alignment of CG18585 and 3FJU generated from structural superposition with conserved residues highlighted using the physiochemical color scheme (CLUSTALX) D. Validation of the CG18585 model: ProQ2 quality score mapped to a 3D model of CG18585 (left); ProSA global quality score ranking (middle) and per-residue quality graph (right) E. CPA1 (3FJU, cyan-blue) superimposed on the predicted structure of CG18585 (green-red) with potential matches for conserved functional residues highlighted F. Summary of features shared by CPA1 and potential *D*. *melanogaster* ortholog CG18585.
(PDF)

S9 Fig Sequence and structural details of the modeled fly HPGDS candidate. A. Domain architecture of HPGDS and CG8938 and known/predicted functional residues B. Pairwise alignment of CG8938 and 1IYI generated from structural superposition showing shared secondary structure elements and known/predicted functional residues (marked with red asterisks) C. Pairwise alignment of CG8938 and 1IYI generated from structural superposition with conserved residues highlighted using the physiochemical color scheme (CLUSTALX) D. Validation of the CG8938 model: ProQ2 quality score mapped to a 3D model of CG8938 (left); ProSA global quality score ranking (middle) and per-residue quality graph (right) E. CPA1 (1IYI, cyan-blue) superimposed on the predicted structure of CG8938 (green-red) with potential matches for conserved functional residues highlighted F. Summary of features shared by HPGDS and potential *D*. *melanogaster* ortholog CG8938.
(PDF)

S10 Fig Sequence and structural details of the modeled fly PTGES3 candidate. A. Domain architecture of PTGES3 and CG16817 and known/predicted functional residues B. Pairwise alignment of CG16817 and 1EFJ generated from structural superposition showing shared secondary structure elements C. Pairwise alignment of CG16817 and 1EFJ generated from structural superposition with conserved residues highlighted using the physiochemical color scheme (CLUSTALX) D. Validation of the CG16817 model: ProQ2 quality score mapped to a 3D model of CG16817 (left); ProSA global quality score ranking (middle) and per-residue quality graph (right) E. CPA1 (1EFJ, cyan-blue) superimposed on the predicted structure of CG16817 (green-red) with potential matches for conserved functional residues highlighted F. Summary of features shared by PTGES3 and potential *D*. *melanogaster* ortholog CG16817.
(PDF)

S11 Fig Sequence and structural details of the modeled fly CBR1 candidate. A. Domain architecture of CBR1 and CG11200 and known/predicted functional residues B. Pairwise alignment of CG11200 and 3BHJ generated from structural superposition showing shared secondary structure elements and known/predicted functional residues (marked with red asterisks) C. Pairwise alignment of CG11200 and 3BHJ generated from structural superposition with conserved residues highlighted using the physiochemical color scheme (CLUSTALX) D. Validation of the CG11200 model: ProQ2 quality score mapped to a 3D model of CG11200 (left); ProSA global quality score ranking (middle) and per-residue quality graph (right) E. CPA1 (3BHJ, cyan-blue) superimposed on the predicted structure of CG11200 (green-red) with potential matches for conserved functional residues highlighted F. Summary of features shared by CBR1 and potential *D*. *melanogaster* ortholog CG11200.
(PDF)

S12 Fig Sequence and structural details of the modeled fly HPGD candidate. A. Domain architecture of HPGD and CG18814 and known/predicted functional residues B. Pairwise alignment of CG18814 and 2GDZ generated from structural superposition showing shared secondary structure elements and known/predicted functional residues (marked with red asterisks) C. Pairwise alignment of CG18814 and 2GDZ generated from structural superposition with conserved residues highlighted using the physiochemical color scheme (CLUSTALX) D. Validation of the CG18814 model: ProQ2 quality score mapped to a 3D model of CG18814 (left); ProSA global quality score ranking (middle) and per-residue quality graph (right) E. HPGD (2GDZ, cyan-blue) superimposed on the predicted structure of CG18814 (green-red) with potential matches for conserved functional residues highlighted F. Summary of features shared by HPGD and potential *D*. *melanogaster* ortholog CG18814.
(PDF)

S13 Fig Sequence and structural details of the modeled fly TBXAS1 candidate. A. Domain architecture of TBXAS1 and CG3616 and known/predicted functional residues B. Pairwise alignment of CG3616 and the modeled human TBXAS1 generated from structural superposition showing shared secondary structure elements and known/predicted functional residues (marked with a red asterisk) C. Pairwise alignment of CG3616 and the modeled human TBXAS1 generated from structural superposition with conserved residues highlighted using the physiochemical color scheme (CLUSTALX) D. Validation of the CG3616 model: ProQ2 quality score mapped to a 3D model of CG3616 (left); ProSA global quality score ranking (middle) and per-residue quality graph (right) E. TBXAS1 (model, cyan-blue) superimposed on the predicted structure of CG3616 (green-red) F. Summary of features shared by TBXAS1 and potential *D*. *melanogaster* ortholog CG3616.
(PDF)

S14 Fig Sequence and structural details of the modeled fly PTGS1 candidate. A. Domain architecture of PTGS1 and CG4009 and known/predicted functional residues B. Pairwise alignment of CG4009 and 3N8V generated from structural superposition showing shared secondary structure elements and known/predicted functional residues (marked with red asterisk; the green asterisk marks the putative analog for Y384) C. Pairwise alignment of CG4009 and 3N8V generated from structural superposition with conserved residues highlighted using the physiochemical color scheme (CLUSTALX) D. Validation of the CG4009 model: ProQ2 quality score mapped to a 3D model of CG4009 (left); ProSA global quality score ranking (middle) and per-residue quality graph (right) E. PTGS1 (3N8V, cyan-blue) superimposed on the predicted structure of CG4009 (green-red) with potential matches for conserved functional residues highlighted F. Summary of features shared by PTGS1 and potential *D*. *melanogaster* ortholog CG4009.
(PDF)

S15 Fig Sequence and structural details of the modeled fly PTGDS candidate. A. Domain architecture of PTGDS and CG33126 and known/predicted functional residues B. Pairwise alignment of CG33126 and 5WY9 generated from structural superposition showing shared secondary structure elements and known/predicted functional residues (marked with a red asterisk; the green asterisk denotes the putative analog for C65) C. Pairwise alignment of CG33126 and 5WY9 generated from structural superposition with conserved residues highlighted using the physiochemical color scheme (CLUSTALX) D. Validation of the CG33126 model: ProQ2 quality score mapped to a 3D model of CG33126 (left); ProSA global quality score ranking (middle) and per-residue quality graph (right) E. PTGDS (5WY9, cyan-blue) superimposed on the predicted structure of CG33126 (green-red) with potential matches for conserved functional residues highlighted F. Summary of features shared by PTGDS and potential *D*. *melanogaster* ortholog CG33126.
(PDF)

S16 Fig Sequence and structural details of the modeled fly PTGIS candidate. A. Domain architecture of PTG1S and CG3466 and known/predicted functional residues B. Pairwise alignment of CG34666 and 2IAG generated from structural superposition showing shared secondary structure elements and known/predicted functional residues (marked with a red asterisk) C. Pairwise alignment of CG3466 and 2IAG generated from structural superposition with conserved residues highlighted using the physiochemical color scheme (CLUSTALX) D. Validation of the CG3466 model: ProQ2 quality score mapped to a 3D model of CG3466 (left); ProSA global quality score ranking (middle) and per-residue quality graph (right) E. PTG1S (2IAG, cyan-blue) superimposed on the predicted structure of CG3466 (green-red) with potential matches for conserved functional residues highlighted F. Summary of features shared by PTG1S and potential *D*. *melanogaster* ortholog CG3466.
(PDF)

S17 Fig Sequence and structural details of the modeled fly LTC4S candidate. A. Domain architecture of LTCS4 and CG33178 and known/predicted functional residues B. Pairwise alignment of CG33178 and 2PNO generated from structural superposition showing shared secondary structure elements and known/predicted functional residues (marked with red asterisks; the green asterisk denotes the analog for R31) C. Pairwise alignment of CG33178 and 2PNO generated from structural superposition with conserved residues highlighted using the physiochemical color scheme (CLUSTALX) D. Validation of the CG33178 model: ProQ2 quality score mapped to a 3D model of CG33178 (left); ProSA global quality score ranking (middle) and per-residue quality graph (right) E. LTCS4 (2PNO, cyan-blue) superimposed on the predicted structure of CG33178 (green-red) with potential matches for conserved functional residues highlighted F. Summary of features shared by LTCS4 and potential *D*. *melanogaster* ortholog CG33178.
(PDF)

S18 Fig Sequence and structural details of the modeled fly ALOX5AP candidate. A. Domain architecture of ALOX5AP and CG33177 and known/predicted functional residues B. Pairwise alignment of CG33177 and 2Q7M generated from structural superposition showing shared secondary structure elements C. Pairwise alignment of CG33177 and 2Q7M generated from structural superposition with conserved residues highlighted using the physiochemical color scheme (CLUSTALX) D. Validation of the CG33177 model: ProQ2 quality score mapped to a 3D model of CG33177 (left); ProSA global quality score ranking (middle) and per-residue quality graph (right) E. ALOX5AP (2Q7M, cyan-blue) superimposed on the predicted structure of CG33177 (green-red) F. Summary of features shared by ALOX5AP and potential *D*. *melanogaster* ortholog CG33177.
(PDF)
