# Supplementary material for: A combined computational strategy of sequence and structural analysis predicts the existence of a functional eicosanoid pathway in Drosophila melanogaster
Source: PLoS One. 2019 Feb 12;14(2):e0211897. doi: 10.1371/journal.pone.0211897 (PMC6372189; doi:10.1371/journal.pone.0211897)
Supplement: S1 Fig — A. Domain architecture of PTGES and CG1742 and known/predicted functional residues B. Pairwise alignment of CG1742 and 4AL0 generated from structural superposition showing shared secondary structure elements and known/predicted functional residues (marked with a red asterisk) C. Pairwise alignment of CG1742 and 4AL0 generated from structural superposition with conserved residues highlighted using the physiochemical color scheme (CLUSTALX) D. Validation of the CG1742 model: ProQ2 quality score mapped to a 3D model of CG1742 (left); ProSA global quality score ranking (middle) and per-residue quality graph (right) E. PTGES (4AL0, cyan-blue) superimposed on the predicted structure of CG1742 (green-red) with potential matches for conserved functional residues highlighted F. Summary of features shared by PTGES and potential D. melanogaster ortholog CG1742. (PDF) [file pone.0211897.s001.pdf]

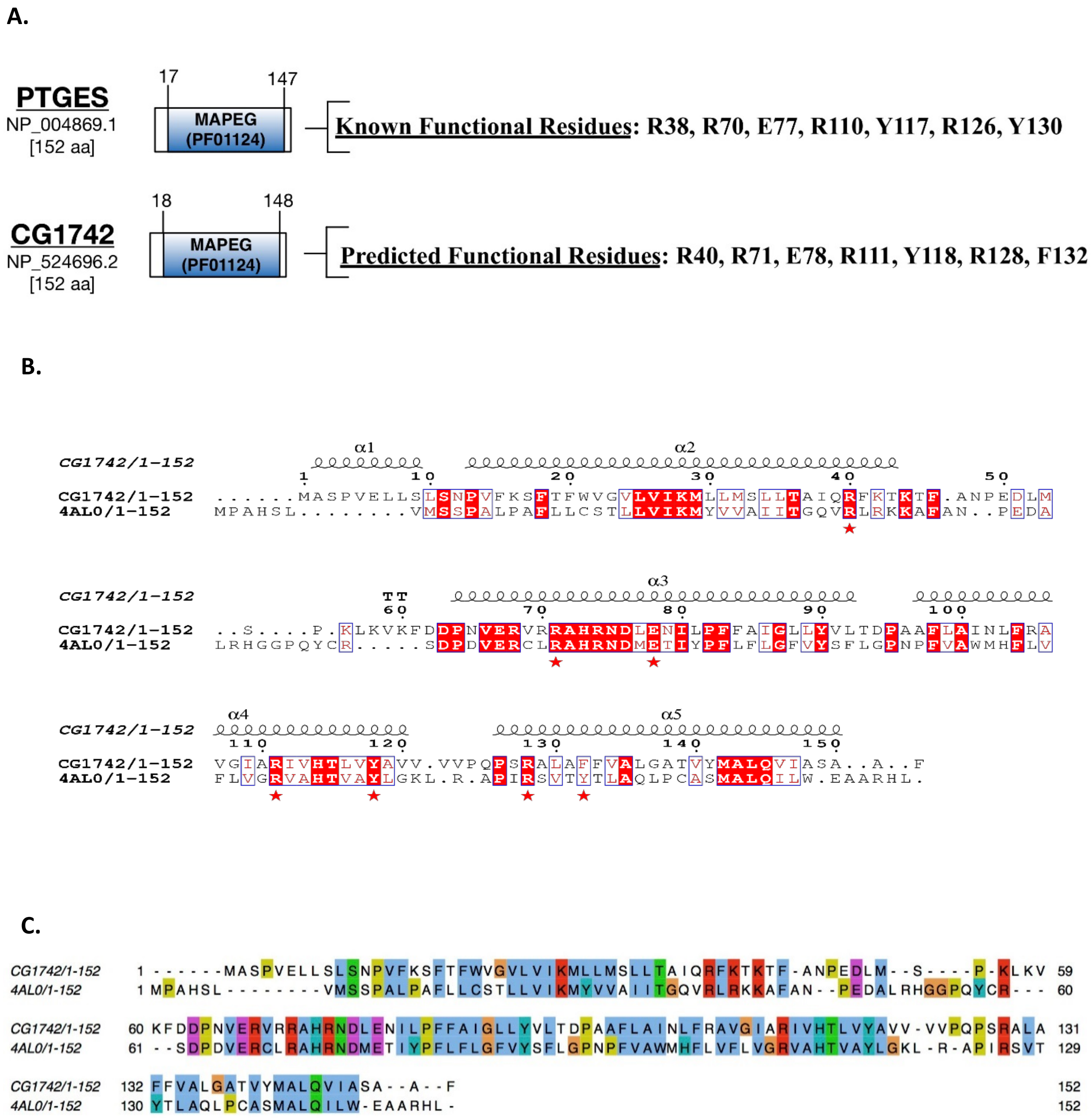

A.
PTGES
NP_004869.1
[152 aa]
17
147
MAPEG
(PF01124)
Known Functional Residues: R38, R70, E77, R110, Y117, R126, Y130
CG1742
NP_524696.2
[152 aa]
18
148
MAPEG
(PF01124)
Predicted Functional Residues: R40, R71, E78, R111, Y118, R128, F132
B.
CG1742/1-152
4AL0/1-152
α1
α2
α3
α4
α5
C.
CG1742/1-152
4AL0/1-152

D.

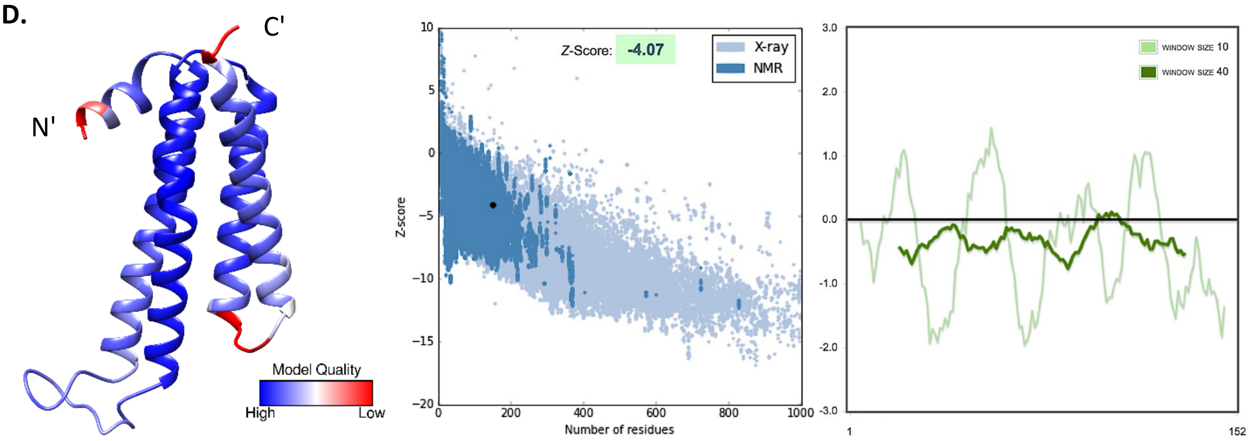

E.

**PTGES Structure**	***D. melanogaster*** **Model**	**Superimposed**
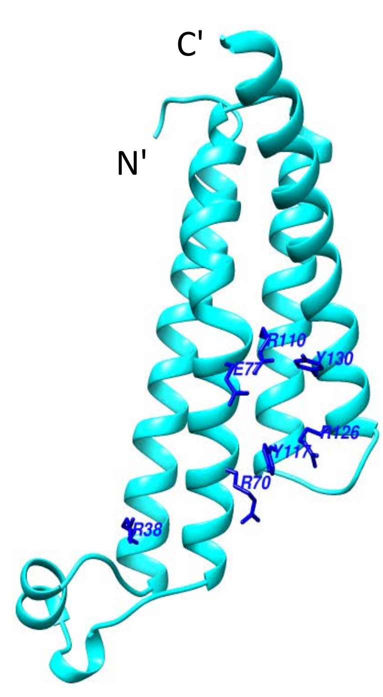	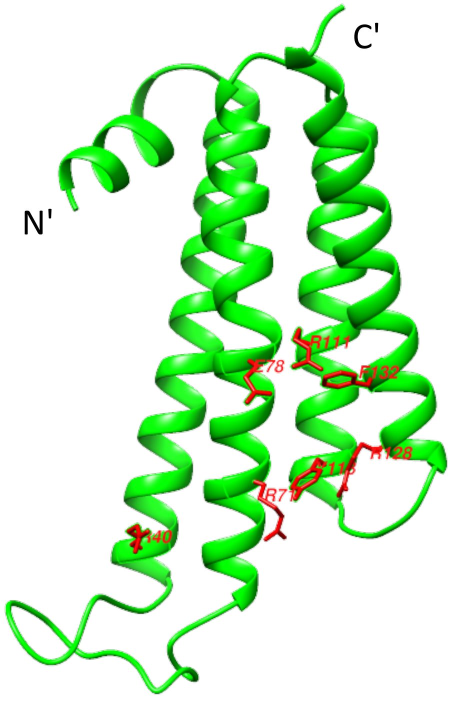	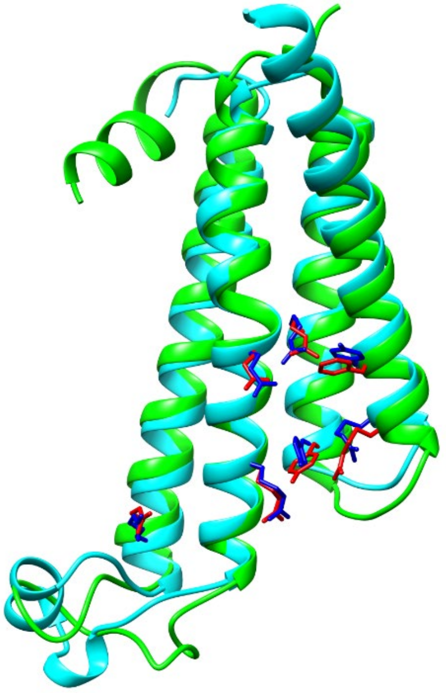

F.

	Length (AA)	Domain Architecture (Pfam, range)	Functional Residues (aligned matches in *D. melanogaster*)	Sequence ID%	Structural Overlap (RMSD)
Prostaglandin-E-synthase (PTGES, NP_004869.1, PDB: 4AL0)	152	MAPEG (PF01124) 16-146	R38, E77, R70, R110, Y117, R126, Y130	34% ID 53% SIM	1.015 Å
MGST-like (CG1742, NP_524696.1)	152	MAPEG (PF01124) 18-148	R40, E78, R71, R111, Y118, R128, F132		

S1 Fig. Sequence and structural details of the modeled fly PTGES candidate. A. Domain architecture of PTGES and CG1742 and known/predicted functional residues B. Pairwise alignment of CG1742 and 4AL0 generated from structural superposition showing shared secondary structure elements and known/predicted functional residues (marked with a red asterisk) C. Pairwise alignment of CG1742 and 4AL0 generated from structural superposition with conserved residues highlighted using the physiochemical color scheme (CLUSTALX) D. Validation of the CG1742 model: ProQ2 quality score mapped to a 3D model of CG1742 (left); ProSA global quality score ranking (middle) and per-residue quality graph (right) E. PTGES (4AL0, cyan-blue) superimposed on the predicted structure of CG1742 (green-red) with potential matches for conserved functional residues highlighted F. Summary of features shared by PTGES and potential *D. melanogaster* ortholog CG1742.
