# Supplementary material for: A combined computational strategy of sequence and structural analysis predicts the existence of a functional eicosanoid pathway in Drosophila melanogaster
Source: PLoS One. 2019 Feb 12;14(2):e0211897. doi: 10.1371/journal.pone.0211897 (PMC6372189; doi:10.1371/journal.pone.0211897)
Supplement: S2 Fig — A. Domain architecture of PTGES2 and CG4086 and known/predicted functional residues B. Pairwise alignment of CG4086 and 1Z9H generated from structural superposition showing shared secondary structure elements and known/predicted functional residues (marked with a red asterisk) C. Pairwise alignment of CG4086 and 1Z9H generated from structural superposition with conserved residues highlighted using the physiochemical color scheme (CLUSTALX) D. Validation of the CG4086 model: ProQ2 quality score mapped to a 3D model of CG4086 (left); ProSA global quality score ranking (middle) and per-residue quality graph (right) E. Truncated PTGES2 (1Z9H, cyan-blue) superimposed on the predicted structure of CG4086 (green-red) with potential matches for conserved functional residues highlighted F. Summary of features shared by PTGES2 and potential D. melanogaster ortholog CG4086. (PDF) [file pone.0211897.s002.pdf]

A.

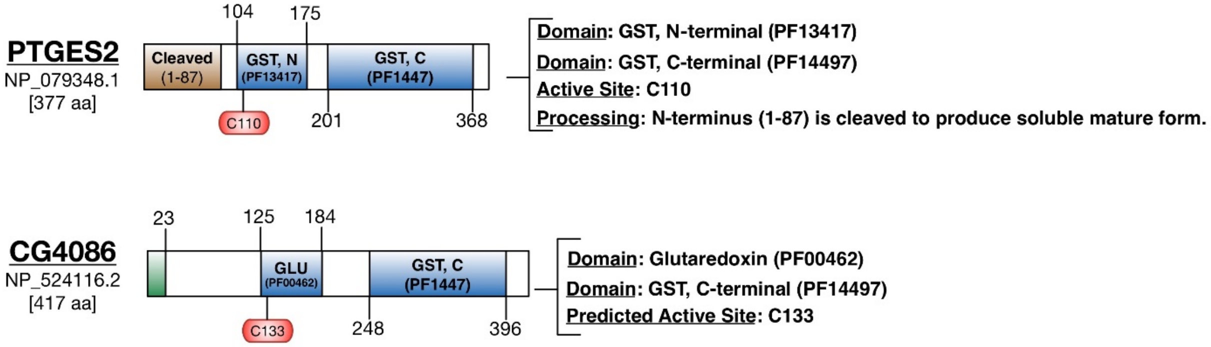
PTGES2
NP_079348.1
[377 aa]
104
175
Cleaved (1-87)
GST, N (PF13417)
GST, C (PF1447)
C110
201
368
Domain: GST, N-terminal (PF13417)
Domain: GST, C-terminal (PF14497)
Active Site: C110
Processing: N-terminus (1-87) is cleaved to produce soluble mature form.
CG4086
NP_524116.2
[417 aa]
23
125
184
GLU (PF00462)
GST, C (PF1447)
C133
248
396
Domain: Glutaredoxin (PF00462)
Domain: GST, C-terminal (PF14497)
Predicted Active Site: C133

B.

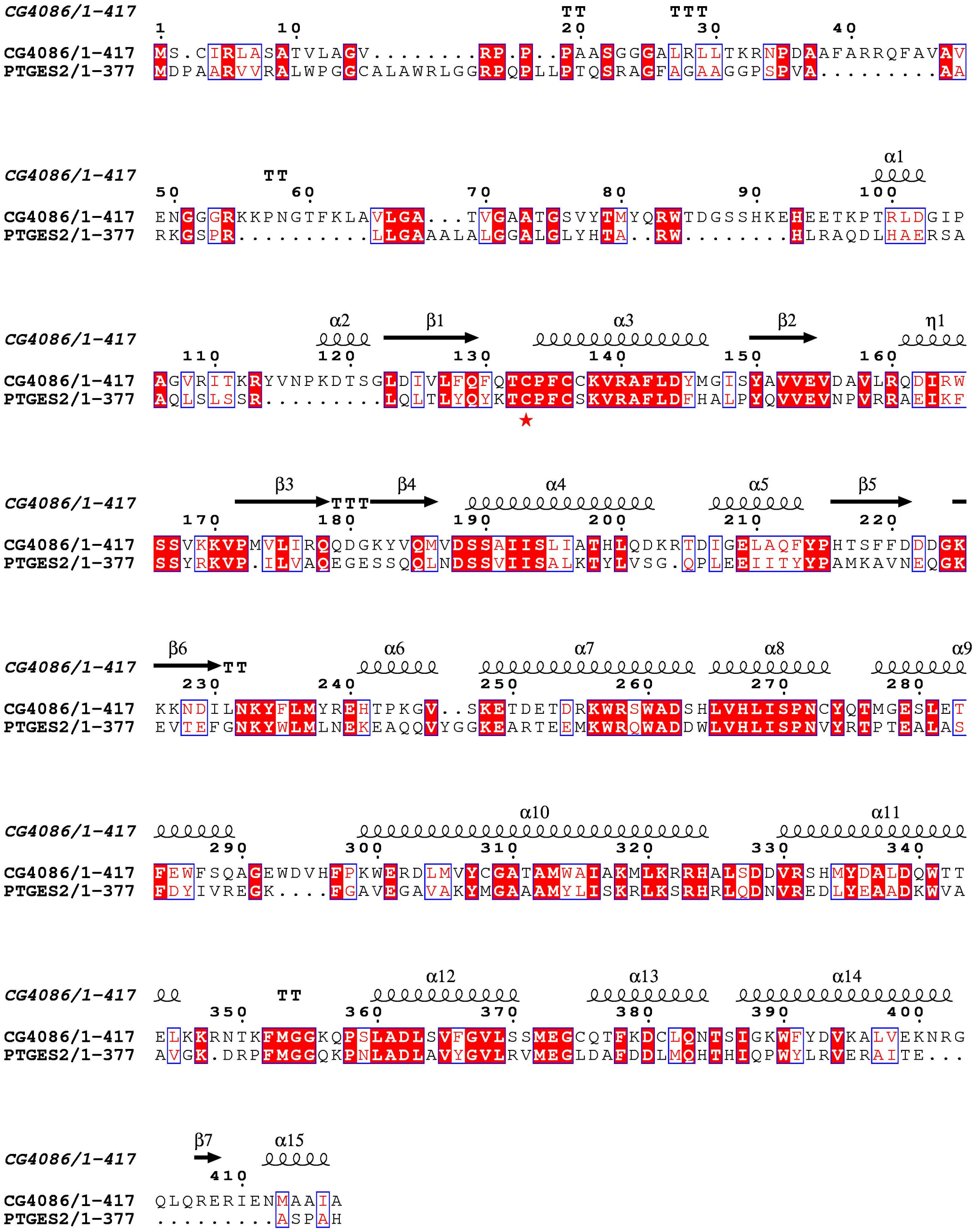
CG4086/1-417
CG4086/1-417 MS.CIRLASATVLAGV........RP.P..PAASGGGALRLLTKRNPDAAFARRQFAVAV
PTGES2/1-377 MDPAARVVRALWPGGCALAWRLGGRPQPLLPTQSRAGFAGAAGGPSPVA.........AA
CG4086/1-417 ENGGGRKKPNGTFKLAVLGA...TVGAATGSVYTMYQRWTDGSSHKEHEETKPTRLDGIP
PTGES2/1-377 RKGSPR..........LLGAAALALGGALGLYHTA..RW........HLRAQDLHAERSA
α1 α2 β1 α3 β2 η1
CG4086/1-417 AGVRITKRYVNPKDTSGLDIVLFQFQTCPFCCKVRAFLDYMGISYAVVEVDAVLRQDIRW
PTGES2/1-377 AQLSLSSR.........LQLTLYQYKTCPFCSKVRAFLDFHALPYQVVEVNPVRRAEIKF
β3 β4 α4 α5 β5
CG4086/1-417 SSVKKVPMVLIRQQDGKYVQMVDSSAIISLIATHLQDKRTDIGELAQFYPHTSFFDDDGK
PTGES2/1-377 SSYRKVP.ILVAQEGESSQQLNDSSVIISALKTYLVSG.QPLEEIITYYPAMKAVNEQGK
β6 α6 α7 α8 α9
CG4086/1-417 KKNDILNKYFLMYREHTPKGV..SKETDETDRKWRSWADSHLVHLISPNCYQTMGESLET
PTGES2/1-377 EVTEFGNKYWLMLNEKEAQQVYGGKEARTEEMKWRQWADDWLVHLISPNVYRTPTEALAS
α10 α11
CG4086/1-417 FEWFSQAGEWDVHFPKWERDLMVYCGATAMWAIAKMLKRRHALSDDVRSHMYDALDQWTT
PTGES2/1-377 FDYIVREGK....FGAVEGAVAKYMGAAAMYLISKRLKSRHRLQDNVREDLYEAADKWVA
α12 α13 α14
CG4086/1-417 ELKKRNTKFMGGKQPSLADLSVFGVLSSMEGCQTFKDCLQNTSIGKWFYDVKALVEKNRG
PTGES2/1-377 AVGK.DRPFMGGQKPNLADLAVYGVLRVMEGLDAFDDLMQHTHIQPWYLRVERAITE...
β7 α15
CG4086/1-417 QLQRERIENMAAIA
PTGES2/1-377 ....ASPAH

C.

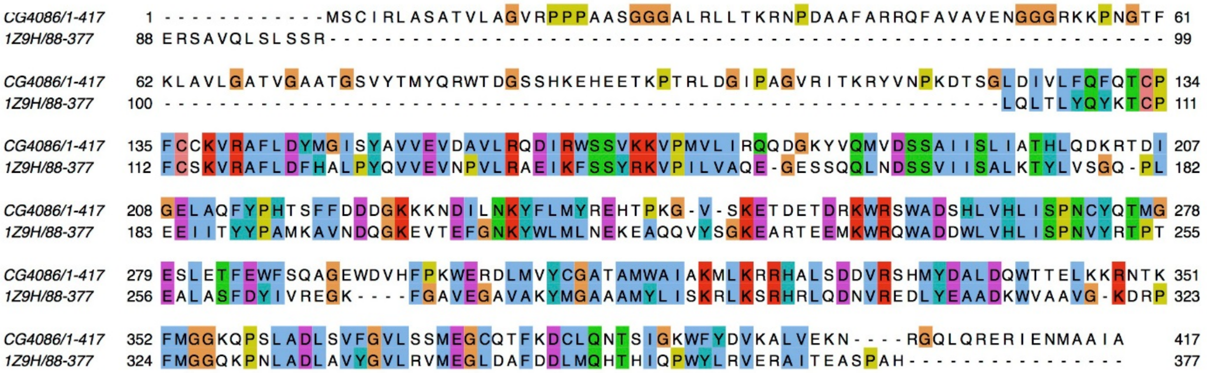

D.

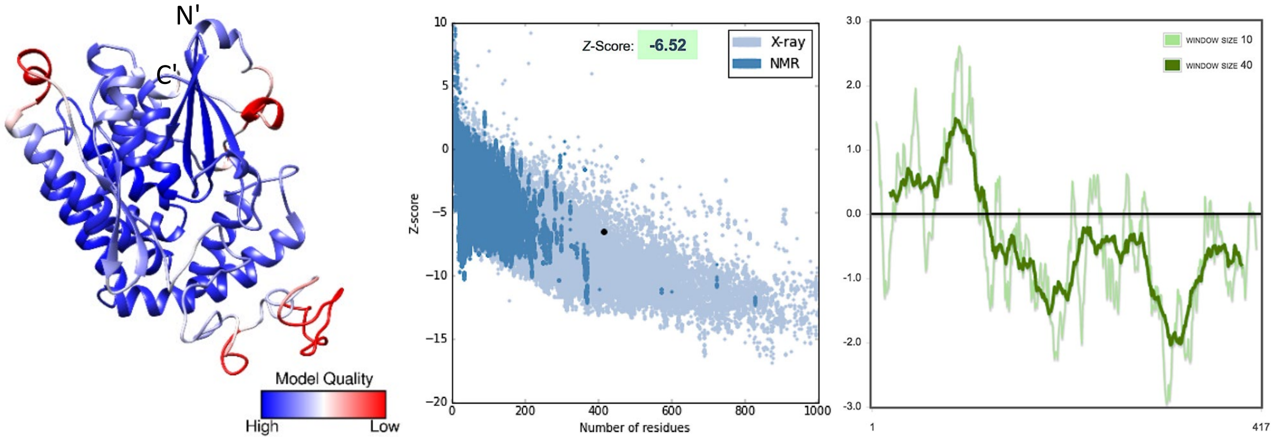

E.

**PTGES Structure**	***D. melanogaster* Model**	**Superimposed**
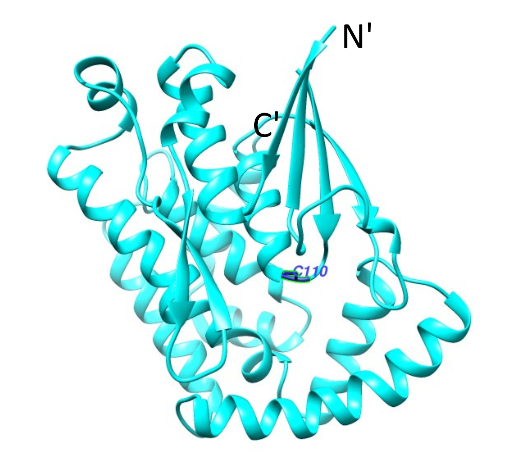	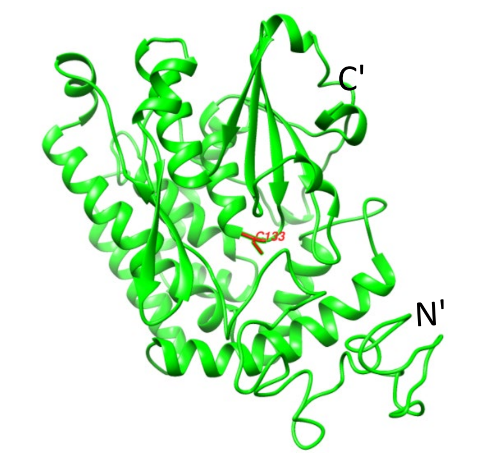	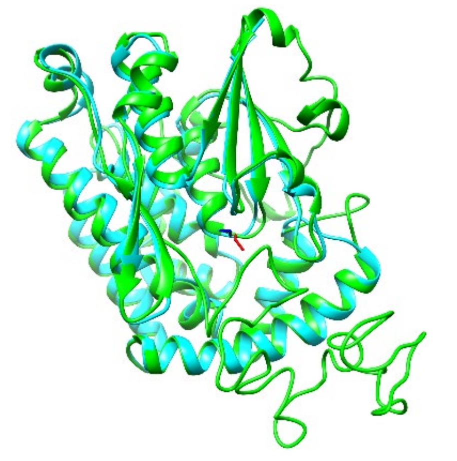

F.

	Length (AA)	Domain Architecture (Pfam, range)	Functional Residues (aligned matches in *D. melanogaster*)	Sequence ID%	Structural Overlap (RMSD)
Prostaglandin-E-synthase 2 (PTGES2, NP_079348.1, PDB: 1Z9H)	377	GST-N3 domain (PF13417) 104-175<br><br>GST-C3 (PF14497) 201-368	C110	33% ID<br>49% SIM	0.814 Å
Suppressor of ref(2)P sterility (CG4086, NP_524116.2)	417	Glutaredoxin domain (PF00462) 125-184<br><br>GST-C domain (PF14497) 248-396	C133		

S2 Fig. Sequence and structural details of the modeled fly PTGES2 candidate. A. Domain architecture of PTGES2 and CG4086 and known/predicted functional residues B. Pairwise alignment of CG4086 and 1Z9H generated from structural superposition showing shared secondary structure elements and known/predicted functional residues (marked with a red asterisk) C. Pairwise alignment of CG4086 and 1Z9H generated from structural superposition with conserved residues highlighted using the physiochemical color scheme (CLUSTALX) D. Validation of the CG4086 model: ProQ2 quality score mapped to a 3D model of CG4086 (left); ProSA global quality score ranking (middle) and per-residue quality graph (right) E. Truncated PTGES2 (1Z9H, cyan-blue) superimposed on the predicted structure of CG4086 (green-red) with potential matches for conserved functional residues highlighted F. Summary of features shared by PTGES2 and potential *D. melanogaster* ortholog CG4086.
