# Supplementary material for: A combined computational strategy of sequence and structural analysis predicts the existence of a functional eicosanoid pathway in Drosophila melanogaster
Source: PLoS One. 2019 Feb 12;14(2):e0211897. doi: 10.1371/journal.pone.0211897 (PMC6372189; doi:10.1371/journal.pone.0211897)
Supplement: S3 Fig — A. Domain architecture of AKR1A1 and CG6084 and known/predicted functional residues B. Pairwise alignment of CG6084 and 2ALR generated from structural superposition showing shared secondary structure elements and known/predicted functional residues (marked with a red asterisk) C. Pairwise alignment of CG6084 and 2ALR generated from structural superposition with conserved residues highlighted using the physiochemical color scheme (CLUSTALX) D. Validation of the CG6084 model: ProQ2 quality score mapped to a 3D model of CG6084 (left); ProSA global quality score ranking (middle) and per-residue quality graph (right) E. AKR1A1 (2ALR, cyan-blue) superimposed on the predicted structure of CG6084 (green-red). RMSD: 0.847 Å with potential matches for conserved functional residues highlighted F. Summary of features shared by Prostacyclin F synthase and potential D. melanogaster ortholog CG6084. (PDF) [file pone.0211897.s003.pdf]

A.

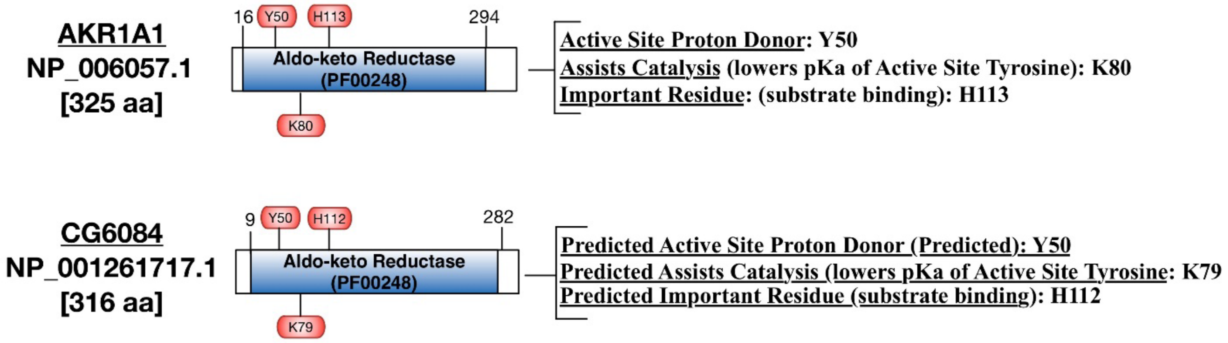
AKR1A1
NP_006057.1
[325 aa]
16
Y50
H113
294
Aldo-keto Reductase
(PF00248)
K80
Active Site Proton Donor: Y50
Assists Catalysis (lowers pKa of Active Site Tyrosine): K80
Important Residue: (substrate binding): H113
CG6084
NP_001261717.1
[316 aa]
9
Y50
H112
282
Aldo-keto Reductase
(PF00248)
K79
Predicted Active Site Proton Donor (Predicted): Y50
Predicted Assists Catalysis (lowers pKa of Active Site Tyrosine: K79
Predicted Important Residue (substrate binding): H112

B.

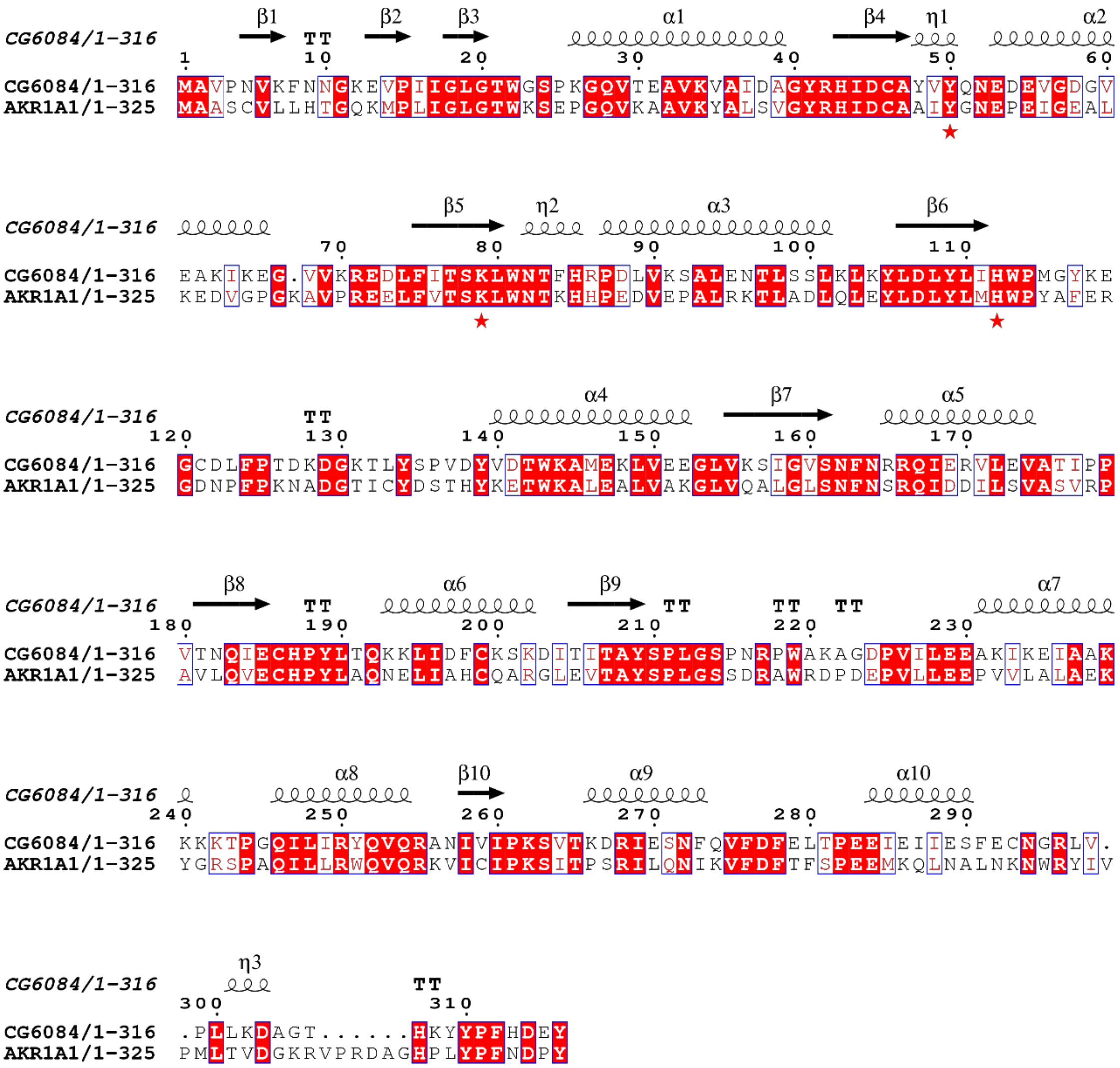
CG6084/1-316 MAVPNVKFNNGKEVPIIGLGTWGSPKGQVTEAVKVAIDAGYRHIDCAYVYQNEDEVGDGV
AKR1A1/1-325 MAASCVLLHTGQKMPLIGLGTWKSEPGQVKAAVKYALSVGYRHIDCAAIYGNEPEIGEAL
CG6084/1-316 EAKIKEG.VVKREDLFITSKLWNTFHRPDLVKSALENTLSSLKLKYLDLYLIHWPMGYKE
AKR1A1/1-325 KEDVGPGKAVPREELFVTSKLWNTKHHPEDVEPALRKTLADLQLEYLDLYLMHWPYAFER
CG6084/1-316 GCDLFPTDKDGKTLYSPVDYVDTWKAMEKLVEEGLVKSIGVSNFNRRQIERVLEVATIPP
AKR1A1/1-325 GDNPFPKNADGTICYDSTHYKETWKALEALVAKGLVQALGLSNFNSRQIDDILSVASVRP
CG6084/1-316 VTNQIECHPYLTQKKLIDFCKSKDITITAYSPLGSPNRPWAKAGDPVILEEAKIKEIAAK
AKR1A1/1-325 AVLQVECHPYLAQNELIAHCQARGLEVTAYSPLGSSDRAWRDPDEPVLLEEPVVLALAEK
CG6084/1-316 KKKTPGQILIRYQVQRANIVIPKSVTKDRIESNFQVFDFELTPEEIEIIESFECNGRLV.
AKR1A1/1-325 YGRSPAQILLRWQVQRKVICIPKSITPSRILQNIKVFDFTFSPEEMKQLNALNKNWRYIV
CG6084/1-316 .PLLKDAGT......HKYYPFHDEY
AKR1A1/1-325 PMLTVDGKRVPRDAGHPLYPFNDPY

C.

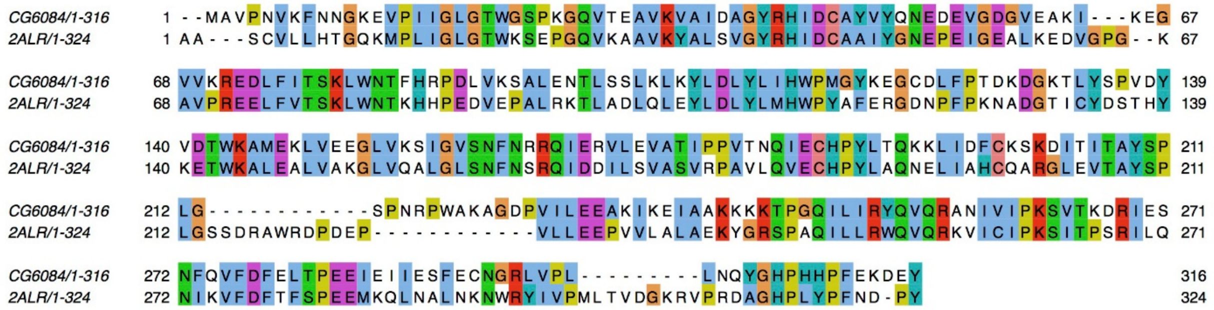

D.

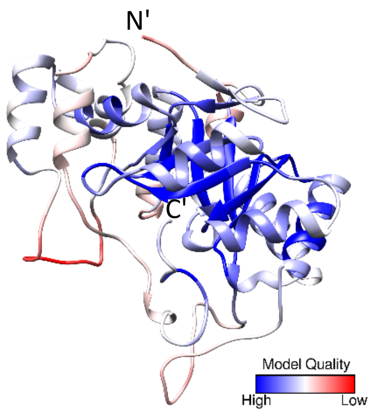

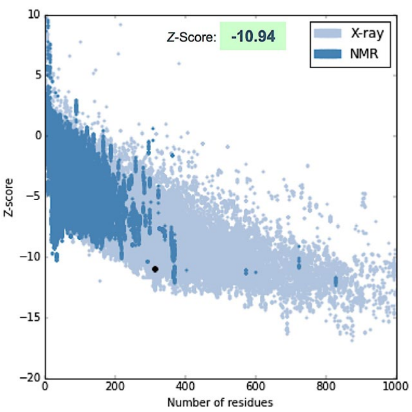

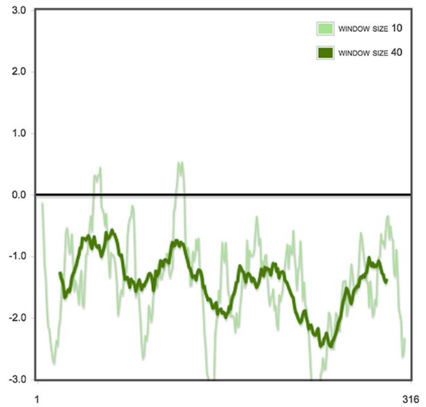

E.

AKR1A1 Structure	*D. melanogaster* Model	Superimposed
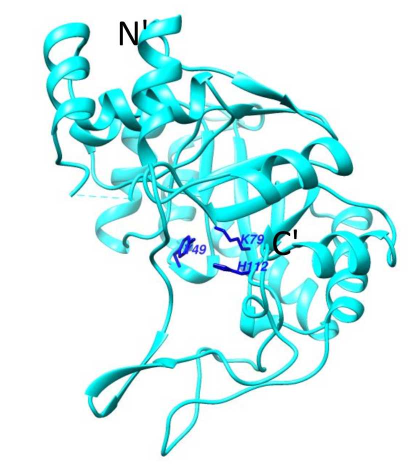	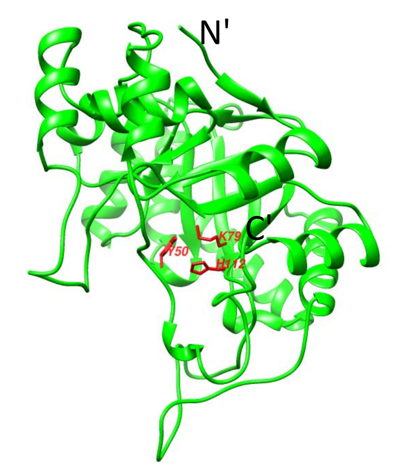	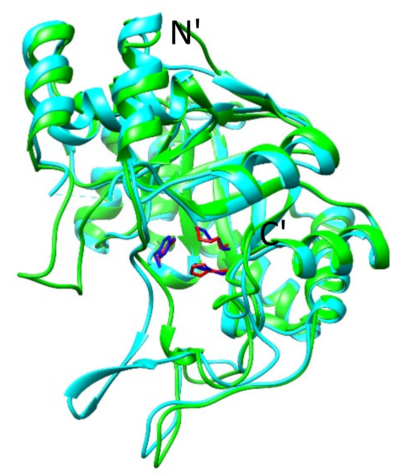

F.	Length (AA)	Domain Architecture (Pfam, range)	Functional Residues (aligned matches in *D. melanogaster*)	Sequence ID%	Structural Overlap (RMSD)
Prostacyclin F synthase (AKR1B1, NP_001619.1)	316	Aldo/keto reductase family (PF00248) 4-289	Y49, K78, H111	48% ID 62% SIM	0.847 Å
Uncharacterized protein (CG6084, NP_648484.1)	316	Aldo/keto reductase family (PF00248) 9-282	Y50, K79, H112		

S3 Fig. Sequence and structural details of the modeled fly Prostacyclin F synthase candidate. A. Domain architecture of AKR1A1 and CG6084 and known/predicted functional residues B. Pairwise alignment of CG6084 and 2ALR generated from structural superposition showing shared secondary structure elements and known/predicted functional residues (marked with a red asterisk) C. Pairwise alignment of CG6084 and 2ALR generated from structural superposition with conserved residues highlighted using the physiochemical color scheme (CLUSTALX) D. Validation of the CG6084 model: ProQ2 quality score mapped to a 3D model of CG6084 (left); ProSA global quality score ranking (middle) and per-residue quality graph (right) E. AKR1A1 (2ALR, cyan-blue) superimposed on the predicted structure of CG6084 (green-red). RMSD: 0.847 Å with potential matches for conserved functional residues highlighted F. Summary of features shared by Prostacyclin F synthase and potential *D. melanogaster* ortholog CG6084.
