# Supplementary material for: A combined computational strategy of sequence and structural analysis predicts the existence of a functional eicosanoid pathway in Drosophila melanogaster
Source: PLoS One. 2019 Feb 12;14(2):e0211897. doi: 10.1371/journal.pone.0211897 (PMC6372189; doi:10.1371/journal.pone.0211897)
Supplement: S4 Fig — A. Domain architecture of LTA4H and CG10602 and known/predicted functional residues B. Pairwise alignment of CG10602 and 3B7U generated from structural superposition showing shared secondary structure elements and known/predicted functional residues (marked with red asterisks) C. Pairwise alignment of CG10602 and 3B7U generated from structural superposition with conserved residues highlighted using the physiochemical color scheme (CLUSTALX) D. Validation of the CG10602 model: ProQ2 quality score mapped to a 3D model of CG10602 (left); ProSA global quality score ranking (middle) and per-residue quality graph (right) E. LTA4H (3B7U, cyan-blue) superimposed on the predicted structure of CG10602 (green-red) with potential matches for conserved functional residues highlighted F. Summary of features shared by LTA4H and potential D. melanogaster ortholog CG10602. (PDF) [file pone.0211897.s004.pdf]

A.

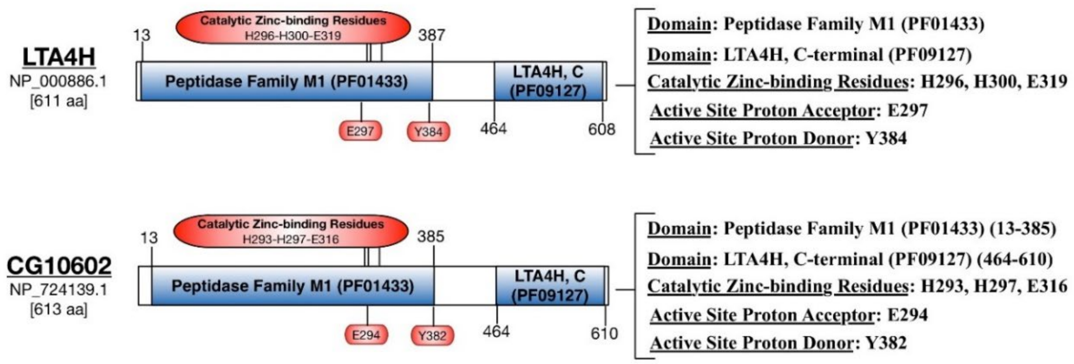
Catalytic Zinc-binding Residues
H296-H300-E319
13
387
LTA4H
NP_000886.1
[611 aa]
Peptidase Family M1 (PF01433)
LTA4H, C (PF09127)
E297
Y384
464
608
Domain: Peptidase Family M1 (PF01433)
Domain: LTA4H, C-terminal (PF09127)
Catalytic Zinc-binding Residues: H296, H300, E319
Active Site Proton Acceptor: E297
Active Site Proton Donor: Y384
Catalytic Zinc-binding Residues
H293-H297-E316
13
385
CG10602
NP_724139.1
[613 aa]
Peptidase Family M1 (PF01433)
LTA4H, C (PF09127)
E294
Y382
464
610
Domain: Peptidase Family M1 (PF01433) (13-385)
Domain: LTA4H, C-terminal (PF09127) (464-610)
Catalytic Zinc-binding Residues: H293, H297, E316
Active Site Proton Acceptor: E294
Active Site Proton Donor: Y382

B.

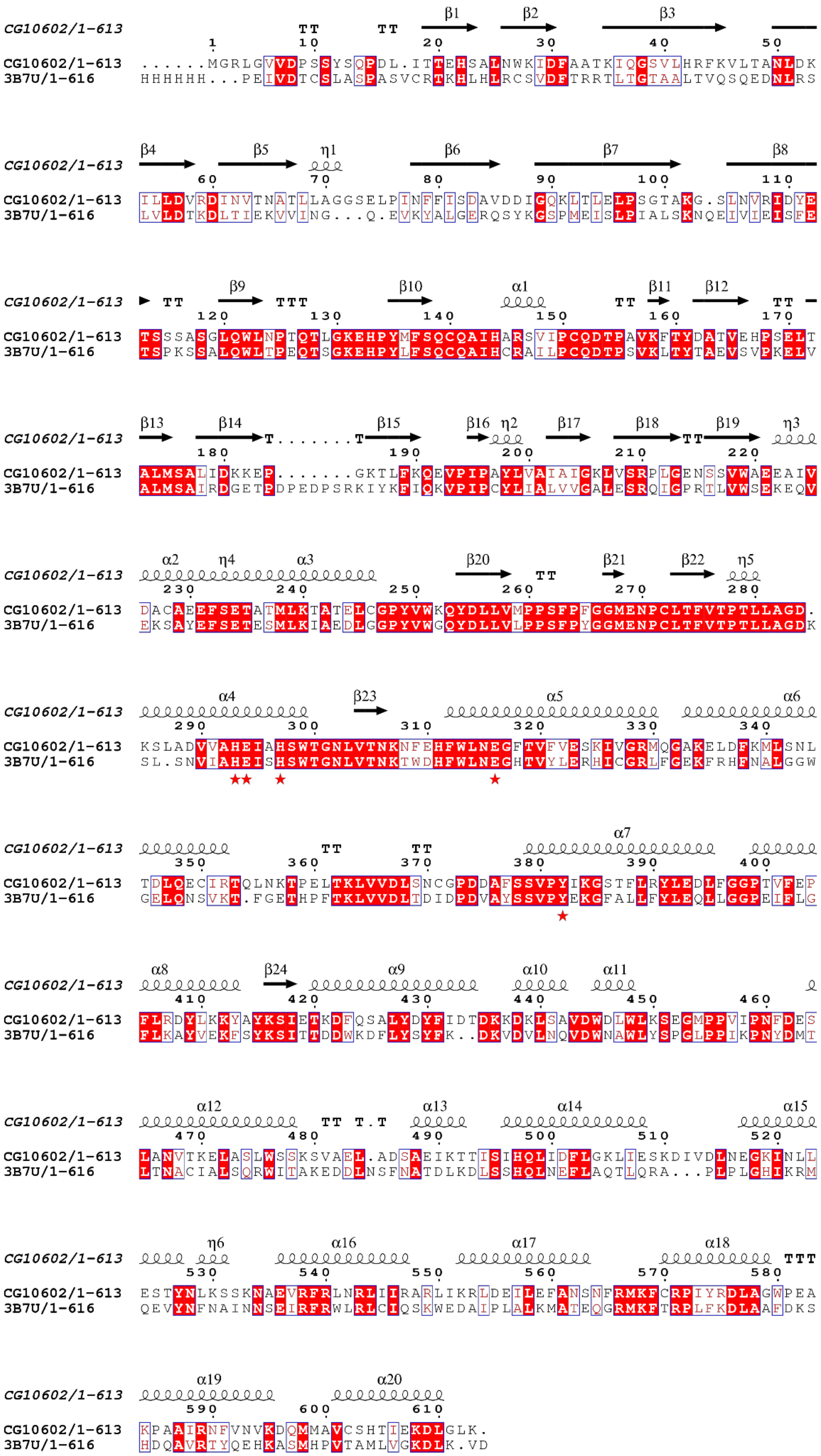
CG10602/1-613 ......MGRLGVVDPSSYSQPDL.ITTEHSALNWKIDFAATKIQGSVLHRFKVLTANLDK
3B7U/1-616 HHHHHH...PEIVDTCSLASPASVCRTKHLHLRCSVDFTRRTLTGTAALTVQSQEDNLRS
CG10602/1-613 ILLDVRDINVTNATLLAGGSELPINFFISDAVDDIGQKLTLELPSGTAKG.SLNVRIDYE
3B7U/1-616 LVLDTKDLTIEKVVING...Q.EVKYALGERQSYKGSPMEISLPIALSKNQEIVIEISFE
CG10602/1-613 TSSSASGLQWLNPTQTLGKEHPYMFSQCQAIHARSVIPCQDTPAVKFTYDATVEHPSELT
3B7U/1-616 TSPKSSALQWLTPEQTSGKEHPYLFSQCQAIHCRAILPCQDTPSVKLTYTAEVSVPKELV
CG10602/1-613 ALMSALIDKKEP.......GKTLFKQEVPIPAYLVAIAIGKLVSRPLGENSSVWAEEAIV
3B7U/1-616 ALMSAIRDGETPDPEDPSRKIYKFIQKVPIPCYLIALVVGALESRQIGPRTLVWSEKEQV
CG10602/1-613 DACAEEFSETATMLKTATELCGPYVWKQYDLLVMPPSFPFGGMENPCLTFVTPTLLAGD.
3B7U/1-616 EKSAYEFSETESMLKIAEDLGGPYVWGQYDLLVLPPSFPYGGMENPCLTFVTPTLLAGDK
CG10602/1-613 KSLADVVAHEIAHSWTGNLVTNKNFEHFWLNEGFTVFVESKIVGRMQGAKELDFKMLSNL
3B7U/1-616 SL.SNVIAHEISHSWTGNLVTNKTWDHFWLNEGHTVYLERHICGRLFGEKFRHFNALGGW
CG10602/1-613 TDLQECIRTQLNKTPELTKLVVDLSNCGPDDAFSSVPYIKGSTFLRYLEDLFGGPTVFEP
3B7U/1-616 GELQNSVKT.FGETHPFTKLVVDLTDIDPDVAYSSVPYEKGFALLFYLEQLLGGPEIFLG
CG10602/1-613 FLRDYLKKYAYKSIETKDFQSALYDYFIDTDKKDKLSAVDWDLWLKSEGMPPVIPNFDES
3B7U/1-616 FLKAYVEKFSYKSITTDDWKDFLYSYFK..DKVDVLNQVDWNAWLYSPGLPPIKPNYDMT
CG10602/1-613 LANVTKELASLWSSKSVAEL.ADSAEIKTTISIHQLIDFLGKLIESKDIVDLNEGKINLL
3B7U/1-616 LTNACIALSQRWITAKEDDLNSFNATDLKDLSSHQLNEFLAQTLQRA...PLPLGHIKRM
CG10602/1-613 ESTYNLKSSKNAEVRFRLNRLIIRARLIKRLDEILEFANSNFRMKFCRPIYRDLAGWPEA
3B7U/1-616 QEVYNFNAINNSEIRFRWLRLCIQSKWEDAIPLALKMATEQGRMKFTRPLFKDLAAFDKS
CG10602/1-613 KPAAIRNFVNVKDQMMAVCSHTIEKDLGLK.
3B7U/1-616 HDQAVRTYQEHKASMHPVTAMLVGKDLK.VD

C.

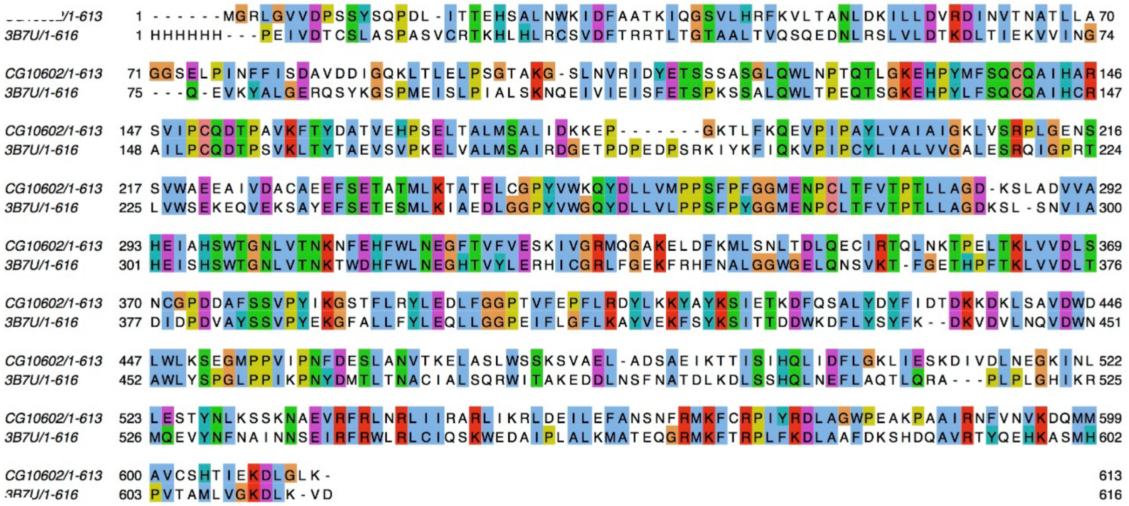

D.

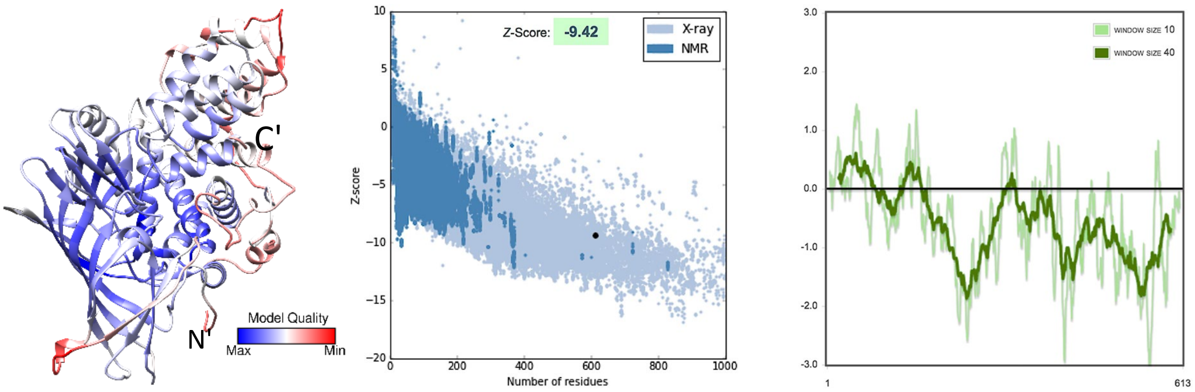

E.

LTA4H Structure	*D. melanogaster* Model	Superimposed
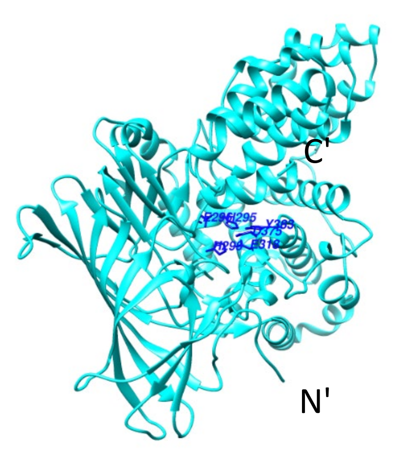	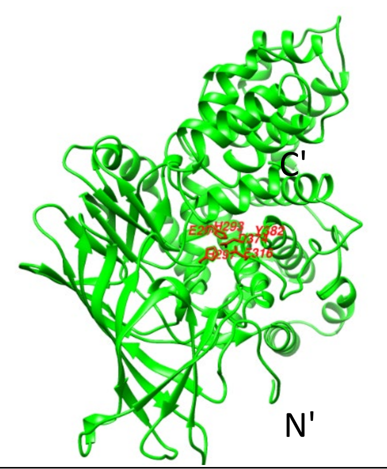	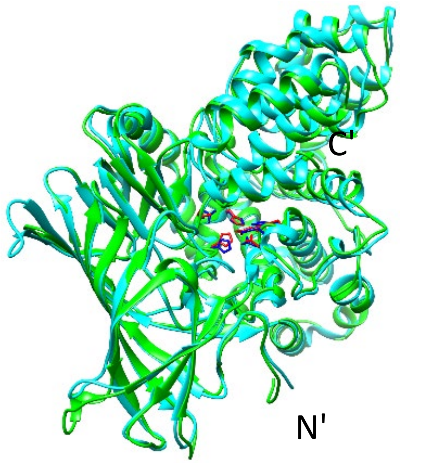

<table>
<tr><th>F.</th><th>Length (AA)</th><th>Domain Architecture (Pfam, range)</th><th>Functional Residues (aligned matches in D. melanogaster)</th><th>Sequence ID%</th><th>Structural Overlap (RMSD)</th></tr>
<tr><td>Leukotriene A4 hydrolase (LTA4H, NP_000886.1, PDB: 3B7U)</td><td>611</td><td>Peptidase family M1 (PF01433) 13-387

LTA4H, C-term (PF09127) 464-608</td><td>H296, E297, H300, E319, D376 and Y384</td><td rowspan="2">58% SIM</td><td rowspan="2">0.891 Å</td></tr>
<tr><td>Uncharacterized protein (CG10602, NP_724139.1)</td><td>613</td><td>Peptidase family M1 (PF01433) 8-420

LTA4H, C-term (PF09127) 464-610</td><td>H293, E294, H297, E316, D374 and Y382</td></tr>
</table>

S4 Fig. Sequence and structural details of the modeled fly LTA4H candidate. A. Domain architecture of LTA4H and CG10602 and known/predicted functional residues B. Pairwise alignment of CG10602 and 3B7U generated from structural superposition showing shared secondary structure elements and known/predicted functional residues (marked with red asterisks) C. Pairwise alignment of CG10602 and 3B7U generated from structural superposition with conserved residues highlighted using the physiochemical color scheme (CLUSTALX) D. Validation of the CG10602 model: ProQ2 quality score mapped to a 3D model of CG10602 (left); ProSA global quality score ranking (middle) and per-residue quality graph (right) E. LTA4H (3B7U, cyan-blue) superimposed on the predicted structure of CG10602 (green-red) with potential matches for conserved functional residues highlighted F. Summary of features shared by LTA4H and potential *D. melanogaster* ortholog CG10602.
