# Supplementary material for: A combined computational strategy of sequence and structural analysis predicts the existence of a functional eicosanoid pathway in Drosophila melanogaster
Source: PLoS One. 2019 Feb 12;14(2):e0211897. doi: 10.1371/journal.pone.0211897 (PMC6372189; doi:10.1371/journal.pone.0211897)
Supplement: S5 Fig — A. Domain architecture of GGT1 and CG6461 and known/predicted functional residues B. Pairwise alignment of CG6461 and 4GDX generated from structural superposition showing shared secondary structure elements and known/predicted functional residues (marked with red asterisks) C. Pairwise alignment of CG6461 and 4GDX generated from structural superposition with conserved residues highlighted using the physiochemical color scheme (CLUSTALX) D. Validation of the CG6461 model: ProQ2 quality score mapped to a 3D model of CG6461 (left); ProSA global quality score ranking (middle) and per-residue quality graph (right) E. GGT1 (4GDX, cyan-blue) superimposed on the predicted structure of CG6461 (green-red) with potential matches for conserved functional residues highlighted F. Summary of features shared by GGT1 and potential D. melanogaster ortholog CG6461. (PDF) [file pone.0211897.s005.pdf]

A.

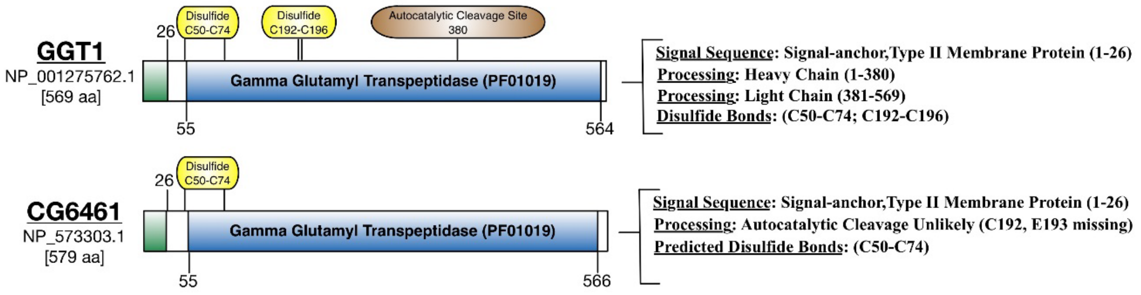
Disulfide C50-C74
Disulfide C192-C196
Autocatalytic Cleavage Site 380
26
GGT1
NP_001275762.1
[569 aa]
Gamma Glutamyl Transpeptidase (PF01019)
55
564
Signal Sequence: Signal-anchor,Type II Membrane Protein (1-26)
Processing: Heavy Chain (1-380)
Processing: Light Chain (381-569)
Disulfide Bonds: (C50-C74; C192-C196)
Disulfide C50-C74
26
CG6461
NP_573303.1
[579 aa]
Gamma Glutamyl Transpeptidase (PF01019)
55
566
Signal Sequence: Signal-anchor,Type II Membrane Protein (1-26)
Processing: Autocatalytic Cleavage Unlikely (C192, E193 missing)
Predicted Disulfide Bonds: (C50-C74)

B.

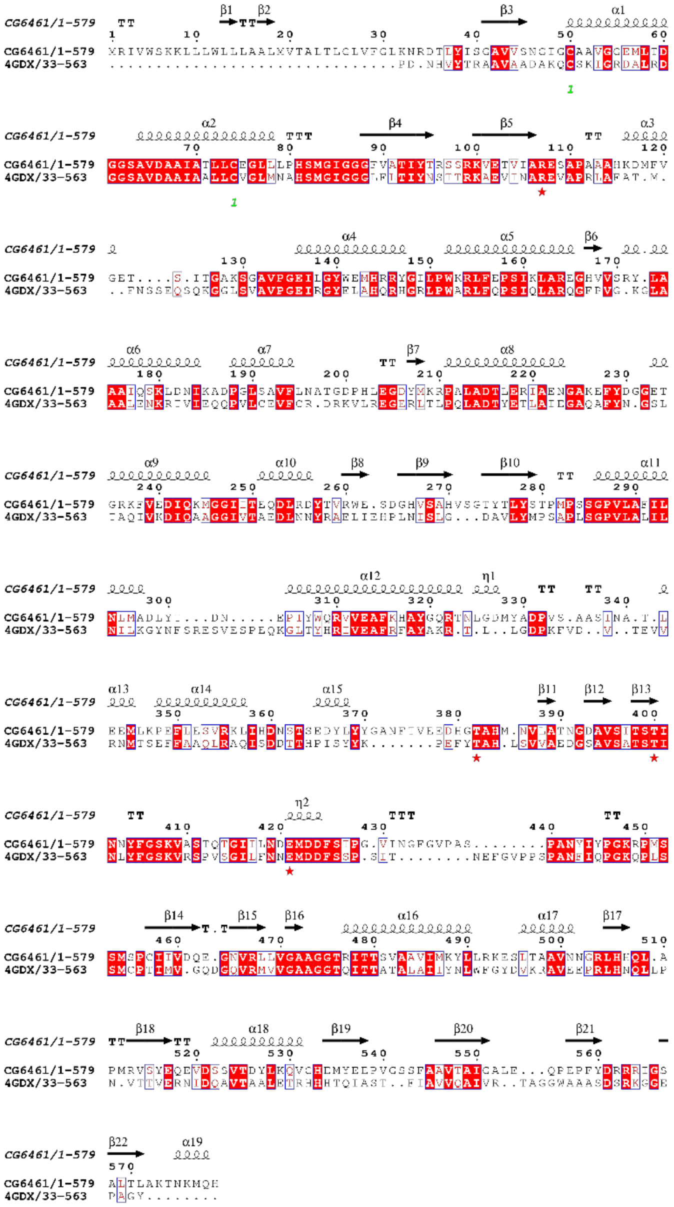
CG6461/1-579
4GDX/33-563

C.

```
1-579          1 MRIVWSKKLLLWLLLAALMVTALTLGLVFGLKNRDTLYISGAVVS 45
4GDX/33-563   33 -------------------------------PD-NHVYTRAAVAA 45

CG6461/1-579  46 NGIGCAAVGGEMLTDGGSAVDAAIATLLCEGLLLPHSMGIGGGFV 90
4GDX/33-563   46 DAKQCSKIGRDALRDGGSAVDAAIAALLCVGLMNAHSMGIGGGLF 90

CG6461/1-579  91 ATIYTRSSRKVETVIARESAPAAAHKDMFVGET----S-ITGAKS 130
4GDX/33-563   91 LTIYNSTTRKAEVINAREVAPRLAFAT-M---FNSSEQSQKGGLS 131

CG6461/1-579 131 GAVPGEILGYWEMHRRYGILPWKRLFEPSIKLAREGHVVSRY-LA 174
4GDX/33-563  132 VAVPGEIRGYELAHQRHGRLPWARLFQPSIQLARQGFPVG-KGLA 175

CG6461/1-579 175 AAIQSKLDNIKADPGLSAVFLNATGDPHLEGDYMKRPALADTLER 219
4GDX/33-563  176 AALENKRTVIEQQPVLCEVFCR-DRKVLREGERLTLPQLADTYET 219

CG6461/1-579 220 IAENGAKEFYDGGETGRKFVEDIQKMGGIITEQDLRDYTVRWE-S 263
4GDX/33-563  220 LAIEGAQAFYN-GSLTAQIVKDIQAAGGIVTAEDLNNYRAELIEH 263

CG6461/1-579 264 DGHVSAHVSGTYTLYSTPMPSSGPVLAFILNLMADLYT---DN-- 303
4GDX/33-563  264 PLNISLG---DAVLYMPSAPLSGPVLALILNILKGYNFSRESVES 305

CG6461/1-579 304 ---EPIYWQRVVEAFKHAYGQRTNLGDMYADPVS-AASINA-T-L 342
4GDX/33-563  306 PEQKGLTYHRIVEAFRFAYAKR-T-L--LGDPKFVD--V--TEVV 342

CG6461/1-579 343 EEMLKPEFLESVRKLIHDNSTSEDYLYYGANFTVEEDHGTA---- 383
4GDX/33-563  343 RNMTSEFFAAQLRAQISDDTTHPISYYK--P----------EFYT 375

CG6461/1-579 384 -HM-NVLATNGDAVSITSTINNYFGSKVASTQTGIILNDEMDDFS 426
4GDX/33-563  376 AH-LSVVAEDGSAVSATSTINLYFGSKVRSPVSGILFNNEMDDFS 419

CG6461/1-579 427 TPG-VINGFGVPAS--------PANYIYPGKRPMSSMSPCIIVDQ 462
4GDX/33-563  420 SP-SIT---------NEFGVPPSPANFIQPGKQPLSSMCPTIMV-G 454

CG6461/1-579 463 E-GNVRLLVGAAGGTRITTSVAAVIMKYLLRKESLTAAVNNGRLH 506
4GDX/33-563  455 QDGQVRMVVGAAGGTQITTATALAIIYNLWFGYDVKRAVEEPRLH 499

CG6461/1-579 507 HQL-APMRVSYEQEVDSSVTDYLKQVGHEMYEEPVGSSFAAVTAI 550
4GDX/33-563  500 NQLLPN-VTTVERNIDQAVTAALETRHHHTQIAST--FIAVVQAI 541

CG6461/1-579 551 GALE---QPEPFYDRRRIGSALTLAKTNKMQH             579
4GDX/33-563  542 VR--TAGGWAAASDSRKGGEPAGY--------             563
```

D.

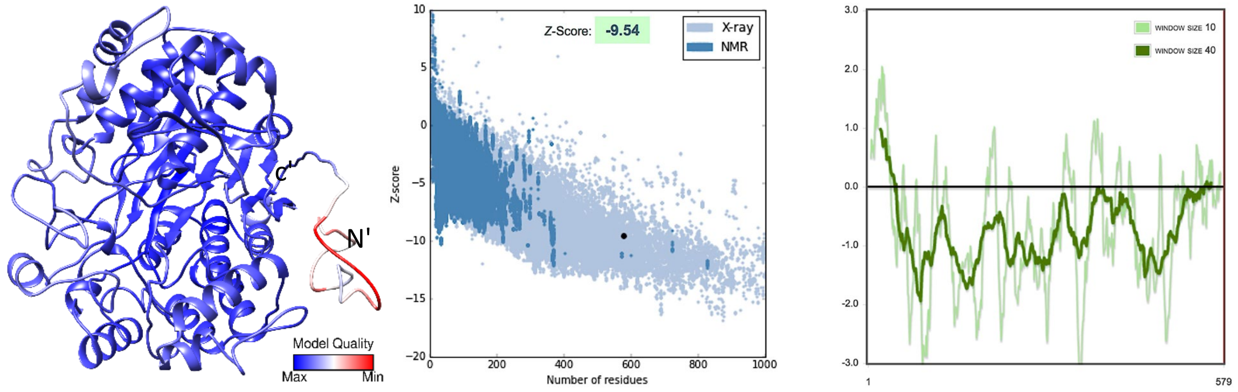

E.

<table>
<tr><th>GGT1 Structure</th><th>D. melanogaster Model</th><th>Superimposed</th></tr>
<tr><td>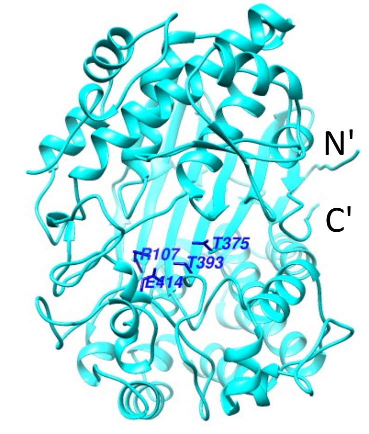
</td><td>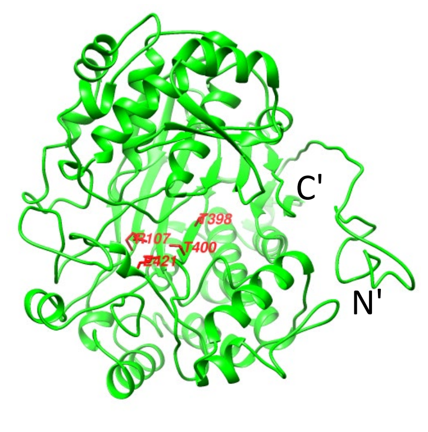
</td><td>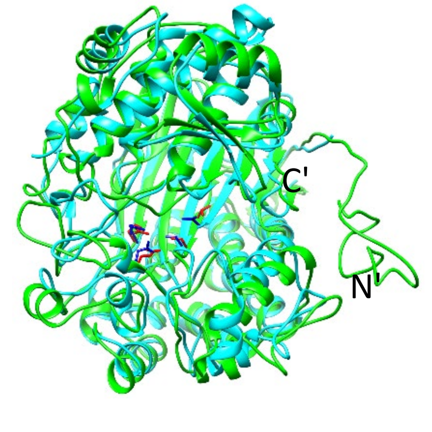
</td></tr>
</table>

F.	Length (AA)	Domain Architecture (Pfam, range)	Functional Residues (aligned matches in *D. melanogaster*)	Sequence ID%	Structural Overlap (RMSD)
Gamma-glutamyltranspeptidase 1 (GGT1, NP_001275762.1, PDB: 4GDX)	569	Gamma-glutamyltranspeptidase (PF01019) 55-564	T381	39% ID 54% SIM	1.126 Å
Gamma-glutamyltranspeptidase (CG6461, NP_573303.1)	579	Gamma-glutamyltranspeptidase (PF01019) 56-566	T382		

S5 Fig. Sequence and structural details of the modeled fly GGT1 candidate. A. Domain architecture of GGT1 and CG6461 and known/predicted functional residues B. Pairwise alignment of CG6461 and 4GDX generated from structural superposition showing shared secondary structure elements and known/predicted functional residues (marked with red asterisks) C. Pairwise alignment of CG6461 and 4GDX generated from structural superposition with conserved residues highlighted using the physiochemical color scheme (CLUSTALX) D. Validation of the CG6461 model: ProQ2 quality score mapped to a 3D model of CG6461 (left); ProSA global quality score ranking (middle) and per-residue quality graph (right) E. GGT1 (4GDX, cyan-blue) superimposed on the predicted structure of CG6461 (green-red) with potential matches for conserved functional residues highlighted F. Summary of features shared by GGT1 and potential *D. melanogaster* ortholog CG6461.

.
