# Supplementary material for: A combined computational strategy of sequence and structural analysis predicts the existence of a functional eicosanoid pathway in Drosophila melanogaster
Source: PLoS One. 2019 Feb 12;14(2):e0211897. doi: 10.1371/journal.pone.0211897 (PMC6372189; doi:10.1371/journal.pone.0211897)
Supplement: S6 Fig — A. Domain architecture of DPEP1 and CG6154 and known/predicted functional residues B. Pairwise alignment of CG6154 and 1ITQ generated from structural superposition showing shared secondary structure elements and known/predicted functional residues (marked with red asterisks) C. Pairwise alignment of CG6154 and 1ITQ generated from structural superposition with conserved residues highlighted using the physiochemical color scheme (CLUSTALX) D. Validation of the CG6154 model: ProQ2 quality score mapped to a 3D model of CG6154 (left); ProSA global quality score ranking (middle) and per-residue quality graph (right) E. DPEP1 (1ITQ, cyan-blue) superimposed on the predicted structure of CG6154 (green-red) with potential matches for conserved functional residues highlighted F. Summary of features shared by DPEP1 and potential D. melanogaster ortholog CG6154. (PDF) [file pone.0211897.s006.pdf]

A.

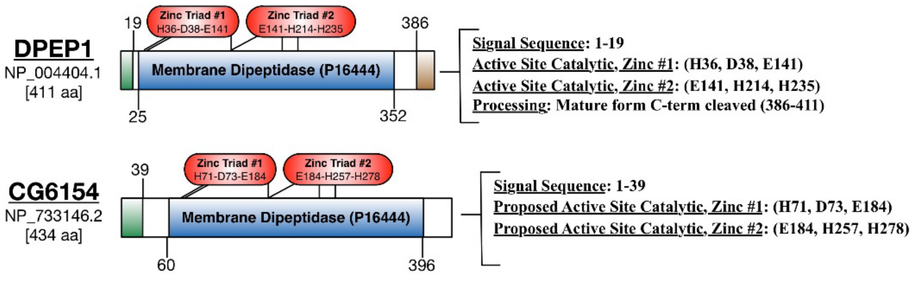
DPEP1
NP_004404.1
[411 aa]
19
25
386
352
Zinc Triad #1
H36-D38-E141
Zinc Triad #2
E141-H214-H235
Membrane Dipeptidase (P16444)
Signal Sequence: 1-19
Active Site Catalytic, Zinc #1: (H36, D38, E141)
Active Site Catalytic, Zinc #2: (E141, H214, H235)
Processing: Mature form C-term cleaved (386-411)
CG6154
NP_733146.2
[434 aa]
39
60
396
Zinc Triad #1
H71-D73-E184
Zinc Triad #2
E184-H257-H278
Membrane Dipeptidase (P16444)
Signal Sequence: 1-39
Proposed Active Site Catalytic, Zinc #1: (H71, D73, E184)
Proposed Active Site Catalytic, Zinc #2: (E184, H257, H278)

B.

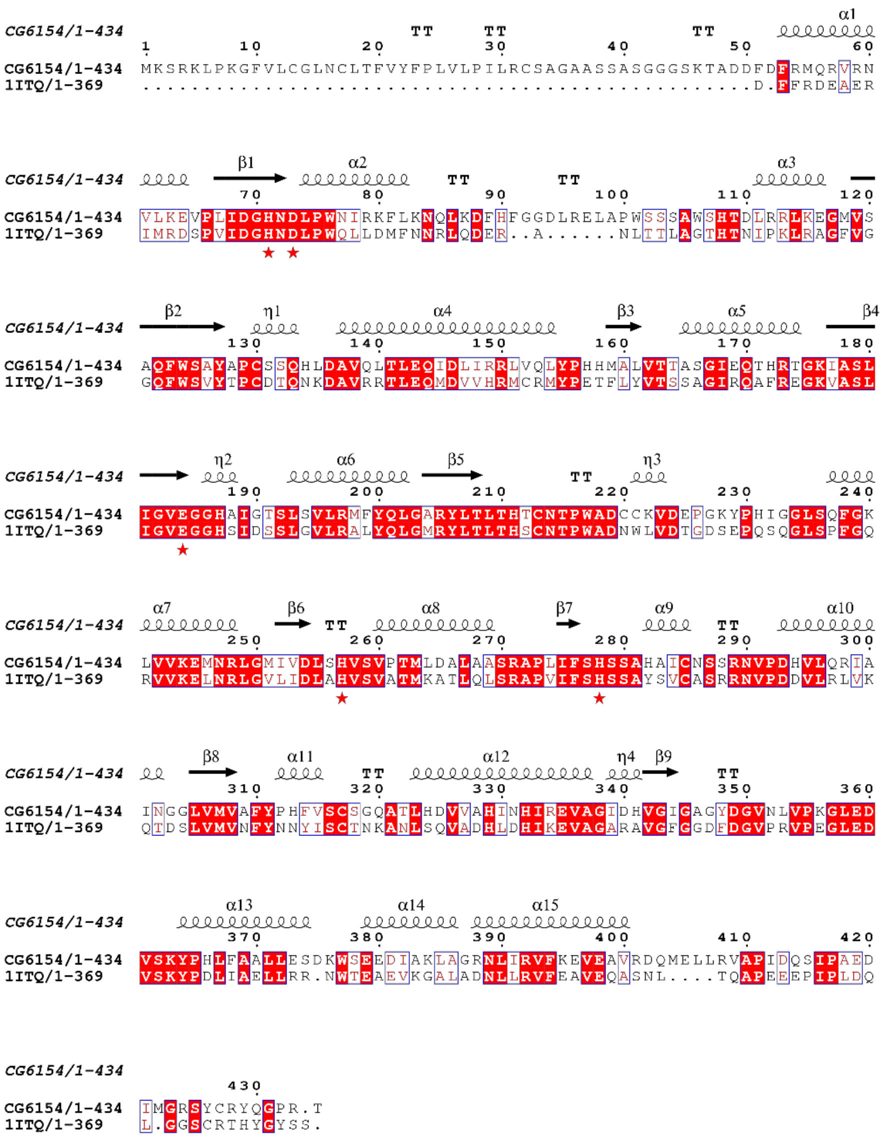
CG6154/1-434 MKSRKLPKGFVLCGLNCLTFVYFPLVLPILRCSAGAASSASGGGSKTADDFDFRMQRVRN
1ITQ/1-369 ....D.FFRDEAER
CG6154/1-434 VLKEVPLIDGHNDLPWNIRKFLKNQLKDFHFGGDLRELAPWSSSAWSHTDLRRLKEGMVS
1ITQ/1-369 IMRDSPVIDGHNDLPWQLLDMFNNRLQDER..A......NLTTLAGTHTNIPKKLRAGFVG
CG6154/1-434 AQFWSAYAPCSSQHLDAVQLTLEQIDLIRRLVQLYPHHMALVTTASGIEQTHRTGKIASL
1ITQ/1-369 GQFWSVYTPCDTQNKDAVRRTLEQMDVVHRMCRMYPETFLYVTSSAGIRQAFREGKVASL
CG6154/1-434 IGVEGGHAIGTSLSVLRMFYQLGARYLTLTHTCNTPWADCCKVDEPGKYPHIGGLSQFGK
1ITQ/1-369 IGVEGGHSIDSSLGVLRALYQLGMRYLTLTHSCNTPWADNWLVDTGDSEPQSQGLSPFGQ
CG6154/1-434 LVVKEMNRLGMIVDLSHVSVPTMLDALAASRAPLIFSHSSAHAICNSSRNVPDHVLQRIA
1ITQ/1-369 RVVKELNRLGVLIDLAHVSVATMKATLQLSRAPVIFSHSSAYSVCASRRNVPDDVLRLVK
CG6154/1-434 INGGLVMVAFYPHFVSCSGQATLHDVVAHINHIREVAGIDHVGIGAGYDGVNLVPKGLED
1ITQ/1-369 QTDSLVMVNFYNNYISCTNKANLSQVADHLDHIKEVAGARAVGFGGDFDGVPRVPEGLED
CG6154/1-434 VSKYPHLFAALLESDKWSEEDIAKLAGRNLIRVFKEVEAVRDQMELLRVAPIDQSIPAED
1ITQ/1-369 VSKYPDLIAELLRR.NWTEAEVKGALADNLLRVFEAVEQASNL....TQAPEEEPIPLDQ
CG6154/1-434 IMGRSYCRYQGPR.T
1ITQ/1-369 L.GGSCRTHYGYSS.

C.

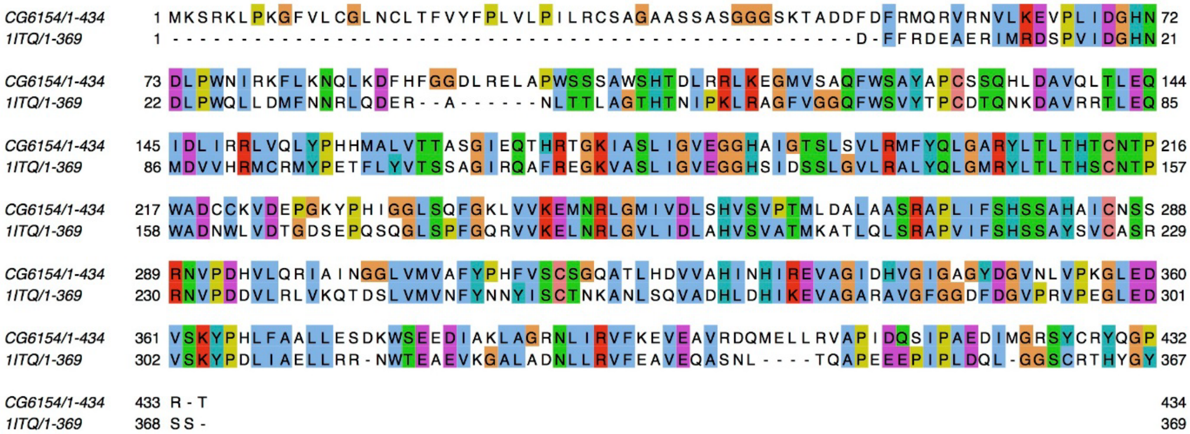

D.

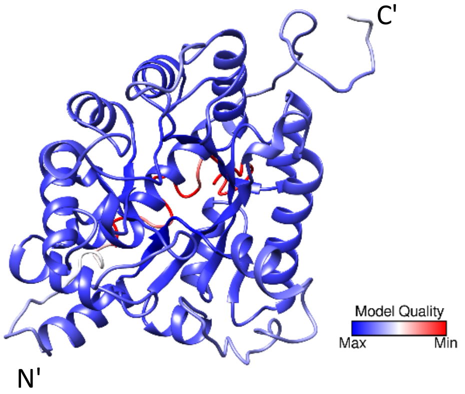

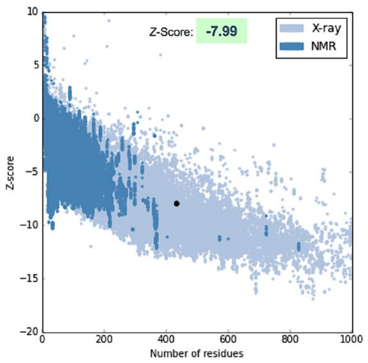

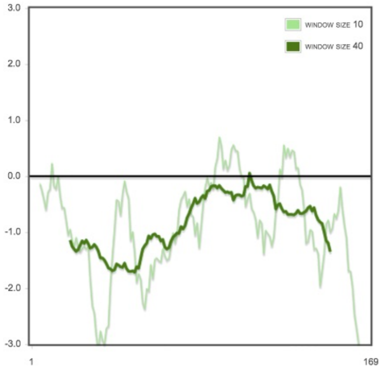

E.

DPEP1 Structure	*D. melanogaster* Model	Superimposed
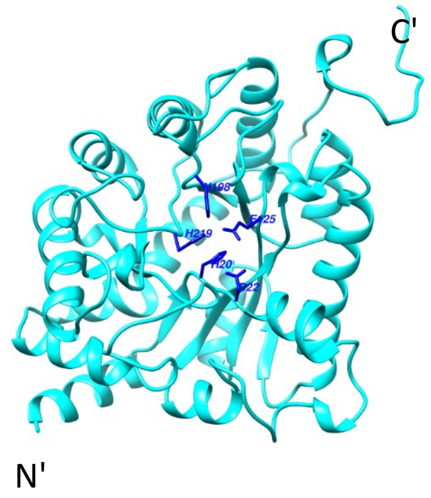	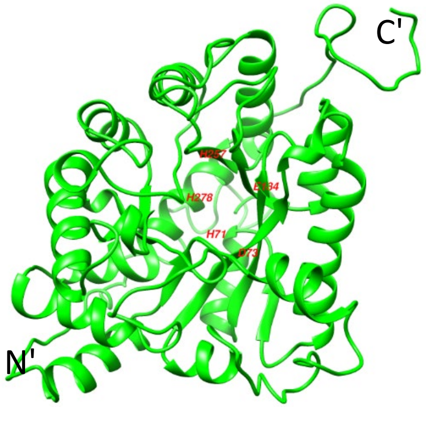	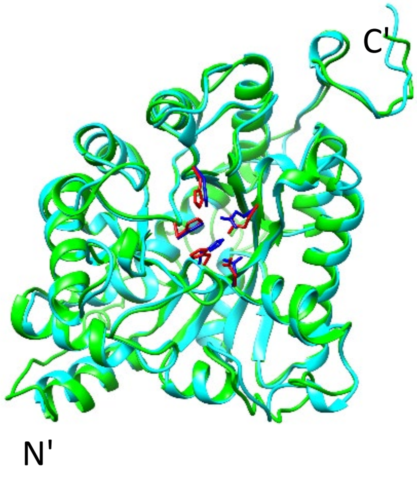

F.	Length (AA)	Domain Architecture (Pfam, range)	Functional Residues (aligned matches in *D. melanogaster*)	Sequence ID%	Structural Overlap (RMSD)
Dipeptidase, renal (DPEP1, NP_004404.1, PDB: 1ITQ)	411	Membrane dipeptidase (PF01244) 31-349	H36, D38, E141, H214 and H235	42% ID 56% SIM	0.523 Å
DPEP (CG6154, NP_733146.2)	434	Membrane dipeptidase (PF01244) 66-393	H71, D73, E184, H257 and H278		

S6 Fig. Sequence and structural details of the modeled fly DPEP1 candidate. A. Domain architecture of DPEP1 and CG6154 and known/predicted functional residues B. Pairwise alignment of CG6154 and 1ITQ generated from structural superposition showing shared secondary structure elements and known/predicted functional residues (marked with red asterisks) C. Pairwise alignment of CG6154 and 1ITQ generated from structural superposition with conserved residues highlighted using the physiochemical color scheme (CLUSTALX) D. Validation of the CG6154 model: ProQ2 quality score mapped to a 3D model of CG6154 (left); ProSA global quality score ranking (middle) and per-residue quality graph (right) E. DPEP1 (1ITQ, cyan-blue) superimposed on the predicted structure of CG6154 (green-red) with potential matches for conserved functional residues highlighted F. Summary of features shared by DPEP1 and potential *D. melanogaster* ortholog CG6154.

.
