# Supplementary material for: A combined computational strategy of sequence and structural analysis predicts the existence of a functional eicosanoid pathway in Drosophila melanogaster
Source: PLoS One. 2019 Feb 12;14(2):e0211897. doi: 10.1371/journal.pone.0211897 (PMC6372189; doi:10.1371/journal.pone.0211897)
Supplement: S7 Fig — A. Domain architecture of GPX1 and CG12013 and known/predicted functional residues B. Pairwise alignment of CG12013 and 2F8A generated from structural superposition showing shared secondary structure elements and known/predicted functional residues (marked with red asterisk; in the GPX1 crystal structure, the selenocysteine is mutated to a glycine) C. Pairwise alignment of CG12013 and 2F8A generated from structural superposition with conserved residues highlighted using the physiochemical color scheme (CLUSTALX) D. Validation of the CG12013 model: ProQ2 quality score mapped to a 3D model of CG12013 (left); ProSA global quality score ranking (middle) and per-residue quality graph (right) E. GPX1 (2F8A, cyan-blue) superimposed on the predicted structure of CG12013 (green-red) with potential matches for conserved functional residues highlighted F. Summary of features shared by GPX1 and potential D. melanogaster ortholog CG12013. (PDF) [file pone.0211897.s007.pdf]

A.

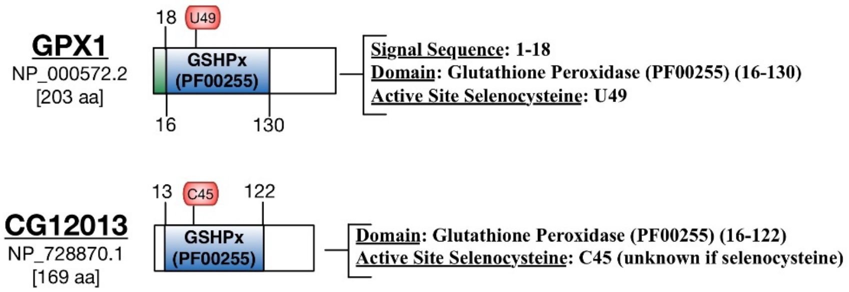
GPX1
NP_000572.2
[203 aa]
18
U49
GSHPx
(PF00255)
16
130
Signal Sequence: 1-18
Domain: Glutathione Peroxidase (PF00255) (16-130)
Active Site Selenocysteine: U49
CG12013
NP_728870.1
[169 aa]
13
C45
122
GSHPx
(PF00255)
Domain: Glutathione Peroxidase (PF00255) (16-122)
Active Site Selenocysteine: C45 (unknown if selenocysteine)

B.

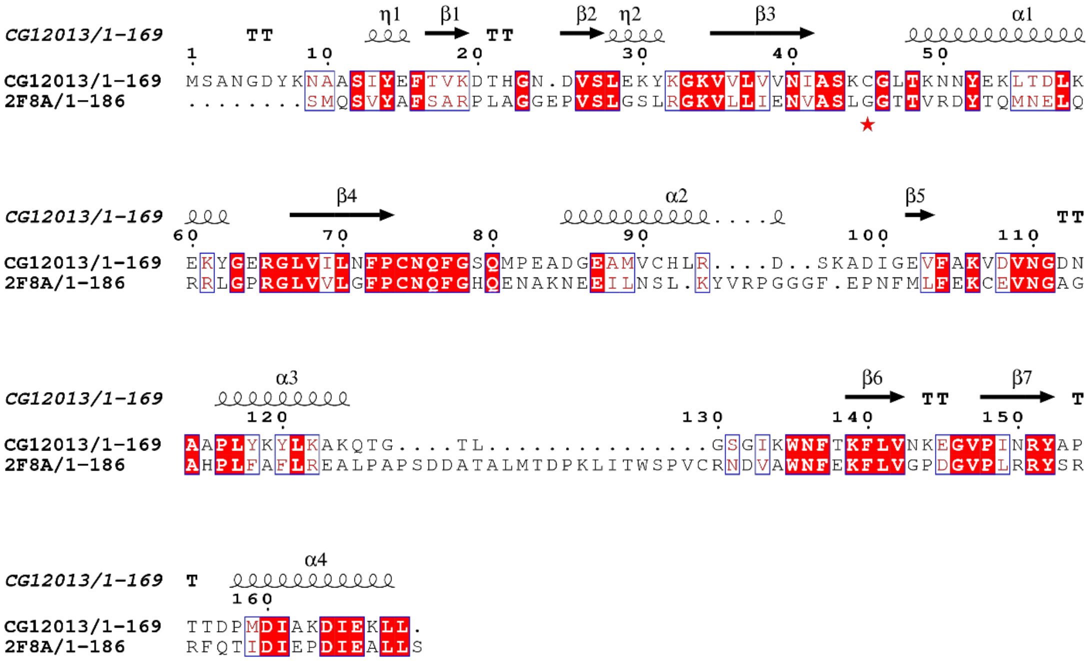
CG12013/1-169
CG12013/1-169 MSANGDYKNAASIYEFTVKDTHGN.DVSLEKYKGKVVLVVNIASKCGLTKNNYEKLTDLK
2F8A/1-186 ........SMQSVYAFSARPLAGGEPVSLGSLRGKVLLIENVASLGGTTVRDYTQMNELQ
CG12013/1-169 EKYGERGLVILNFPCNQFGSQMPEADGEAMVCHLR....D..SKADIGEVFAKVDVNGDN
2F8A/1-186 RRLGPRGLVVLGFPCNQFGHQENAKNEEILNSL.KYVRPGGGF.EPNFMLFEKCEVNGAG
CG12013/1-169 AAPLYKYLKAKQTG....TL...............GSGIKWNFTKFLVNKEGVPINRYAP
2F8A/1-186 AHPLFAFLREALPAPSDDATALMTDPKLITWSPVCRNDVAWNFEKFLVGPDGVPLRRYSR
CG12013/1-169 TTDPMDIAKDIEKLL.
2F8A/1-186 RFQTIDIEPDIEALLS

C.

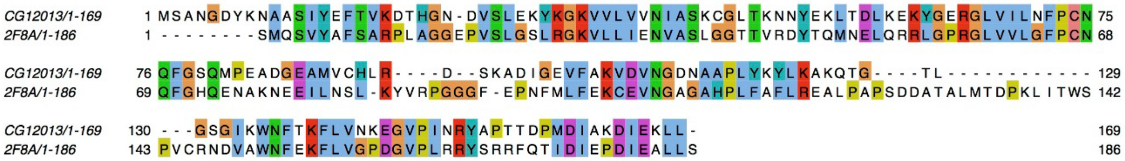
CG12013/1-169 1 MSANGDYKNAASIYEFTVKDTHGN-DVSLEKYKGKVVLVVNIASKCGLTKNNYEKLTDLKEKYGERGLVILNFPCN 75
2F8A/1-186 1 --------SMQSVYAFSARPLAGGEPVSLGSLRGKVLLIENVASLGGTTVRDYTQMNELQRRLGPRGLVVLGFPCN 68
CG12013/1-169 76 QFGSQMPEADGEAMVCHLR----D--SKADIGEVFAKVDVNGDNAAPLYKYLKAKQTG----TL------------- 129
2F8A/1-186 69 QFGHQENAKNEEILNSL-KYVRPGGGF-EPNFMLFEKCEVNGAGAHPLFAFLREALPAPSDDATALMTDPKLITWS 142
CG12013/1-169 130 ---GSGIKWNFTKFLVNKEGVPINRYAPTTDPMDIAKDIEKLL- 169
2F8A/1-186 143 PVCRNDVAWNFEKFLVGPDGVPLRRYSRFQTIDIEPDIEALLS 186

D.

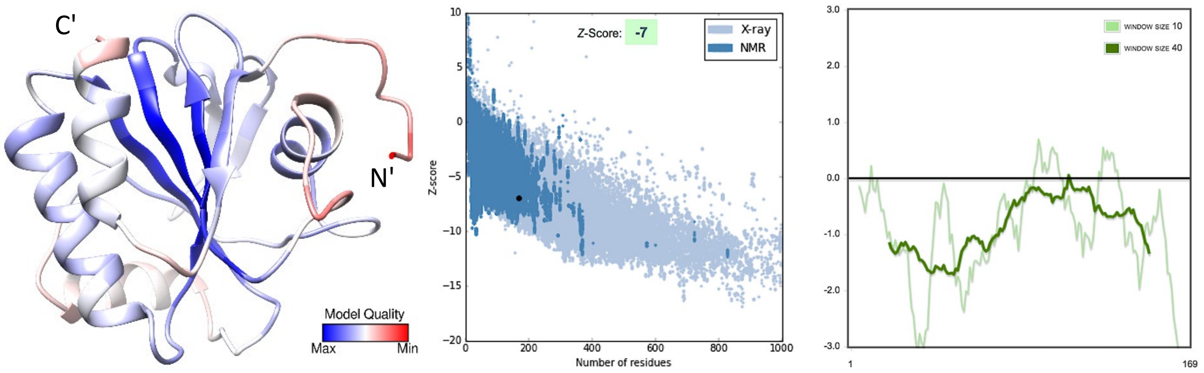

E.

**GPX1 Structure**	***D. melanogaster* Model**	**Superimposed**
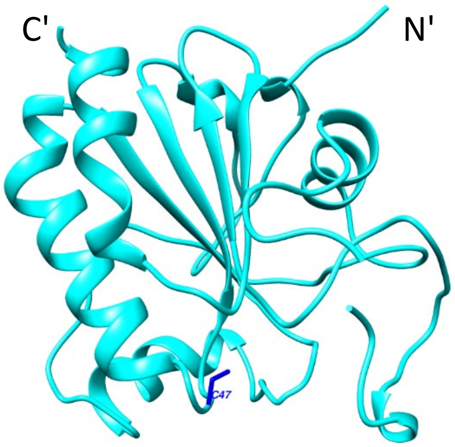	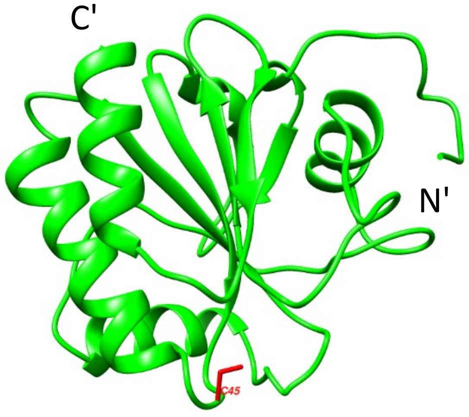	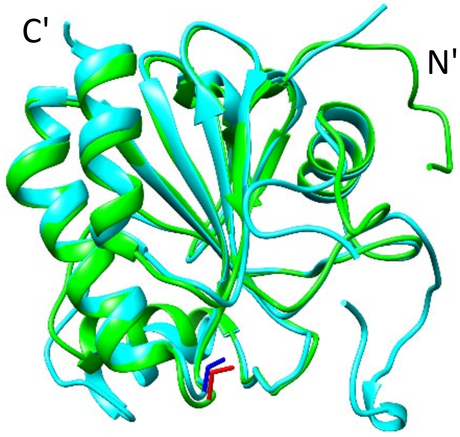

F.

	Length (AA)	Domain Architecture (Pfam, range)	Functional Residues (aligned matches in *D. melanogaster*)	Sequence ID%	Structural Overlap (RMSD)
Glutathione peroxidase 1 (GPX1, NP_000572.2, PDB: 2F8A)	203	GSHPx (PF00255) 16-130	C49	32% ID 44% SIM	0.699Å
Glutathione peroxidase (CG12013, NP_728870.1)	169	GSHPx (PF00255) 13-122	C45		

S7 Fig. Sequence and structural details of the modeled fly GPX1 candidate. A. Domain architecture of GPX1 and CG12013 and known/predicted functional residues B. Pairwise alignment of CG12013 and 2F8A generated from structural superposition showing shared secondary structure elements and known/predicted functional residues (marked with red asterisk; in the GPX1 crystal structure, the selenocysteine is mutated to a glycine) C. Pairwise alignment of CG12013 and 2F8A generated from structural superposition with conserved residues highlighted using the physiochemical color scheme (CLUSTALX) D. Validation of the CG12013 model: ProQ2 quality score mapped to a 3D model of CG12013 (left); ProSA global quality score ranking (middle) and per-residue quality graph (right) E. GPX1 (2F8A, cyan-blue) superimposed on the predicted structure of CG12013 (green-red) with potential matches for conserved functional residues highlighted F. Summary of features shared by GPX1 and potential *D. melanogaster* ortholog CG12013.

.
