# Supplementary material for: A combined computational strategy of sequence and structural analysis predicts the existence of a functional eicosanoid pathway in Drosophila melanogaster
Source: PLoS One. 2019 Feb 12;14(2):e0211897. doi: 10.1371/journal.pone.0211897 (PMC6372189; doi:10.1371/journal.pone.0211897)
Supplement: S8 Fig — A. Domain architecture of CPA1 and CG18585 and known/predicted functional residues B. Pairwise alignment of CG18585 and 3FJU generated from structural superposition showing shared secondary structure elements and known/predicted functional residues (marked with red asterisks) C. Pairwise alignment of CG18585 and 3FJU generated from structural superposition with conserved residues highlighted using the physiochemical color scheme (CLUSTALX) D. Validation of the CG18585 model: ProQ2 quality score mapped to a 3D model of CG18585 (left); ProSA global quality score ranking (middle) and per-residue quality graph (right) E. CPA1 (3FJU, cyan-blue) superimposed on the predicted structure of CG18585 (green-red) with potential matches for conserved functional residues highlighted F. Summary of features shared by CPA1 and potential D. melanogaster ortholog CG18585. (PDF) [file pone.0211897.s008.pdf]

A.

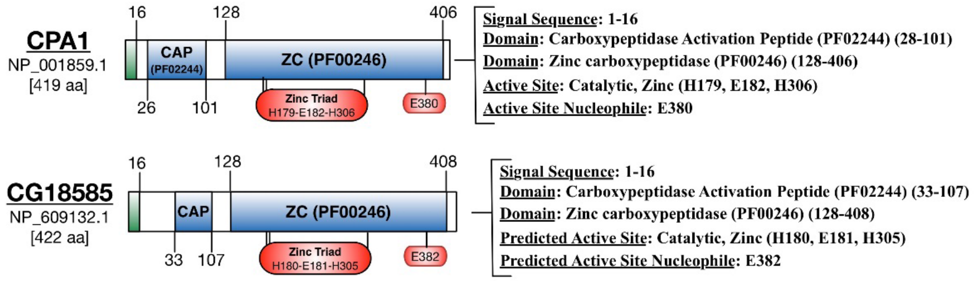

CPA1
NP_001859.1
[419 aa]
16
128
406
26
101
CAP
(PF02244)
ZC (PF00246)
Zinc Triad
H179-E182-H306
E380
Signal Sequence: 1-16
Domain: Carboxypeptidase Activation Peptide (PF02244) (28-101)
Domain: Zinc carboxypeptidase (PF00246) (128-406)
Active Site: Catalytic, Zinc (H179, E182, H306)
Active Site Nucleophile: E380
CG18585
NP_609132.1
[422 aa]
16
128
408
33
107
CAP
ZC (PF00246)
Zinc Triad
H180-E181-H305
E382
Signal Sequence: 1-16
Domain: Carboxypeptidase Activation Peptide (PF02244) (33-107)
Domain: Zinc carboxypeptidase (PF00246) (128-408)
Predicted Active Site: Catalytic, Zinc (H180, E181, H305)
Predicted Active Site Nucleophile: E382

B.

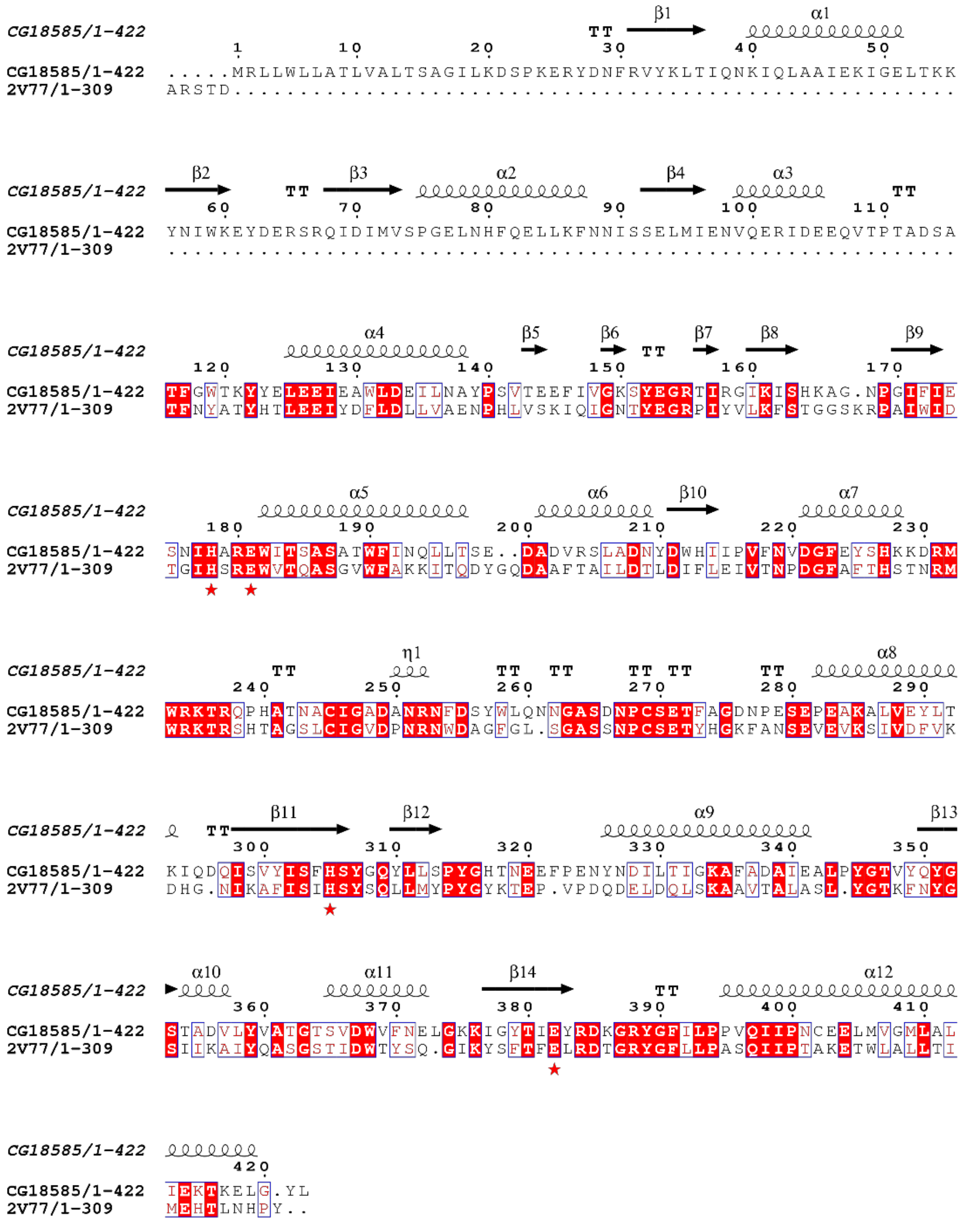

CG18585/1-422 .....MRLLWLLATLVALTSAGILKDSPKERYDNFRVYKLTIQNKIQLAAIEKIGELTKK
2V77/1-309 ARSTD...
CG18585/1-422 YNIWKEYDERSRQIDIMVSPGELNHFQELLKFNNISSELMIENVQERIDEEQVTPTADSA
2V77/1-309 ....
CG18585/1-422 TFGWTKYYELEEIEAWLDEILNAYPSVTEEFIVGKSYEGRTIRGIKISHKAG.NPGIFIE
2V77/1-309 TFNYATYHTLEEIYDFLDLLVAENPHLVSKIQIGNTYEGRPIYVLKFSTGGSKRPAIWID
CG18585/1-422 SNIHAREWITSASATWFINQLLTSE..DADVRSLADNYDWHIIPVFNVDGFEYSHKKDRM
2V77/1-309 TGIHSREWVTQASGVWFAKKITQDYGQDAAFTAILDTLDIFLEIVTNPDGFAFTHSTNRM
CG18585/1-422 WRKTRQPHATNACIGADANRNFDSYWLQNNGASDNPCSETFAGDNPESEPEAKALVEYLT
2V77/1-309 WRKTRSHTAGSLCIGVDPNRNWDAGFGL.SGASSNPCSETYHGKFANSEVEVKSIVDFVK
CG18585/1-422 KIQDQISVYISFHSYGQYLLSPYGHTNEEFPENYNDILTIGKAFADAIEALPYGTVYQYG
2V77/1-309 DHG.NIKAFISIHSYSQLLMYPYGYKTEP.VPDQDELDQLSKAAVTALASL.YGTKFNYG
CG18585/1-422 STADVLYVATGTSVDWVFNELGKKIGYTIEYRDKGRYGFILPPVQIIPNCEELMVGMLAL
2V77/1-309 SIIKAIYQASGSTIDWTYSQ.GIKYSFTFELRDTGRYGFLLPASQIIPTAKETWLALLTI
CG18585/1-422 IEKTKELG.YL
2V77/1-309 MEHTLNHPY..

C.

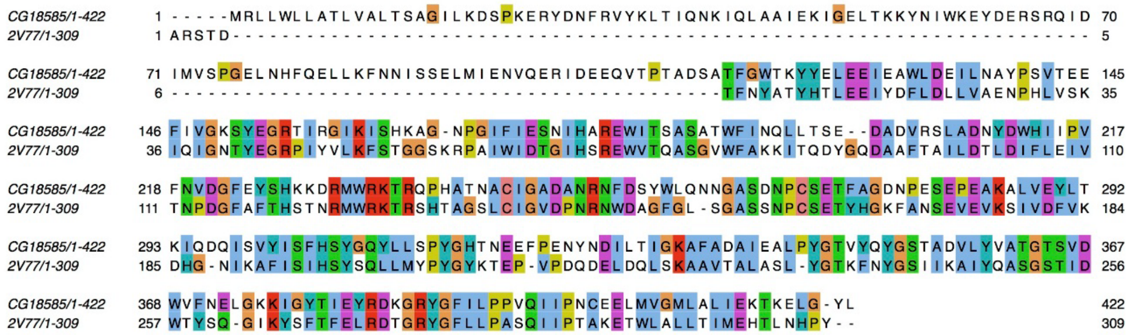
CG18585/1-422 1 -----MRLLWLLATLVALTSAGILKDSPKERYDNFRVYKLTIQNKIQLAAIEKIGELTKKYNIWKEYDERSRQID 70
2V77/1-309 1 ARSTD--- 5
CG18585/1-422 71 IMVSPGELNHFQELLKFNNISSELMIENVQERIDEEQVTPTADSATFGWTKYYELEEIEAWLDEILNAYPSVTEE 145
2V77/1-309 6 ---TFNYATYHTLEEIYDFLDLLVAENPHLVSK 35
CG18585/1-422 146 FIVGKSYEGRTIRGIKISHKAG-NPGIFIESNIHAREWITSASATWFINQLLTSE--DADVRSLADNYDWHIIPV 217
2V77/1-309 36 IQIGNTYEGRPIYVLKFSTGGSKRPAIWIDTGIHSREWVTQASGVWFAKKITQDYGQDAAFTAILDTLDIFLEIV 110
CG18585/1-422 218 FNVDGFEYSHKKDRMWRKTRQPHATNACIGADANRNFDSYWLQNNGASDNPCSETFAGDNPESEPEAKALVEYLT 292
2V77/1-309 111 TNPDGFAFTHSTNRMWRKTRSHTAGSLCIGVDPNRNWDAGFGL-SGASSNPCSETYHGKFANSEVEVKSIVDFVK 184
CG18585/1-422 293 KIQDQISVYISFHSYGQYLLSPYGHTNEEFPENYNDILTIGKAFADAIEALPYGTVYQYGSTADVLYVATGTSVD 367
2V77/1-309 185 DHG-NIKAFISIHSYSQLLMYPYGYKTEP-VPDQDELDQLSKAAVTALASL-YGTKFNYGSIIKAIYQASGSTID 256
CG18585/1-422 368 WVFNELGKKIGYTIEYRDKGRYGFILPPVQIIPNCEELMVGMLALIEKTKELG-YL 422
2V77/1-309 257 WTYSQ-GIKYSFTFELRDTGRYGFLLPASQIIPTAKETWLALLTIMEHTLNHPY-- 309

D.

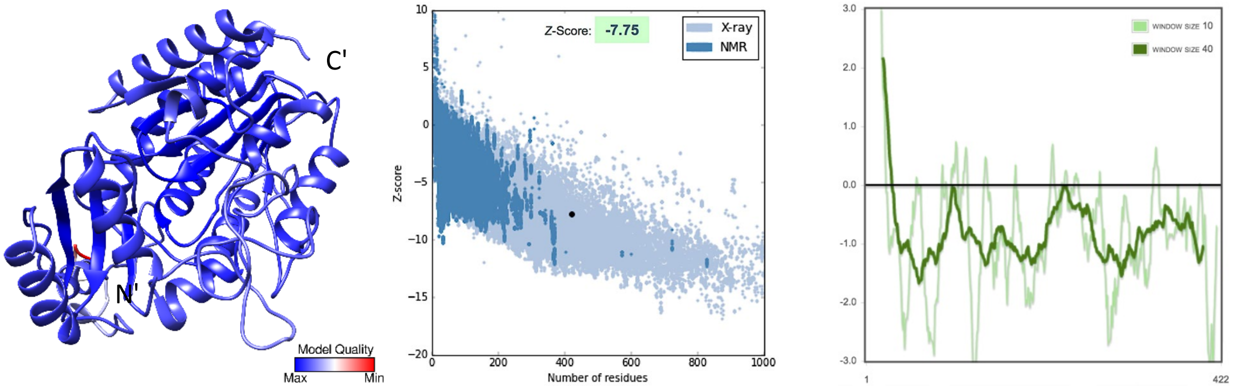
C'
N'
Model Quality
Max
Min
Z-Score: -7.75
X-ray
NMR
Z-score
Number of residues
WINDOW SIZE 10
WINDOW SIZE 40

E.

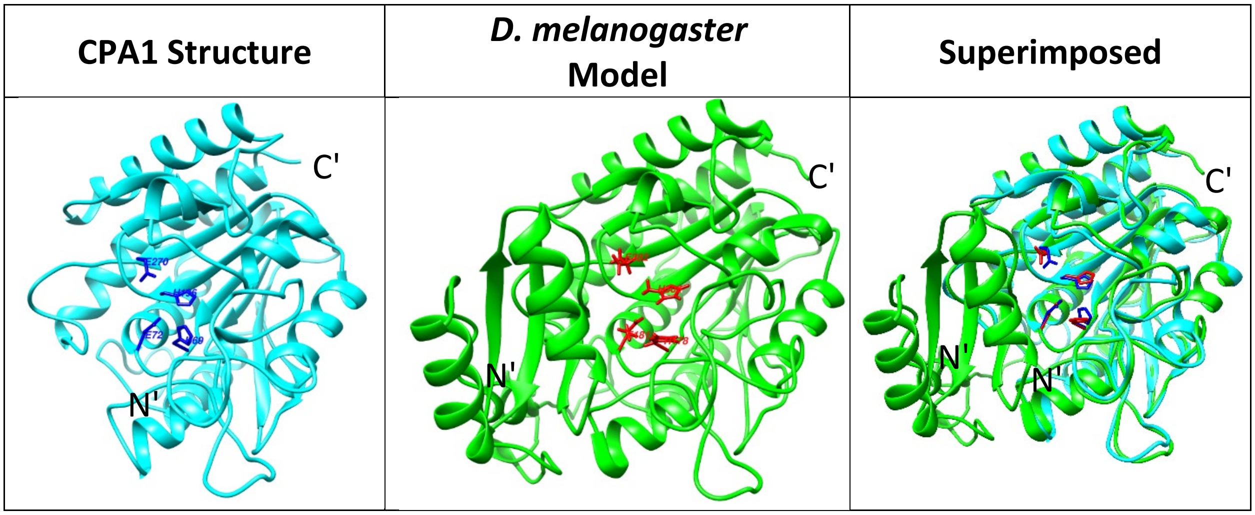
CPA1 Structure
D. melanogaster Model
Superimposed
C'
N'

F.	Length (AA)	Domain Architecture (Pfam, range)	Functional Residues (aligned matches in *D. melanogaster*)	Sequence ID%	Structural Overlap (RMSD)
Carboxypeptidase A1 (CPA1, NP_001859.1, PDB: 3FJU)	419	Carboxypeptidase activation peptide (PF02244) 26-100<br>Zinc carboxypeptidase (PF00246) 128-406	H179, E182, H306 and E380	34% ID 54% SIM	0.753 Å
CPA (CG18585, NP_609132.1)	422	Carboxypeptidase activation peptide (PF02244) 33-106<br>Zinc carboxypeptidase (PF00246) 128-408	H178, E181, H305 and E382		

S8 Fig. Sequence and structural details of the modeled fly CPA1 candidate. A. Domain architecture of CPA1 and CG18585 and known/predicted functional residues B. Pairwise alignment of CG18585 and 3FJU generated from structural superposition showing shared secondary structure elements and known/predicted functional residues (marked with red asterisks) C. Pairwise alignment of CG18585 and 3FJU generated from structural superposition with conserved residues highlighted using the physiochemical color scheme (CLUSTALX) D. Validation of the CG18585 model: ProQ2 quality score mapped to a 3D model of CG18585 (left); ProSA global quality score ranking (middle) and per-residue quality graph (right) E. CPA1 (3FJU, cyan-blue) superimposed on the predicted structure of CG18585 (green-red) with potential matches for conserved functional residues highlighted F. Summary of features shared by CPA1 and potential *D. melanogaster* ortholog CG18585.
