# Supplementary material for: A combined computational strategy of sequence and structural analysis predicts the existence of a functional eicosanoid pathway in Drosophila melanogaster
Source: PLoS One. 2019 Feb 12;14(2):e0211897. doi: 10.1371/journal.pone.0211897 (PMC6372189; doi:10.1371/journal.pone.0211897)
Supplement: S9 Fig — A. Domain architecture of HPGDS and CG8938 and known/predicted functional residues B. Pairwise alignment of CG8938 and 1IYI generated from structural superposition showing shared secondary structure elements and known/predicted functional residues (marked with red asterisks) C. Pairwise alignment of CG8938 and 1IYI generated from structural superposition with conserved residues highlighted using the physiochemical color scheme (CLUSTALX) D. Validation of the CG8938 model: ProQ2 quality score mapped to a 3D model of CG8938 (left); ProSA global quality score ranking (middle) and per-residue quality graph (right) E. CPA1 (1IYI, cyan-blue) superimposed on the predicted structure of CG8938 (green-red) with potential matches for conserved functional residues highlighted F. Summary of features shared by HPGDS and potential D. melanogaster ortholog CG8938. (PDF) [file pone.0211897.s009.pdf]

A.

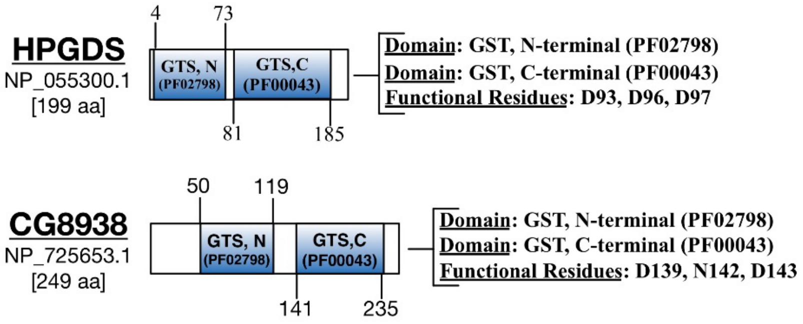
HPGDS
NP_055300.1
[199 aa]
4
73
81
185
GTS, N (PF02798)
GTS,C (PF00043)
Domain: GST, N-terminal (PF02798)
Domain: GST, C-terminal (PF00043)
Functional Residues: D93, D96, D97
CG8938
NP_725653.1
[249 aa]
50
119
141
235
GTS, N (PF02798)
GTS,C (PF00043)
Domain: GST, N-terminal (PF02798)
Domain: GST, C-terminal (PF00043)
Functional Residues: D139, N142, D143

B.

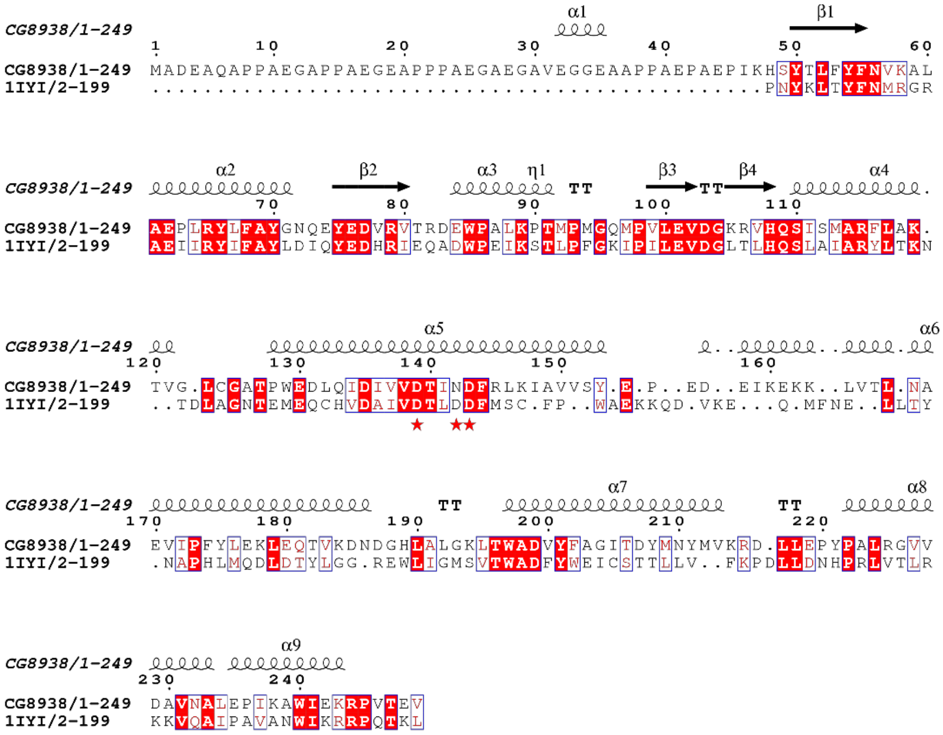
CG8938/1-249
1IYI/2-199
α1
β1
α2
β2
α3
η1
β3
β4
α4
α5
α6
α7
α8
α9
TT

C.

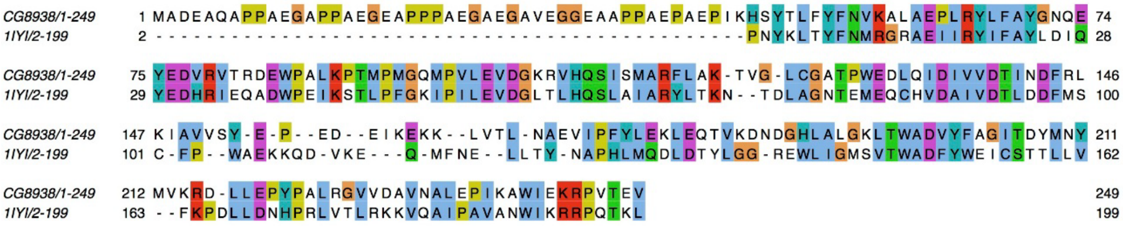
CG8938/1-249 1 MADEAQAPPAEGAPPAEGEAPPPAEGAEGAVEGGEAAPPAEPAEPIKHSYTLFYFNVKALAEPLRYLFAYGNQE 74
1IYI/2-199 2 --PNYKLTYFNMRGRAEIIRYIFAYLDIQ 28
CG8938/1-249 75 YEDVRVTRDEWPALKPTMPMGQMPVLEVDGKRVHQSISMARFLAK-TVG-LCGATPWEDLQIDIVVDTINDFRL 146
1IYI/2-199 29 YEDHRIEQADWPEIKSTLPFGKIPILEVDGLTLHQSLAIARYLTKN--TDLAGNTEMEQCHVDAIVDTLDDFMS 100
CG8938/1-249 147 KIAVVSY-E-P--ED--EIKEKK--LVTL-NAEVIPFYLEKLEQTVKDNDGHLALGKLTWADVYFAGITDYMNY 211
1IYI/2-199 101 C-FP--WAEKKQD-VKE---Q-MFNE--LLTY-NAPHLMQDLDTYLGG-REWLIGMSVTWADFYWEICSTTLLV 162
CG8938/1-249 212 MVKRD-LLEPYPALRGVVDAVNALEPIKAWIEKRPVTEV 249
1IYI/2-199 163 --FKPDLLDNHPRLVTLRKKVQAIPAVANWIKRRPQTKL 199

D.

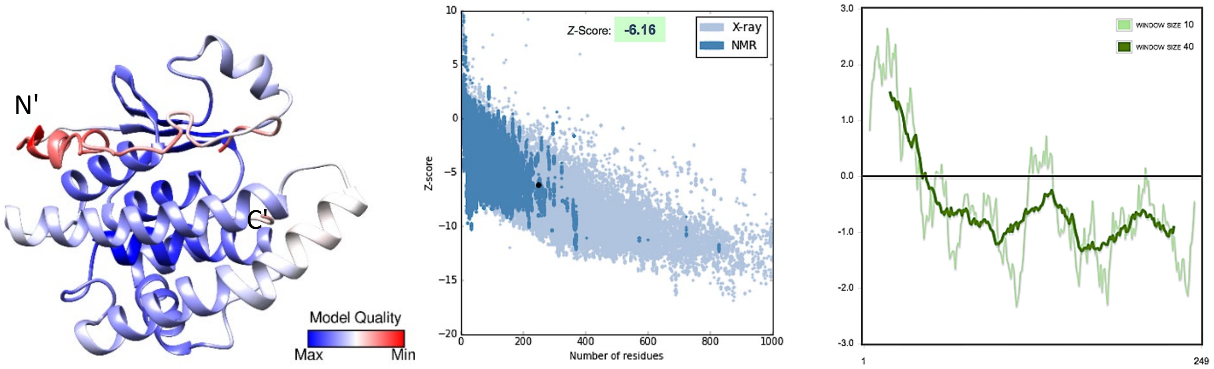

E.

**HPGDS Structure**	***D. melanogaster* Model**	**Superimposed**
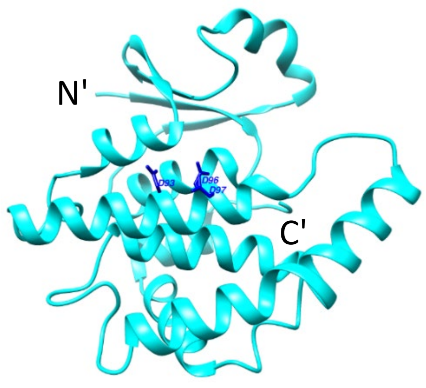	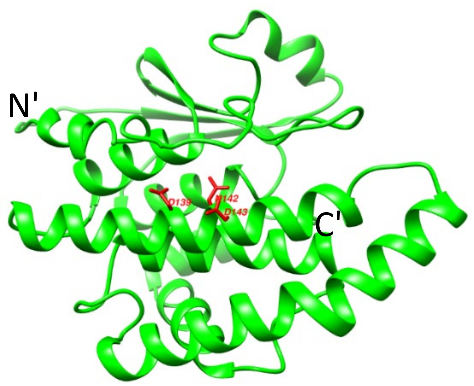	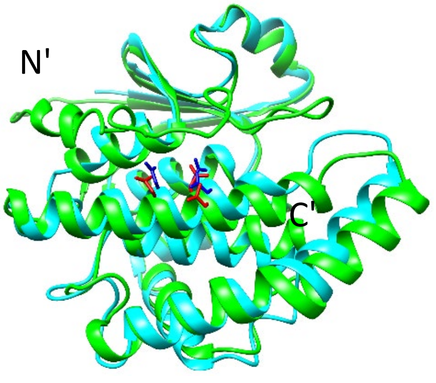

F.

	Length (AA)	Domain Architecture (Pfam, range)	Functional Residues (aligned matches in *D. melanogaster*)	Sequence ID%	Structural Overlap (RMSD)
Hematopoietic Prostaglandin D synthase (HPGDS, NP_055300.1, PDB: 1IYI)	160	GST-N domain (PF02798) 4-73 GST-C domain (PF00043) 81-185	D93, D96, D97	27% ID 43% SIM	1.045 Å
Glutathione S transferase S1 (CG8938, NP_725653.1)	184	GST-N domain (PF02798) 50-119 GST-C domain (PF00043) 141-235	D139, D143, N142		

S9 Fig. Sequence and structural details of the modeled fly HPGDS candidate. A. Domain architecture of HPGDS and CG8938 and known/predicted functional residues B. Pairwise alignment of CG8938 and 1IYI generated from structural superposition showing shared secondary structure elements and known/predicted functional residues (marked with red asterisks) C. Pairwise alignment of CG8938 and 1IYI generated from structural superposition with conserved residues highlighted using the physiochemical color scheme (CLUSTALX) D. Validation of the CG8938 model: ProQ2 quality score mapped to a 3D model of CG8938 (left); ProSA global quality score ranking (middle) and per-residue quality graph (right) E. CPA1 (1IYI, cyan-blue) superimposed on the predicted structure of CG8938 (green-red) with potential matches for conserved functional residues highlighted F. Summary of features shared by HPGDS and potential *D. melanogaster* ortholog CG8938.
