# Supplementary material for: A combined computational strategy of sequence and structural analysis predicts the existence of a functional eicosanoid pathway in Drosophila melanogaster
Source: PLoS One. 2019 Feb 12;14(2):e0211897. doi: 10.1371/journal.pone.0211897 (PMC6372189; doi:10.1371/journal.pone.0211897)
Supplement: S10 Fig — A. Domain architecture of PTGES3 and CG16817 and known/predicted functional residues B. Pairwise alignment of CG16817 and 1EFJ generated from structural superposition showing shared secondary structure elements C. Pairwise alignment of CG16817 and 1EFJ generated from structural superposition with conserved residues highlighted using the physiochemical color scheme (CLUSTALX) D. Validation of the CG16817 model: ProQ2 quality score mapped to a 3D model of CG16817 (left); ProSA global quality score ranking (middle) and per-residue quality graph (right) E. CPA1 (1EFJ, cyan-blue) superimposed on the predicted structure of CG16817 (green-red) with potential matches for conserved functional residues highlighted F. Summary of features shared by PTGES3 and potential D. melanogaster ortholog CG16817. (PDF) [file pone.0211897.s010.pdf]

A.

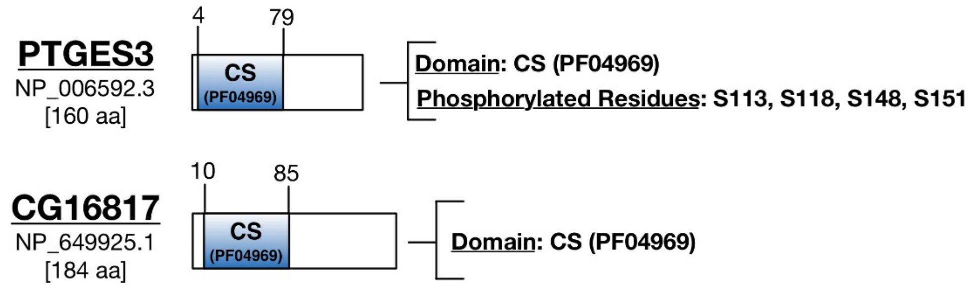
PTGES3
NP_006592.3
[160 aa]
4
79
CS
(PF04969)
Domain: CS (PF04969)
Phosphorylated Residues: S113, S118, S148, S151
CG16817
NP_649925.1
[184 aa]
10
85
CS
(PF04969)
Domain: CS (PF04969)

B.

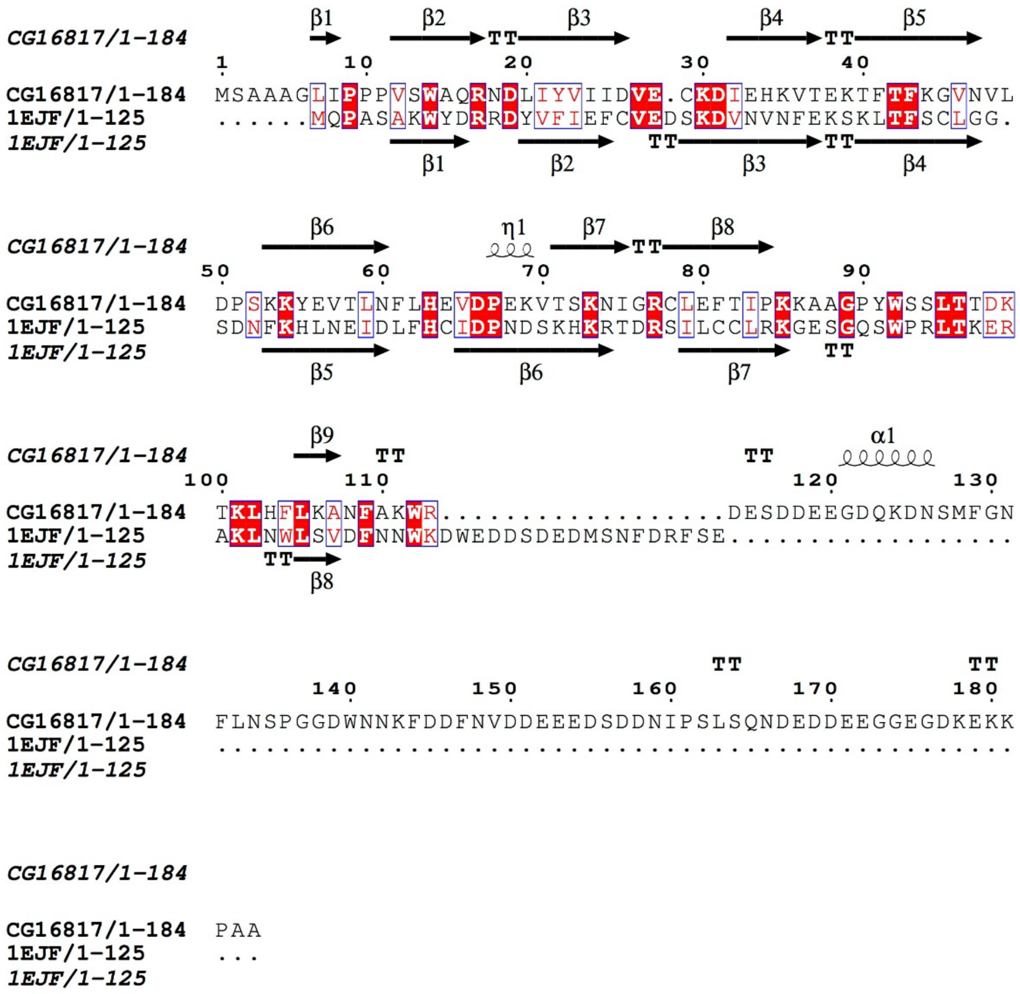
CG16817/1-184
β1 β2 β3 β4 β5
1 10 20 30 40
CG16817/1-184 MSAAAGLIPPPVSWAQRNDLIYVIIDVE.CKDIEHKVTEKTFTFKGVNVL
1EJF/1-125 ......MQPASAKWYDRRDYVFIEFCVEDSKDVNVNFEKSKLTFSCLGG.
1EJF/1-125
β1 β2 β3 β4
CG16817/1-184
β6 η1 β7 β8
50 60 70 80 90
CG16817/1-184 DPSKKYEVTLNFLHEVDPEKVTSKNIGRCLEFTIPKKAAGPYWSSLTTDK
1EJF/1-125 SDNFKHLNEIDLFHCIDPNDSKHKRTDRSILCCLRKGESGQSWPRLTKER
1EJF/1-125
β5 β6 β7
CG16817/1-184
β9 α1
100 110 120 130
CG16817/1-184 TKLHFLKANFAKWR..................DESDDEEGDQKDNSMFGN
1EJF/1-125 AKLNWLSVDFNNWKDWEDDSDEDMSNFDRFSE..................
1EJF/1-125
β8
CG16817/1-184
140 150 160 170 180
CG16817/1-184 FLNSPGGDWNNKFDDFNVDDEEEDSDDNIPSLSQNDEDDEEGGEGDKEKK
1EJF/1-125 ....
1EJF/1-125
CG16817/1-184
CG16817/1-184 PAA
1EJF/1-125 ...
1EJF/1-125

C.

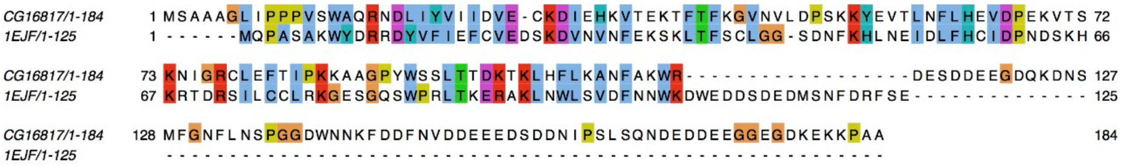
CG16817/1-184 1 MSAAAGLIPPPVSWAQRNDLIYVIIDVE-CKDIEHKVTEKTFTFKGVNVLDPSKKYEVTLNFLHEVDPEKVTS 72
1EJF/1-125 1 ------MQPASAKWYDRRDYVFIEFCVEDSKDVNVNFEKSKLTFSCLGG-SDNFKHLNEIDLFHCIDPNDSKH 66
CG16817/1-184 73 KNIGRCLEFTIPKKAAGPYWSSLTTDKTKLHFLKANFAKWR------------------DESDDEEGDQKDNS 127
1EJF/1-125 67 KRTDRSILCCLRKGESGQSWPRLTKERAKLNWLSVDFNNWKDWEDDSDEDMSNFDRFSE------------------ 125
CG16817/1-184 128 MFGNFLNSPGGDWNNKFDDFNVDDEEEDSDDNIPSLSQNDEDDEEGGEGDKEKKPAA 184
1EJF/1-125

D.

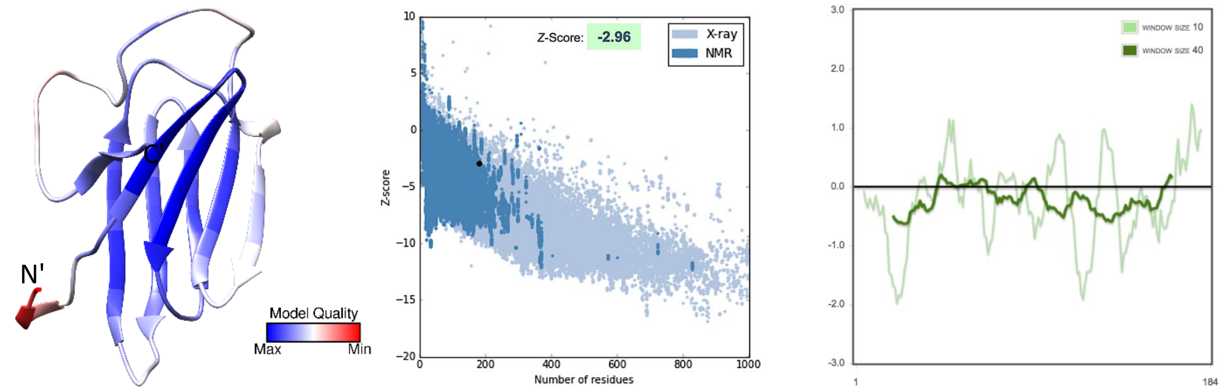

E.

Truncated PTGES3 Structure	*D. melanogaster* Model (Partial)	Superimposed
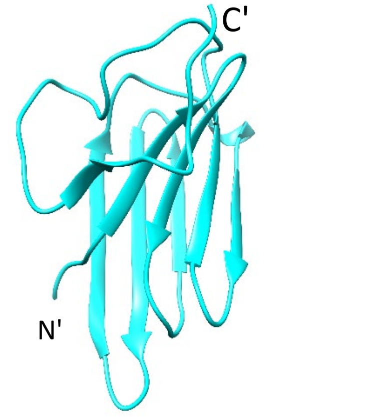	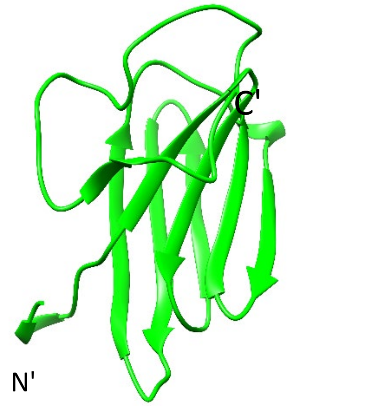	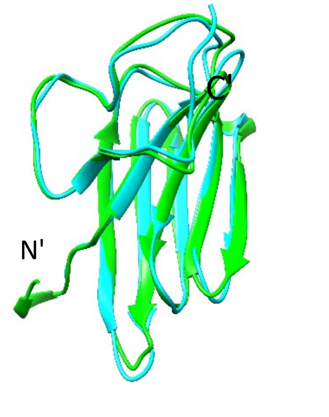

F.

	Length (AA)	Domain Architecture (Pfam, range)	Functional Residues (aligned matches in *D. melanogaster*)	Sequence ID%	Structural Overlap (RMSD)
Prostaglandin E synthase 3 (PTGES3, NP_006592.3, PDB: 1EJF)	160	CS Domain (PF04969) 4-79	N/A	24% ID 40% SIM	0.823 Å
Uncharacterized protein (CG16817, NP_649925.1)	184	CS Domain (PF04969) 10-85	N/A		

S10 Fig. Sequence and structural details of the modeled fly PTGES3 candidate. A. Domain architecture of PTGES3 and CG16817 and known/predicted functional residues B. Pairwise alignment of CG16817 and 1EFJ generated from structural superposition showing shared secondary structure elements C. Pairwise alignment of CG16817 and 1EFJ generated from structural superposition with conserved residues highlighted using the physiochemical color scheme (CLUSTALX) D. Validation of the CG16817 model: ProQ2 quality score mapped to a 3D model of CG16817 (left); ProSA global quality score ranking (middle) and per-residue quality graph (right) E. CPA1 (1EFJ, cyan-blue) superimposed on the predicted structure of CG16817 (green-red) with potential matches for conserved functional residues highlighted F. Summary of features shared by PTGES3 and potential *D. melanogaster* ortholog CG16817.
