# Supplementary material for: A combined computational strategy of sequence and structural analysis predicts the existence of a functional eicosanoid pathway in Drosophila melanogaster
Source: PLoS One. 2019 Feb 12;14(2):e0211897. doi: 10.1371/journal.pone.0211897 (PMC6372189; doi:10.1371/journal.pone.0211897)
Supplement: S11 Fig — A. Domain architecture of CBR1 and CG11200 and known/predicted functional residues B. Pairwise alignment of CG11200 and 3BHJ generated from structural superposition showing shared secondary structure elements and known/predicted functional residues (marked with red asterisks) C. Pairwise alignment of CG11200 and 3BHJ generated from structural superposition with conserved residues highlighted using the physiochemical color scheme (CLUSTALX) D. Validation of the CG11200 model: ProQ2 quality score mapped to a 3D model of CG11200 (left); ProSA global quality score ranking (middle) and per-residue quality graph (right) E. CPA1 (3BHJ, cyan-blue) superimposed on the predicted structure of CG11200 (green-red) with potential matches for conserved functional residues highlighted F. Summary of features shared by CBR1 and potential D. melanogaster ortholog CG11200. (PDF) [file pone.0211897.s011.pdf]

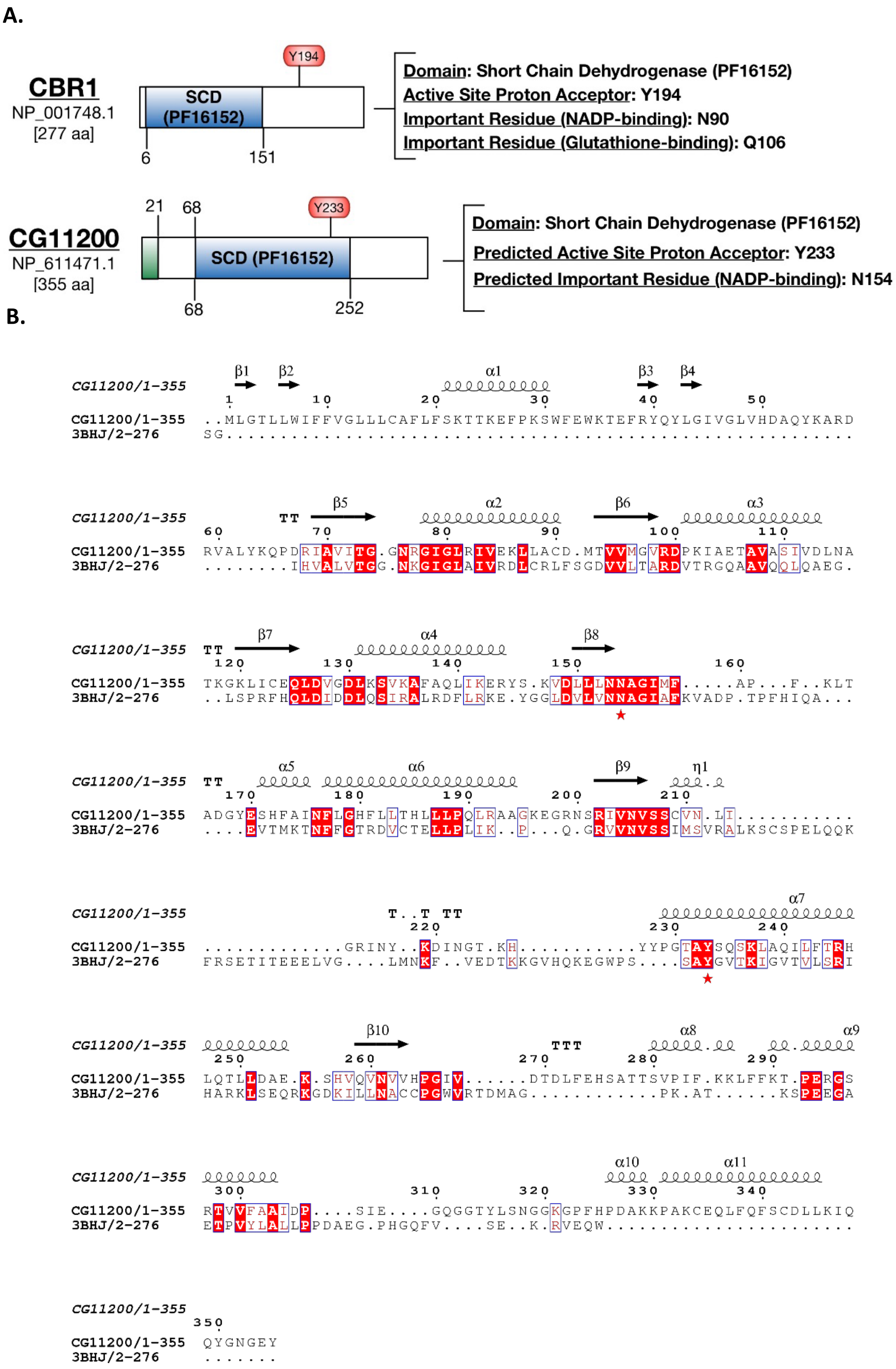

A.
CBR1
NP_001748.1
[277 aa]
SCD (PF16152)
Y194
6
151
Domain: Short Chain Dehydrogenase (PF16152)
Active Site Proton Acceptor: Y194
Important Residue (NADP-binding): N90
Important Residue (Glutathione-binding): Q106
CG11200
NP_611471.1
[355 aa]
21
68
SCD (PF16152)
Y233
68
252
Domain: Short Chain Dehydrogenase (PF16152)
Predicted Active Site Proton Acceptor: Y233
Predicted Important Residue (NADP-binding): N154
B.
CG11200/1-355 ..MLGTLLWIFFVGLLLCAFLFSKTTKEFPKSWFEWKTEFRYQYLGIVGLVHDAQYKARD
3BHJ/2-276 SG..
CG11200/1-355 RVALYKQPDRIAVITG.GNRGIGLRIVEKLLACD.MTVVMGVRDPKIAETAVASIVDLNA
3BHJ/2-276 ........IHVALVTGG.NKGIGLAIVRDLCRLFSGDVVLTARDVTRGQAAVQQLQAEG.
CG11200/1-355 TKGKLICEQLDVGDLKSVKAFAQLIKERYS.KVDLLNNAGIMF.....AP...F..KLT
3BHJ/2-276 ..LSPRFHQLDIDDLQSIRALRDFLRKE.YGGLDVLVNNAGIAFKVADP.TPFHIQA...
CG11200/1-355 ADGYESHFAINFLGHFLLTHLLLPQLRAAGKEGRNSRIVNVSSCVN.LI...........
3BHJ/2-276 ....EVTMKTNFFGTRDVCTELLPLIK..P...Q.GRVVNVSSIMSVRALKSCSPELQQK
CG11200/1-355 .............GRINY..KDINGT.KH...........YYPGTAYSQSKLAQILFTRH
3BHJ/2-276 FRSETITEEELVG....LMNKF..VEDTKKGVHQKEGWPS....SAYGVTKIGVTVLSRI
CG11200/1-355 LQTLLDAE.K.SHVQVNVVHPGIV......DTDLFEHSATTSVPIF.KKLFFKT.PERGS
3BHJ/2-276 HARKLSEQRKGDKILLNACCPGWVRTDMAG...........PK.AT......KSPEEGA
CG11200/1-355 RTVVFAAIDP....SIE....GQGGTYLSNGGKGPFHPDAKKPAKCEQLFQFSCDLLKIQ
3BHJ/2-276 ETPVYLALLPPDAEG.PHGQFV....SE..K.RVEQW.....................
CG11200/1-355 QYGNGEY
3BHJ/2-276 ....

C.

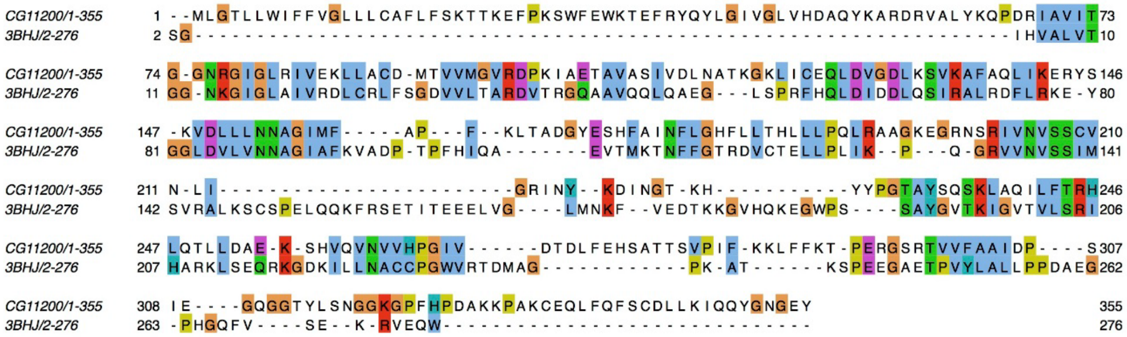

D.

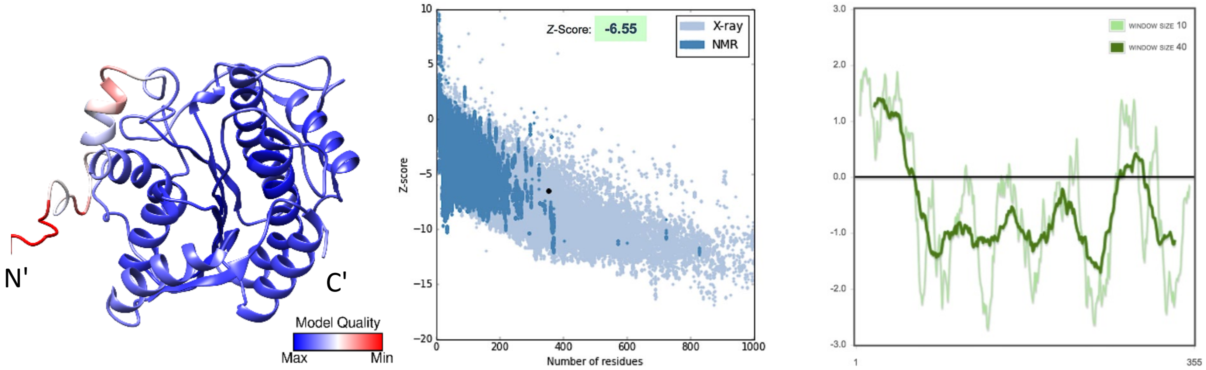

E.

**CBR1 Structure**	***D. melanogaster* Model**	**Superimposed**
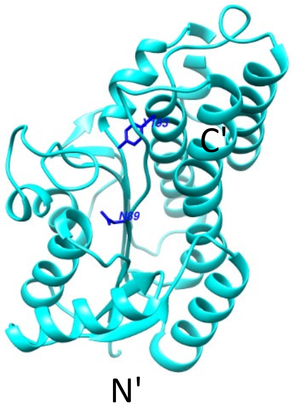	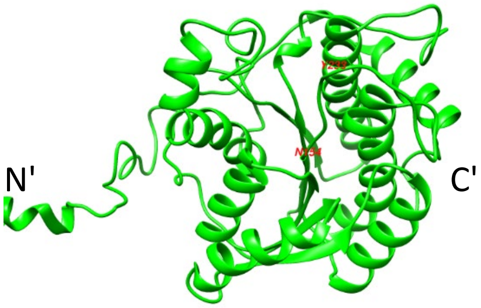	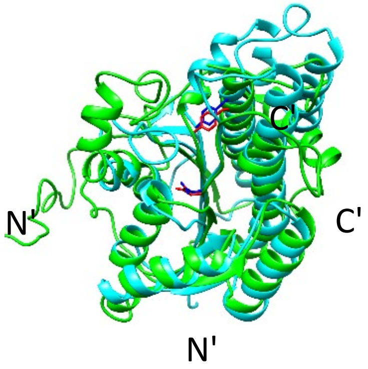

F.	Length (AA)	Domain Architecture (Pfam, range)	Functional Residues (aligned matches in *D. melanogaster*)	Sequence ID%	Structural Overlap (RMSD)
Carbonyl reductase 1 (CBR1, NP_001748.1, PDB: 3BHJ)	277	Short chain dehydrogenase domain (PF16152) 6-151	N90 and Y194	20% ID 33% SIM	1.153 Å
CBR (CG11200, NP_611471.1)	355	Short chain dehydrogenase domain (PF16152) 68-252	N154 and Y233		

S11 Fig. Sequence and structural details of the modeled fly CBR1 candidate. A. Domain architecture of CBR1 and CG11200 and known/predicted functional residues B. Pairwise alignment of CG11200 and 3BHJ generated from structural superposition showing shared secondary structure elements and known/predicted functional residues (marked with red asterisks) C. Pairwise alignment of CG11200 and 3BHJ generated from structural superposition with conserved residues highlighted using the physiochemical color scheme (CLUSTALX) D. Validation of the CG11200 model: ProQ2 quality score mapped to a 3D model of CG11200 (left); ProSA global quality score ranking (middle) and per-residue quality graph (right) E. CPA1 (3BHJ, cyan-blue) superimposed on the predicted structure of CG11200 (green-red) with potential matches for conserved functional residues highlighted F. Summary of features shared by CBR1 and potential *D. melanogaster* ortholog CG11200.
