# Supplementary material for: A combined computational strategy of sequence and structural analysis predicts the existence of a functional eicosanoid pathway in Drosophila melanogaster
Source: PLoS One. 2019 Feb 12;14(2):e0211897. doi: 10.1371/journal.pone.0211897 (PMC6372189; doi:10.1371/journal.pone.0211897)
Supplement: S12 Fig — A. Domain architecture of HPGD and CG18814 and known/predicted functional residues B. Pairwise alignment of CG18814 and 2GDZ generated from structural superposition showing shared secondary structure elements and known/predicted functional residues (marked with red asterisks) C. Pairwise alignment of CG18814 and 2GDZ generated from structural superposition with conserved residues highlighted using the physiochemical color scheme (CLUSTALX) D. Validation of the CG18814 model: ProQ2 quality score mapped to a 3D model of CG18814 (left); ProSA global quality score ranking (middle) and per-residue quality graph (right) E. HPGD (2GDZ, cyan-blue) superimposed on the predicted structure of CG18814 (green-red) with potential matches for conserved functional residues highlighted F. Summary of features shared by HPGD and potential D. melanogaster ortholog CG18814. (PDF) [file pone.0211897.s012.pdf]

A.

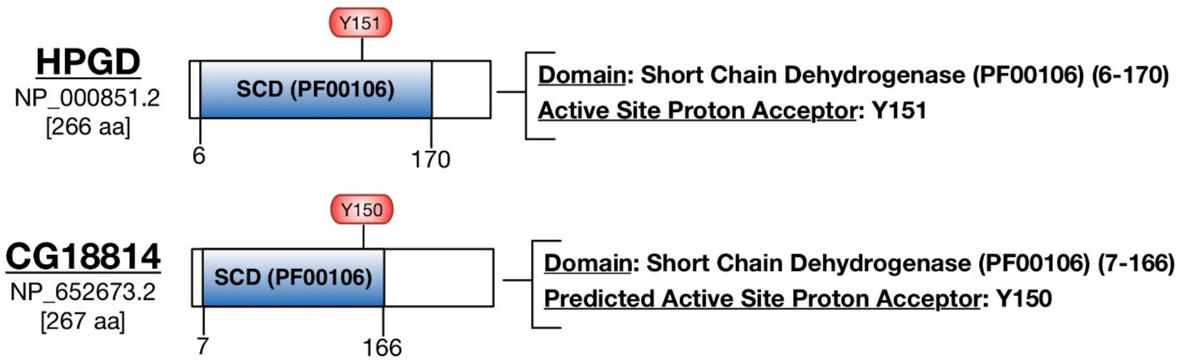
HPGD
NP_000851.2
[266 aa]
Y151
SCD (PF00106)
6
170
Domain: Short Chain Dehydrogenase (PF00106) (6-170)
Active Site Proton Acceptor: Y151
CG18814
NP_652673.2
[267 aa]
Y150
SCD (PF00106)
7
166
Domain: Short Chain Dehydrogenase (PF00106) (7-166)
Predicted Active Site Proton Acceptor: Y150

B.

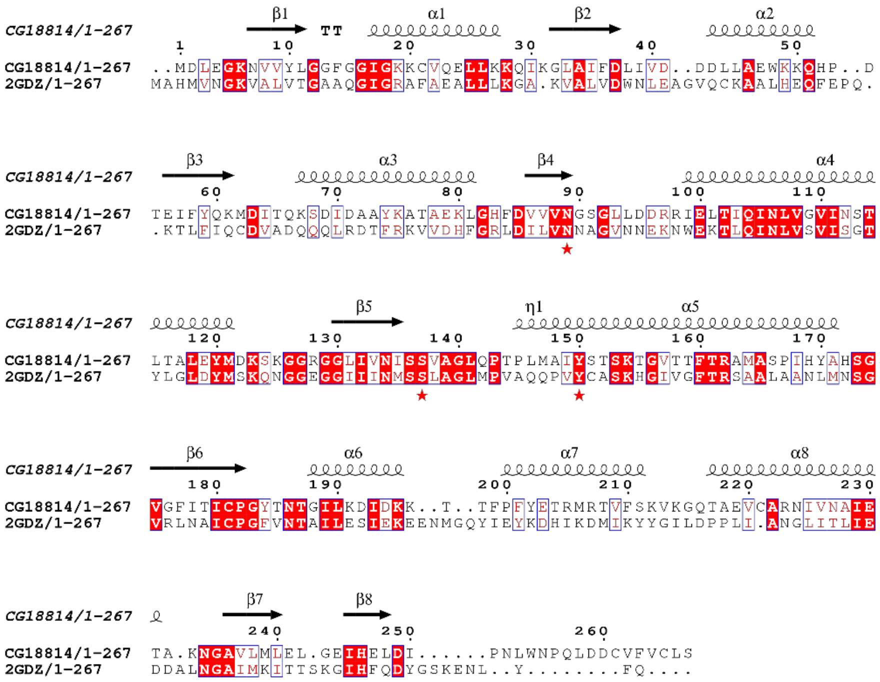
CG18814/1-267
β1 TT α1 β2 α2
CG18814/1-267 ..MDLEGKNVVYLGGFGGIGKKCVQELLKKQIKGLAIFDLIVD..DDLLAEWKKQHP..D
2GDZ/1-267 MAHMVNGKVALVTGAAQGIGRAFAEALLLKGA.KVALVDWNLEAGVQCKAALHEQFEPQ.
β3 α3 β4 α4
CG18814/1-267 TEIFYQKMDITQKSDIDAAYKATAEKLGHFDVVVNGSGLLDDRRIELTIQINLVGVINST
2GDZ/1-267 .KTLFIQCDVADQQQLRDTFRKVVDHFGRLDILVNNAGVNNEKNWEKTLQINLVSVISGT
β5 η1 α5
CG18814/1-267 LTALEYMDKSKGGRGGLIVNISSVAGLQPTPLMAIYSTSKTGVTTFTRAMASPIHYAHSG
2GDZ/1-267 YLGLDYMSKQNGGEGGIIINMSSLAGLMPVAQQPVYCASKHGIVGFTRSAALAANLMNSG
β6 α6 α7 α8
CG18814/1-267 VGFITICPGYTNTGILKDIDKK..T..TFPFYETRMRTVFSKVKGQTAEVCARNIVNAIE
2GDZ/1-267 VRLNAICPGFVNTAILESIEKEENMGQYIEYKDHIKDMIKYYGILDPPLI.ANGLITLIE
β7 β8
CG18814/1-267 TA.KNGAVLMLEL.GEIHELDI......PNLWNPQLDDCVFVCLS
2GDZ/1-267 DDALNGAIMKITTSKGIHFQDYGSKENL..Y........FQ....

C.

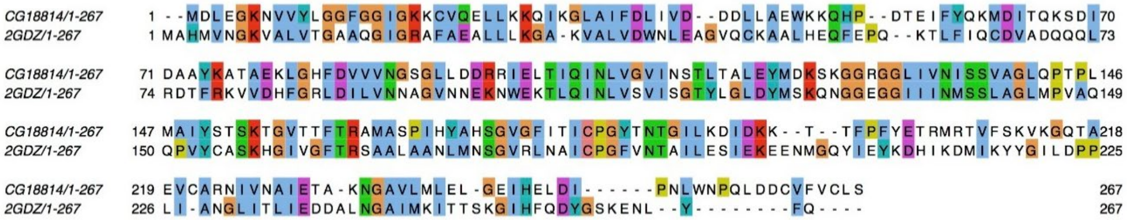
CG18814/1-267 1 --MDLEGKNVVYLGGFGGIGKKCVQELLKKQIKGLAIFDLIVD--DDLLAEWKKQHP--DTEIFYQKMDITQKSDI 70
2GDZ/1-267 1 MAHMVNGKVALVTGAAQGIGRAFAEALLLKGA-KVALVDWNLEAGVQCKAALHEQFEPQ--KTLFIQCDVADQQQL 73
CG18814/1-267 71 DAAYKATAEKLGHFDVVVNGSGLLDDRRIELTIQINLVGVINSTLTALEYMDKSKGGRGGLIVNISSVAGLQPTPL 146
2GDZ/1-267 74 RDTFRKVVDHFGRLDILVNNAGVNNEKNWEKTLQINLVSVISGTYLGLDYMSKQNGGEGGIIINMSSLAGLMPVAQ 149
CG18814/1-267 147 MAIYSTSKTGVTTFTRAMASPIHYAHSGVGFITICPGYTNTGILKDIDKK--T--TFPFYETRMRTVFSKVKGQTA 218
2GDZ/1-267 150 QPVYCASKHGIVGFTRSAALAANLMNSGVRLNAICPGFVNTAILESIEKEENMGQYIEYKDHIKDMIKYYGILDPP 225
CG18814/1-267 219 EVCARNIVNAIETA-KNGAVLMLEL-GEIHELDI------PNLWNPQLDDCVFVCLS 267
2GDZ/1-267 226 LI-ANGLITLIEDDALNGAIMKITTSKGIHFQDYGSKENL--Y--------FQ---- 267

D.

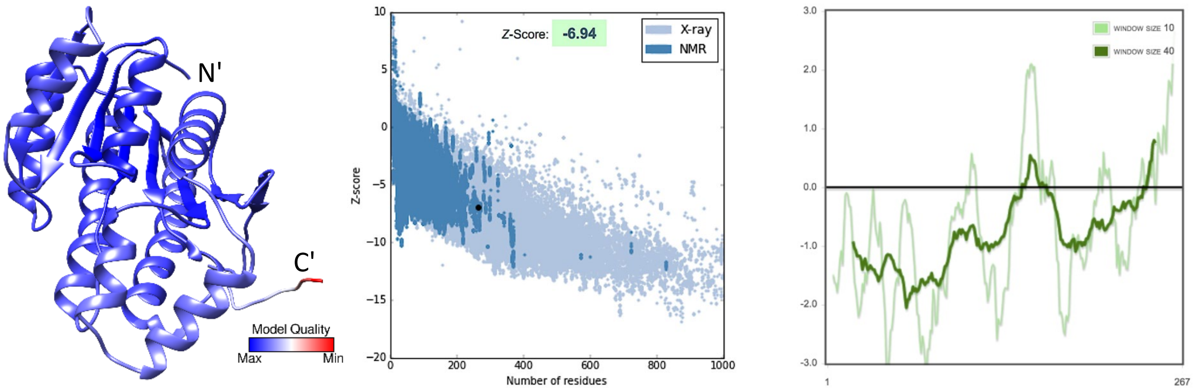

E.

**HPGD Structure**	***D. melanogaster* Model**	**Superimposed**
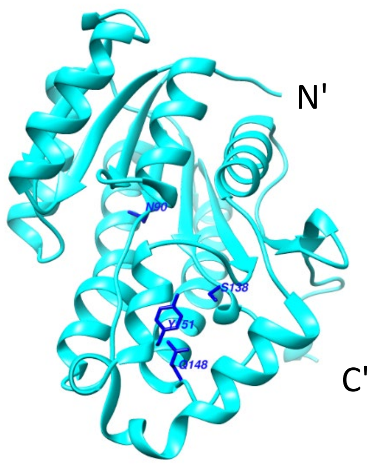	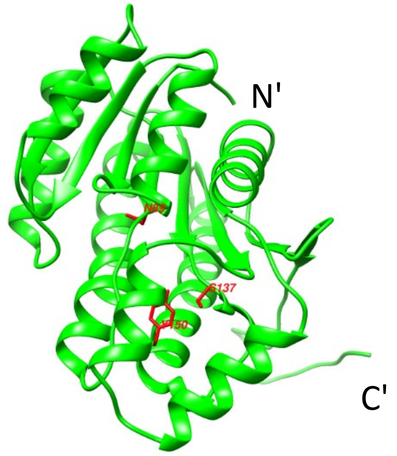	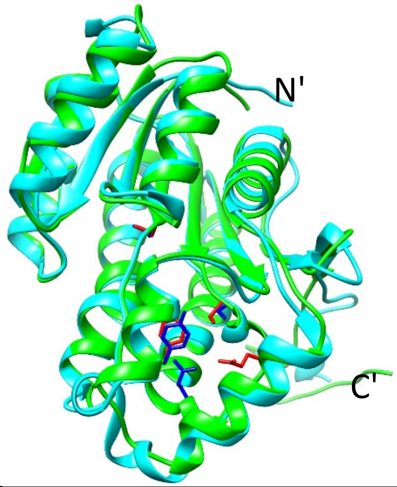

F.	Length (AA)	Domain Architecture (Pfam, range)	Functional Residues (aligned matches in *D. melanogaster*)	Sequence ID%	Structural Overlap (RMSD)
15-hydroxyprostaglandin dehydrogenase NAD(+) (HPGD, NP_000851.2, PDB: 2GDZ)	266	Short chain dehydrogenase domain (PF00106) 6-170	Y151	26% ID 47% SIM	0.715 Å
Uncharacterized protein (CG18814, NP_652673.2)	267	Short chain dehydrogenase domain (PF00106) 7-166	Y150		

S12 Fig. Sequence and structural details of the modeled fly HPGD candidate. A. Domain architecture of HPGD and CG18814 and known/predicted functional residues B. Pairwise alignment of CG18814 and 2GDZ generated from structural superposition showing shared secondary structure elements and known/predicted functional residues (marked with red asterisks) C. Pairwise alignment of CG18814 and 2GDZ generated from structural superposition with conserved residues highlighted using the physiochemical color scheme (CLUSTALX) D. Validation of the CG18814 model: ProQ2 quality score mapped to a 3D model of CG18814 (left); ProSA global quality score ranking (middle) and per-residue quality graph (right) E. HPGD (2GDZ, cyan-blue) superimposed on the predicted structure of CG18814 (green-red) with potential matches for conserved functional residues highlighted F. Summary of features shared by HPGD and potential *D. melanogaster* ortholog CG18814.
