# Supplementary material for: A combined computational strategy of sequence and structural analysis predicts the existence of a functional eicosanoid pathway in Drosophila melanogaster
Source: PLoS One. 2019 Feb 12;14(2):e0211897. doi: 10.1371/journal.pone.0211897 (PMC6372189; doi:10.1371/journal.pone.0211897)
Supplement: S13 Fig — A. Domain architecture of TBXAS1 and CG3616 and known/predicted functional residues B. Pairwise alignment of CG3616 and the modeled human TBXAS1 generated from structural superposition showing shared secondary structure elements and known/predicted functional residues (marked with a red asterisk) C. Pairwise alignment of CG3616 and the modeled human TBXAS1 generated from structural superposition with conserved residues highlighted using the physiochemical color scheme (CLUSTALX) D. Validation of the CG3616 model: ProQ2 quality score mapped to a 3D model of CG3616 (left); ProSA global quality score ranking (middle) and per-residue quality graph (right) E. TBXAS1 (model, cyan-blue) superimposed on the predicted structure of CG3616 (green-red) F. Summary of features shared by TBXAS1 and potential D. melanogaster ortholog CG3616. (PDF) [file pone.0211897.s013.pdf]

A.

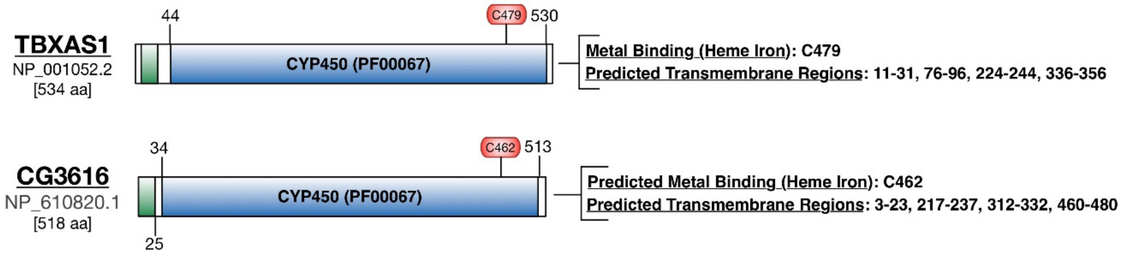
TBXAS1
NP_001052.2
[534 aa]
44
C479
530
CYP450 (PF00067)
Metal Binding (Heme Iron): C479
Predicted Transmembrane Regions: 11-31, 76-96, 224-244, 336-356
CG3616
NP_610820.1
[518 aa]
34
C462
513
25
CYP450 (PF00067)
Predicted Metal Binding (Heme Iron): C462
Predicted Transmembrane Regions: 3-23, 217-237, 312-332, 460-480

B.

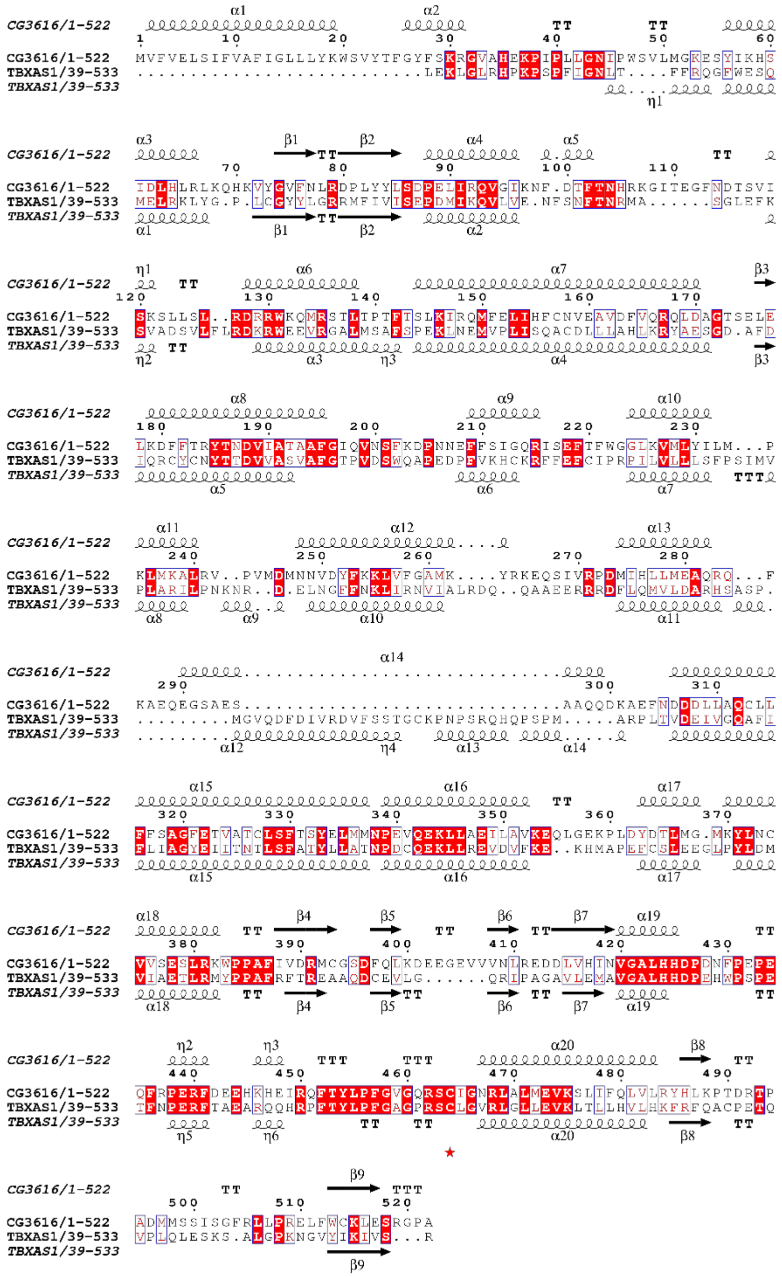
CG3616/1-522
TBXAS1/39-533

C.

```
CG3616/1-522     1 MVFVELSIFVAFIGLLLYKWSVYTFGYFSKRGVAHEKPIPLLGNIPWSVLMGKESYIKHSIDLHLRLKQHKVY 73
TBXAS1/39-533   39 ---------------------------LEKLGLRHPKPSPFIGNLT----FFRQGFWESQMELRKLYG-P-LC 78

CG3616/1-522    74 GVFNLRDPLYYLSDPELIRQVGIKNF-DTFTNHRKGITEGFNDTSVISKSLLSL--RDRRWKQMRSTLTPTFT 143
TBXAS1/39-533   79 GYYLGRRMFIVISEPDMIKQVLVE-NFSNFTNRMA------SGLEFKSVADSVLFLRDKRWEEVRGALMSAFS 144

CG3616/1-522   144 SLKIRQMFELIHFCNVEAVDFVQRQLDAGTSELELKDFFTRYTNDVIATAAFGIQVNSFKDPNNEFFSIGQRI 216
TBXAS1/39-533  145 PEKLNEMVPLISQACDLLAHLKRYAESGD-AFDIQRCYCNYTTDVVASVAFGTPVDSWQAPEDPFVKHCKRF 216

CG3616/1-522   217 SEFTFWGGLKVMLYILM---PKLMKALRV--PVMDMNNVDYFKKLVFGAMK----YRKEQSIVRPDMIHLLME 280
TBXAS1/39-533  217 FEFCIPRPILVLLLSFPSIMVPLARILPNKNR--D-ELNGFFNKLIRNVIALRDQ--QAAEERRRDFLQMVLD 284

CG3616/1-522   281 AQRQ---FKAEQEGSAES------------------------------AAQQDKAEFNDDDLLAQCLLFFSAG 320
TBXAS1/39-533  285 ARHSASP----------MGVQDFDIVRDVFSSTGCKPNPSRQHQPSPM-----ARPLTVDEIVGQAFIFLIAG 342

CG3616/1-522   321 FETVATCLSFTSYELMMNPEVQEKLLAEILAVKEQLGEKPLDYDTLMG-MKYLNCVVSESLRKWPPAFIVDRM 392
TBXAS1/39-533  343 YEIITNTLSFATYLLATNPDCQEKLLREVDVFKE--KHMAPEFCSLEEGLPYLDMVIAETLRMYPPAFRFTRE 413

CG3616/1-522   393 CGSDFQLKDEEGEVVVNLREDDLVHINVGALHHDPDNFPEPEQFRPERFDEEHKHEIRQFTYLPFGVGQRSCI 465
TBXAS1/39-533  414 AAQDCEVLG------QRIPAGAVLEMAVGALHHDPEHWPSPETFNPERFTAEARQQHRPFTYLPFGAGPRSCL 480

CG3616/1-522   466 GNRLALMEVKSLIFQLVLRYHLKPTDRTPADMMSSISGFRLLPRELFWCKLESRGPA 522
TBXAS1/39-533  481 GVRLGLLEVKLTLLHVLHKFRFQACPETQVPLQLESKS-ALGPKNGVYIKIVS---R 533
```

D.

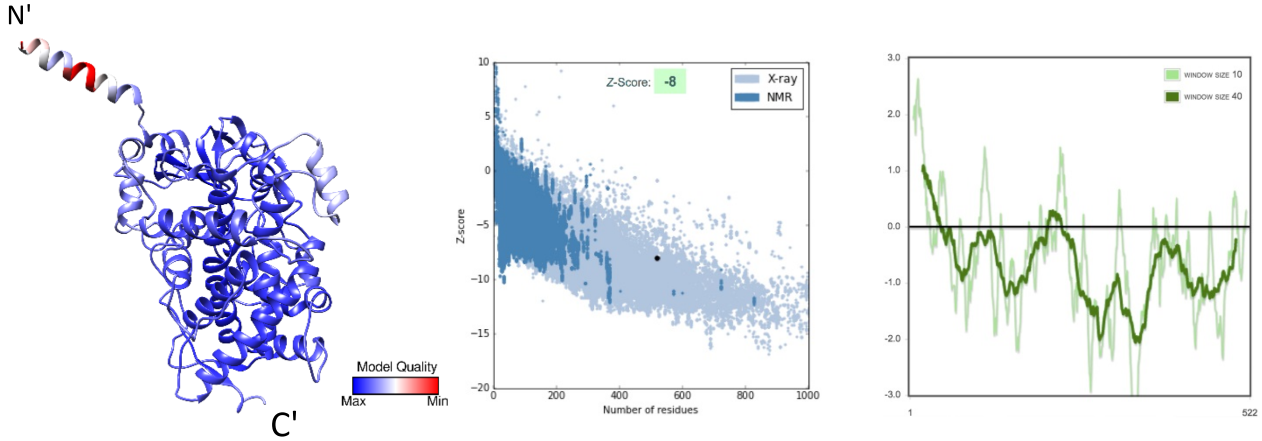

E.

TBXAS1 Model	*D. melanogaster* Model	Superimposed
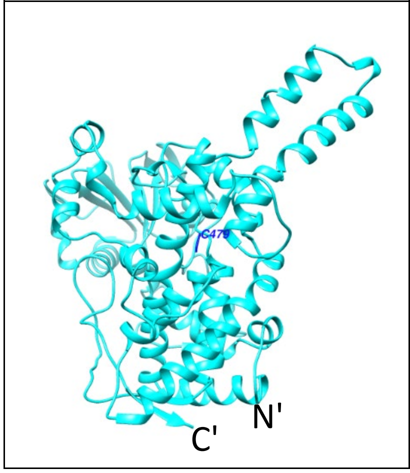	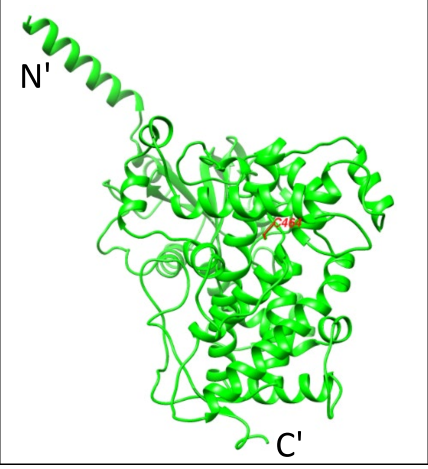	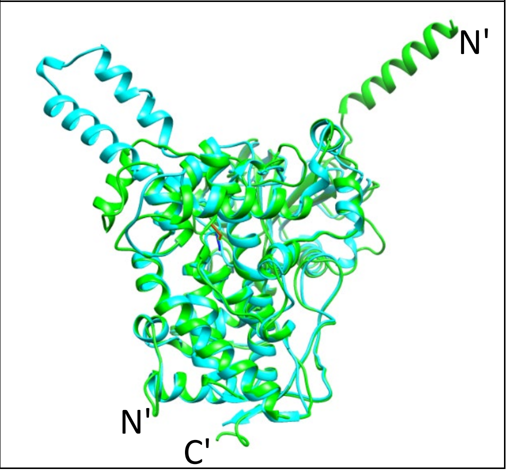

F.

<table>
<tr><th></th><th>Length (AA)</th><th>Domain Architecture (Pfam, range)</th><th>Functional Residues (aligned matches in D. melanogaster)</th><th>Sequence ID%</th><th>Structural Overlap (RMSD)</th></tr>
<tr><td>Thromboxane A synthase (TBXAS1, NP_001052.2, PDB: N/A (modeled))</td><td>534</td><td>Cytochrome P450 domain (PF00067) 44-530</td><td>C479</td><td rowspan="2">27% ID
45% SIM</td><td rowspan="2">0.699 Å</td></tr>
<tr><td>Cytochrome P450-9c1 (CG3616, NP_610820.1)</td><td>518</td><td>Cytochrome P450 domain (PF00067) 34-513</td><td>C462</td></tr>
</table>

S13 Fig. Sequence and structural details of the modeled fly TBXAS1 candidate. A. Domain architecture of TBXAS1 and CG3616 and known/predicted functional residues B. Pairwise alignment of CG3616 and the modeled human TBXAS1 generated from structural superposition showing shared secondary structure elements and known/predicted functional residues (marked with a red asterisk) C. Pairwise alignment of CG3616 and the modeled human TBXAS1 generated from structural superposition with conserved residues highlighted using the physiochemical color scheme (CLUSTALX) D. Validation of the CG3616 model: ProQ2 quality score mapped to a 3D model of CG3616 (left); ProSA global quality score ranking (middle) and per-residue quality graph (right) E. TBXAS1 (model, cyan-blue) superimposed on the predicted structure of CG3616 (green-red) F. Summary of features shared by TBXAS1 and potential *D. melanogaster* ortholog CG3616.
