# Supplementary material for: A combined computational strategy of sequence and structural analysis predicts the existence of a functional eicosanoid pathway in Drosophila melanogaster
Source: PLoS One. 2019 Feb 12;14(2):e0211897. doi: 10.1371/journal.pone.0211897 (PMC6372189; doi:10.1371/journal.pone.0211897)
Supplement: S14 Fig — A. Domain architecture of PTGS1 and CG4009 and known/predicted functional residues B. Pairwise alignment of CG4009 and 3N8V generated from structural superposition showing shared secondary structure elements and known/predicted functional residues (marked with red asterisk; the green asterisk marks the putative analog for Y384) C. Pairwise alignment of CG4009 and 3N8V generated from structural superposition with conserved residues highlighted using the physiochemical color scheme (CLUSTALX) D. Validation of the CG4009 model: ProQ2 quality score mapped to a 3D model of CG4009 (left); ProSA global quality score ranking (middle) and per-residue quality graph (right) E. PTGS1 (3N8V, cyan-blue) superimposed on the predicted structure of CG4009 (green-red) with potential matches for conserved functional residues highlighted F. Summary of features shared by PTGS1 and potential D. melanogaster ortholog CG4009. (PDF) [file pone.0211897.s014.pdf]

A.

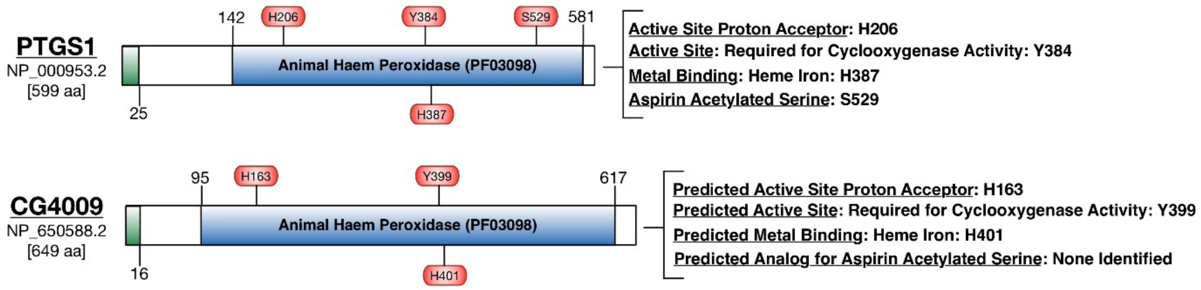
PTGS1
NP_000953.2
[599 aa]
142
H206
Y384
S529
581
Animal Haem Peroxidase (PF03098)
H387
25
Active Site Proton Acceptor: H206
Active Site: Required for Cyclooxygenase Activity: Y384
Metal Binding: Heme Iron: H387
Aspirin Acetylated Serine: S529
CG4009
NP_650588.2
[649 aa]
95
H163
Y399
617
Animal Haem Peroxidase (PF03098)
H401
16
Predicted Active Site Proton Acceptor: H163
Predicted Active Site: Required for Cyclooxygenase Activity: Y399
Predicted Metal Binding: Heme Iron: H401
Predicted Analog for Aspirin Acetylated Serine: None Identified

B.

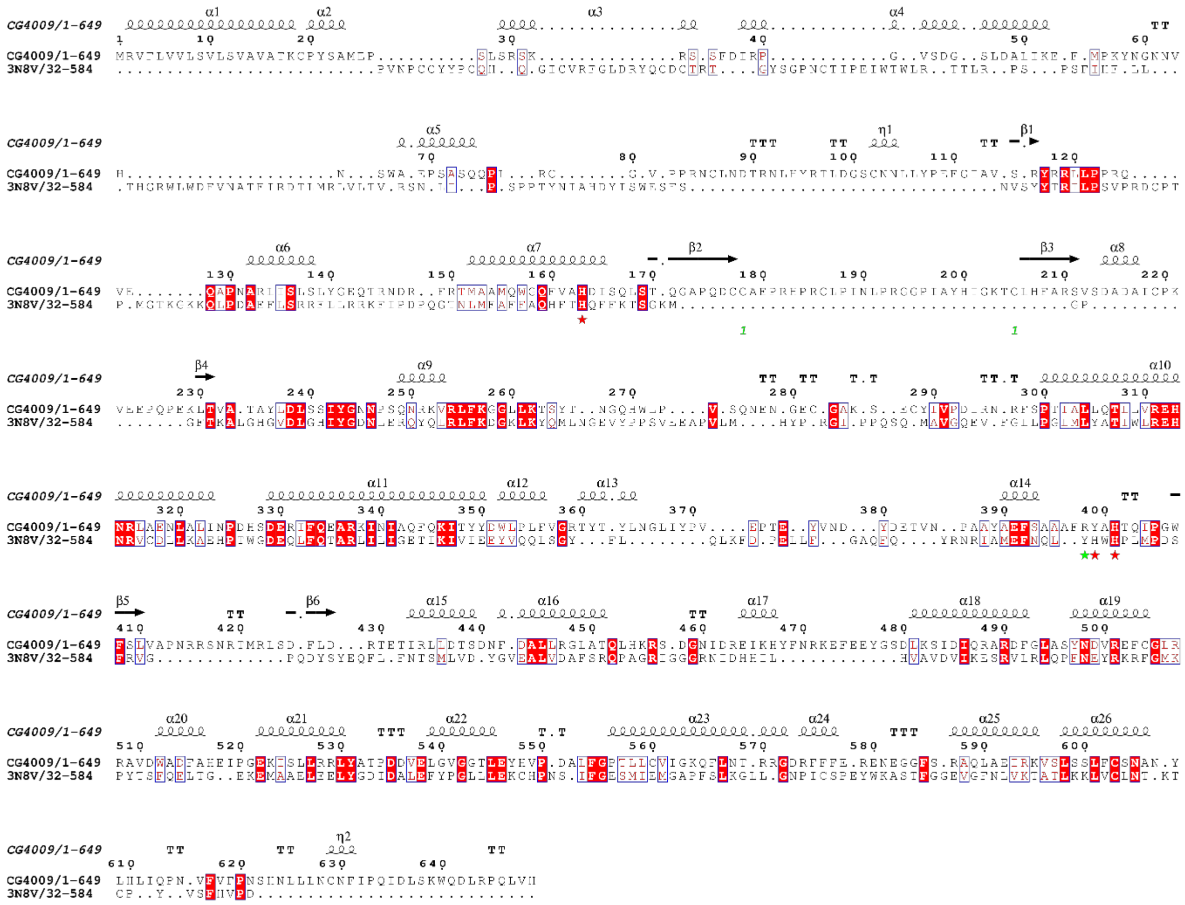
CG4009/1-649
3N8V/32-584

C.

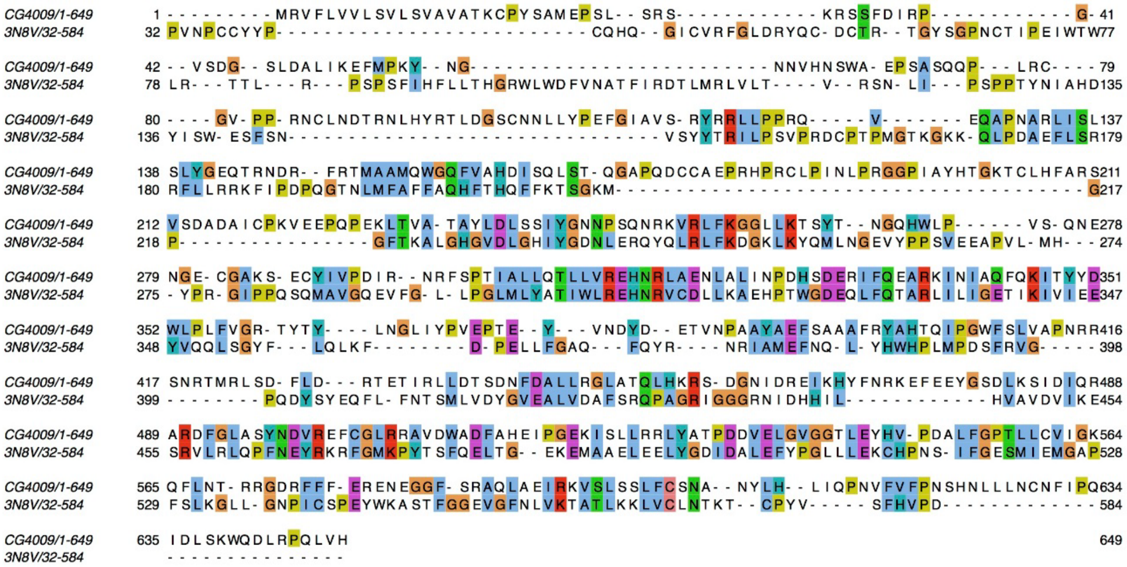

D.

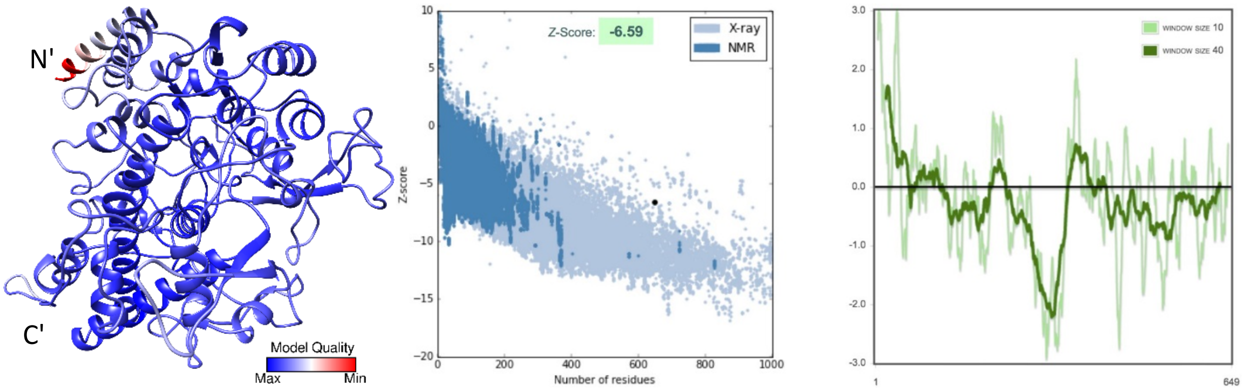

E.

PTGS1 Structure	*D. melanogaster* Model	Superimposed
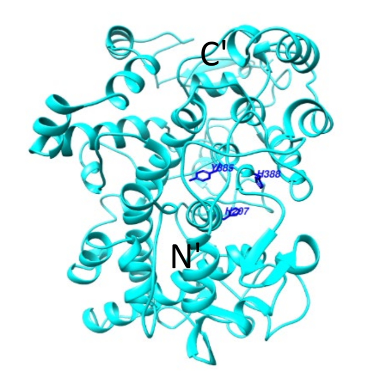	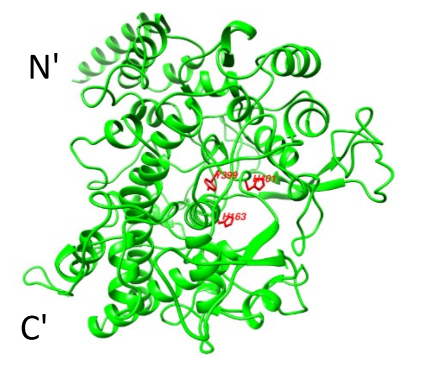	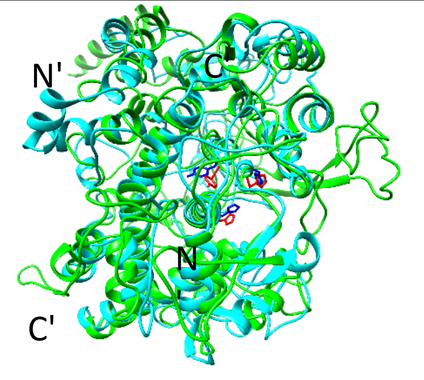

F.	Length (AA)	Domain Architecture (Pfam, range)	Functional Residues (aligned matches in *D. melanogaster*)	Sequence ID%	Structural Overlap (RMSD)
Cyclooxygenase 1 (PTGS1, NP_000953.2, PDB: 3N8V)	599	Animal heme peroxidase domain (PF03098) 142-581	H207, Y385 and H388	14% ID 26% SIM	2.660 Å
Uncharacterized protein CG4009, NP_650588.2)	649	Animal heme peroxidase domain (PF03098) 95-617	H163, Y399, and H401		

S14 Fig. Sequence and structural details of the modeled fly PTGS1 candidate. A. Domain architecture of PTGS1 and CG4009 and known/predicted functional residues B. Pairwise alignment of CG4009 and 3N8V generated from structural superposition showing shared secondary structure elements and known/predicted functional residues (marked with red asterisk; the green asterisk marks the putative analog for Y384) C. Pairwise alignment of CG4009 and 3N8V generated from structural superposition with conserved residues highlighted using the physiochemical color scheme (CLUSTALX) D. Validation of the CG4009 model: ProQ2 quality score mapped to a 3D model of CG4009 (left); ProSA global quality score ranking (middle) and per-residue quality graph (right) E. PTGS1 (3N8V, cyan-blue) superimposed on the predicted structure of CG4009 (green-red) with potential matches for conserved functional residues highlighted F. Summary of features shared by PTGS1 and potential *D. melanogaster* ortholog CG4009.
