# Supplementary material for: A combined computational strategy of sequence and structural analysis predicts the existence of a functional eicosanoid pathway in Drosophila melanogaster
Source: PLoS One. 2019 Feb 12;14(2):e0211897. doi: 10.1371/journal.pone.0211897 (PMC6372189; doi:10.1371/journal.pone.0211897)
Supplement: S15 Fig — A. Domain architecture of PTGDS and CG33126 and known/predicted functional residues B. Pairwise alignment of CG33126 and 5WY9 generated from structural superposition showing shared secondary structure elements and known/predicted functional residues (marked with a red asterisk; the green asterisk denotes the putative analog for C65) C. Pairwise alignment of CG33126 and 5WY9 generated from structural superposition with conserved residues highlighted using the physiochemical color scheme (CLUSTALX) D. Validation of the CG33126 model: ProQ2 quality score mapped to a 3D model of CG33126 (left); ProSA global quality score ranking (middle) and per-residue quality graph (right) E. PTGDS (5WY9, cyan-blue) superimposed on the predicted structure of CG33126 (green-red) with potential matches for conserved functional residues highlighted F. Summary of features shared by PTGDS and potential D. melanogaster ortholog CG33126. (PDF) [file pone.0211897.s015.pdf]

A.

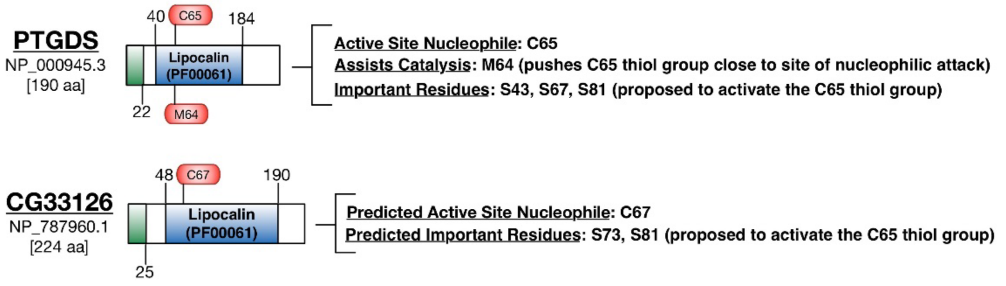
PTGDS
NP_000945.3
[190 aa]
40
C65
184
Lipocalin (PF00061)
22
M64
Active Site Nucleophile: C65
Assists Catalysis: M64 (pushes C65 thiol group close to site of nucleophilic attack)
Important Residues: S43, S67, S81 (proposed to activate the C65 thiol group)
CG33126
NP_787960.1
[224 aa]
48
C67
190
Lipocalin (PF00061)
25
Predicted Active Site Nucleophile: C67
Predicted Important Residues: S73, S81 (proposed to activate the C65 thiol group)

B.

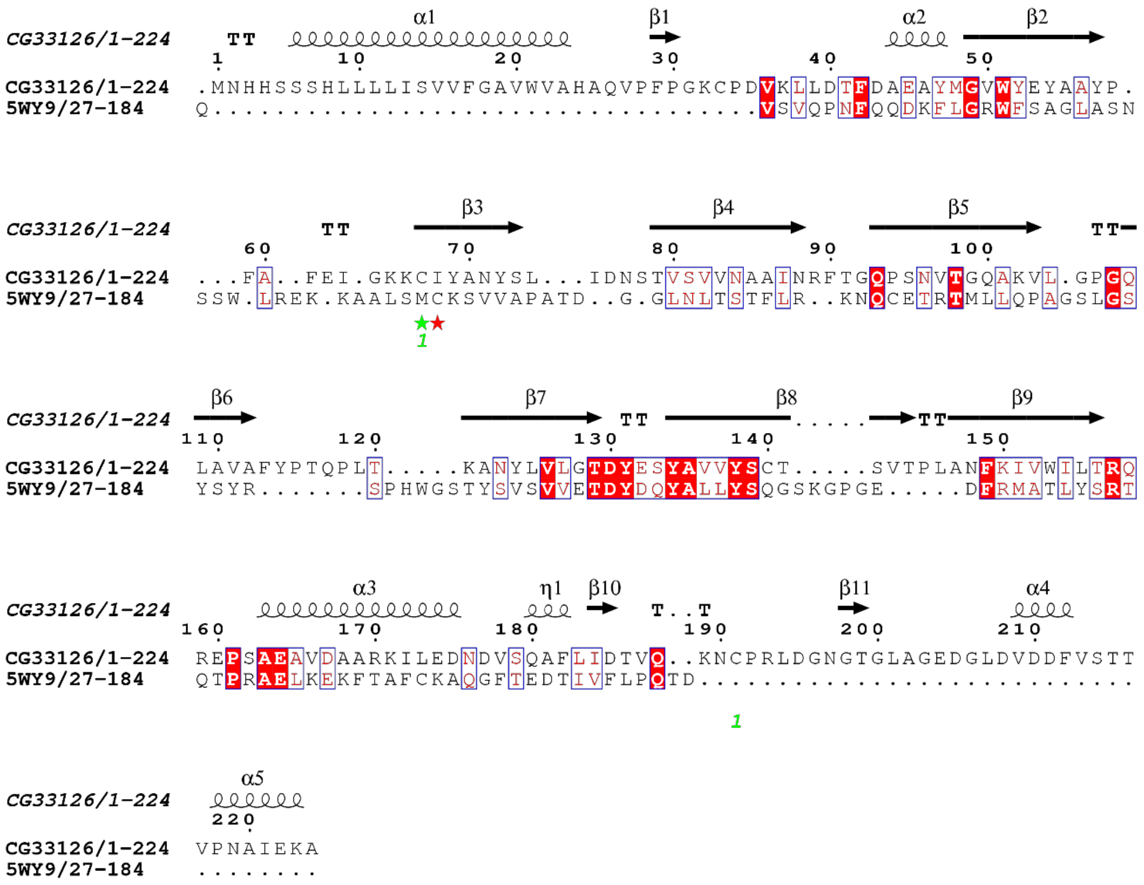
CG33126/1-224
CG33126/1-224 .MNHHSSSHLLLLISVVFGAVWVAHAQVPFPGKCPDVKLLDTFDAEAYMGVWYEYAAYP.
5WY9/27-184 Q...............................VSVQPNFQQDKFLGRWFSAGLASN
CG33126/1-224 ...FA..FEI.GKKCIYANYSL...IDNSTVSVVNAAINRFTGQPSNVTGQAKVL.GPGQ
5WY9/27-184 SSW.LREK.KAALSMCKSVVAPATD..G.GLNLTSTFLR..KNQCETRTMLLQPAGSLGS
CG33126/1-224 LAVAFYPTQPLT.....KANYLVLGTDYESYAVVYSCT.....SVTPLANFKIVWILTRQ
5WY9/27-184 YSYR.......SPHWGSTYSVSVVETDYDQYALLYSQGSKGPGE.....DFRMATLYSRT
CG33126/1-224 REPSAEAVDAARKILEDNDVSQAFLIDTVQ..KNCPRLDGNGTGLAGEDGLDVDDFVSTT
5WY9/27-184 QTPRAELKEKFTAFCKAQGFTEDTIVFLPQTD..........................
CG33126/1-224 VPNAIEKA
5WY9/27-184 ....

C.

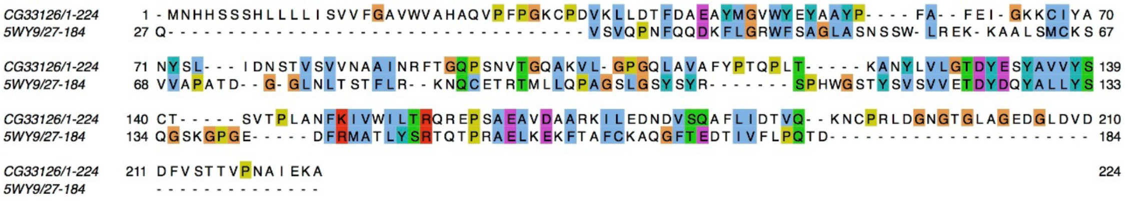
CG33126/1-224 1 - MNHHSSSHLLLLISVVFGAVWVAHAQVPFPGKCPDVKLLDTFDAEAYMGVWYEYAAYP- - - - FA- - FEI- GKKCIYA 70
5WY9/27-184 27 Q- VSVQPNFQQDKFLGRWFSAGLASNSSW- LREK- KAALSMCKS 67
CG33126/1-224 71 NYSL- - - IDNSTVSVVNAAINRFTGQPSNVTGQAKVL- GPGQLAVAFYPTQPLT- - - - - KANYLVLGTDYESYAVVYS 139
5WY9/27-184 68 VVAPATD- - G- GLNLTSTFLR- - KNQCETRTMLLQPAGSLGSYSYR- - - - - - - SPHWGSTYSVSVVETDYDQYALLYS 133
CG33126/1-224 140 CT- - - - - SVTPLANFKIVWILTRQREPSAEAVDAARKILEDNDVSQAFLIDTVQ- - KNCPRLDGNGTGLAGEDGLDVD 210
5WY9/27-184 134 QGSKGPGE- - - - - DFRMATLYSRTQTPRAELKEKFTAFCKAQGFTEDTIVFLPQTD- 184
CG33126/1-224 211 DFVSTTVPNAIEKA 224
5WY9/27-184 - - - - - - - - - - - - - -

D.

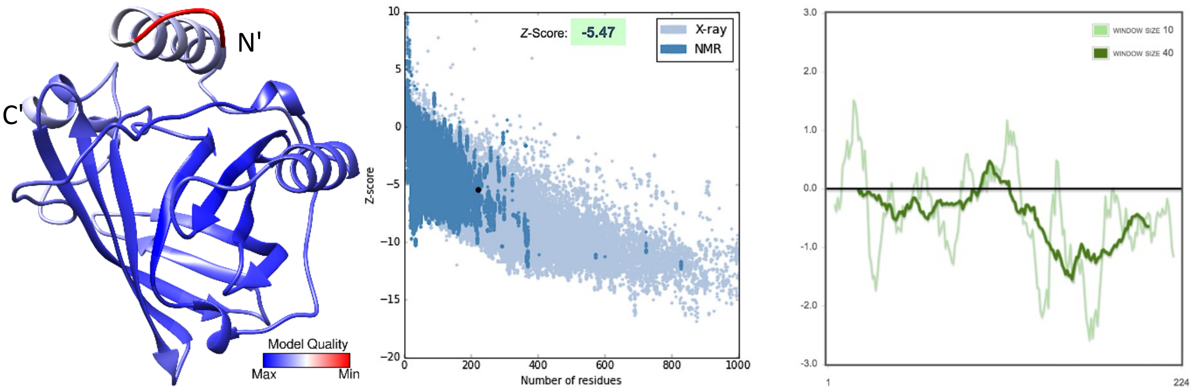

E.

**PTGDS Structure**	***D. melanogaster* Model**	**Superimposed**
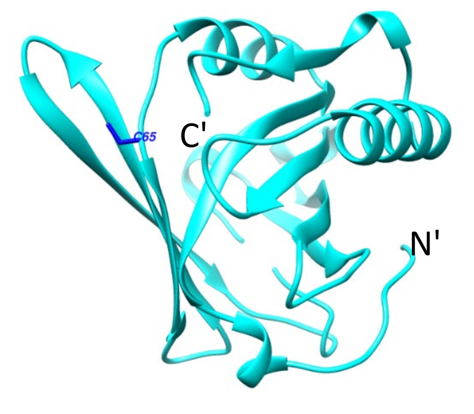	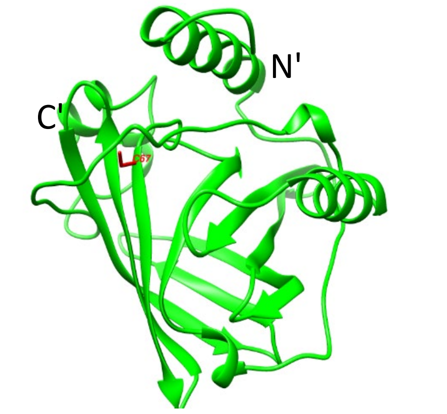	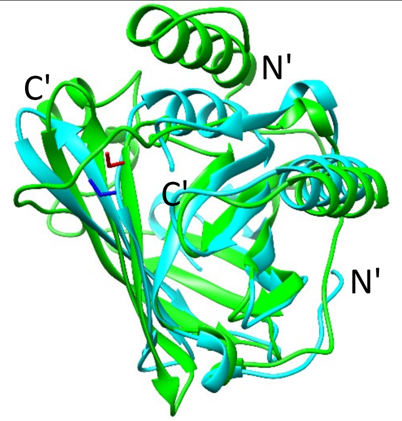

F.

	Length (AA)	Domain Architecture (Pfam, range)	Functional Residues (aligned matches in *D. melanogaster*)	Sequence ID%	Structural Overlap (RMSD)
Prostaglandin-D-synthase (PTGDS, NP_000945.3, PDB: 5WY9)	190	Lipocalin domain (PF00061) 40-184	C65	12% ID<br>31% SIM	2.084 Å
Neural Lazarillo (CG33126, NP_787960.1)	224	Lipocalin domain (PF00061) 48-190	C67		

S15 Fig. Sequence and structural details of the modeled fly PTGDS candidate. A. Domain architecture of PTGDS and CG33126 and known/predicted functional residues B. Pairwise alignment of CG33126 and 5WY9 generated from structural superposition showing shared secondary structure elements and known/predicted functional residues (marked with a red asterisk; the green asterisk denotes the putative analog for C65) C. Pairwise alignment of CG33126 and 5WY9 generated from structural superposition with conserved residues highlighted using the physiochemical color scheme (CLUSTALX) D. Validation of the CG33126 model: ProQ2 quality score mapped to a 3D model of CG33126 (left); ProSA global quality score ranking (middle) and per-residue quality graph (right) E. PTGDS (5WY9, cyan-blue) superimposed on the predicted structure of CG33126 (green-red) with potential matches for conserved functional residues highlighted F. Summary of features shared by PTGDS and potential *D. melanogaster* ortholog CG33126.
