# Supplementary material for: A combined computational strategy of sequence and structural analysis predicts the existence of a functional eicosanoid pathway in Drosophila melanogaster
Source: PLoS One. 2019 Feb 12;14(2):e0211897. doi: 10.1371/journal.pone.0211897 (PMC6372189; doi:10.1371/journal.pone.0211897)
Supplement: S16 Fig — A. Domain architecture of PTG1S and CG3466 and known/predicted functional residues B. Pairwise alignment of CG34666 and 2IAG generated from structural superposition showing shared secondary structure elements and known/predicted functional residues (marked with a red asterisk) C. Pairwise alignment of CG3466 and 2IAG generated from structural superposition with conserved residues highlighted using the physiochemical color scheme (CLUSTALX) D. Validation of the CG3466 model: ProQ2 quality score mapped to a 3D model of CG3466 (left); ProSA global quality score ranking (middle) and per-residue quality graph (right) E. PTG1S (2IAG, cyan-blue) superimposed on the predicted structure of CG3466 (green-red) with potential matches for conserved functional residues highlighted F. Summary of features shared by PTG1S and potential D. melanogaster ortholog CG3466. (PDF) [file pone.0211897.s016.pdf]

A.

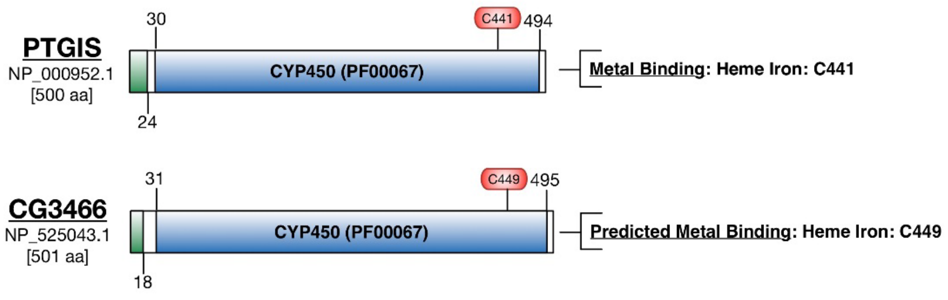
PTGIS
NP_000952.1
[500 aa]
30
24
C441
494
CYP450 (PF00067)
Metal Binding: Heme Iron: C441
CG3466
NP_525043.1
[501 aa]
31
18
C449
495
CYP450 (PF00067)
Predicted Metal Binding: Heme Iron: C449

B.

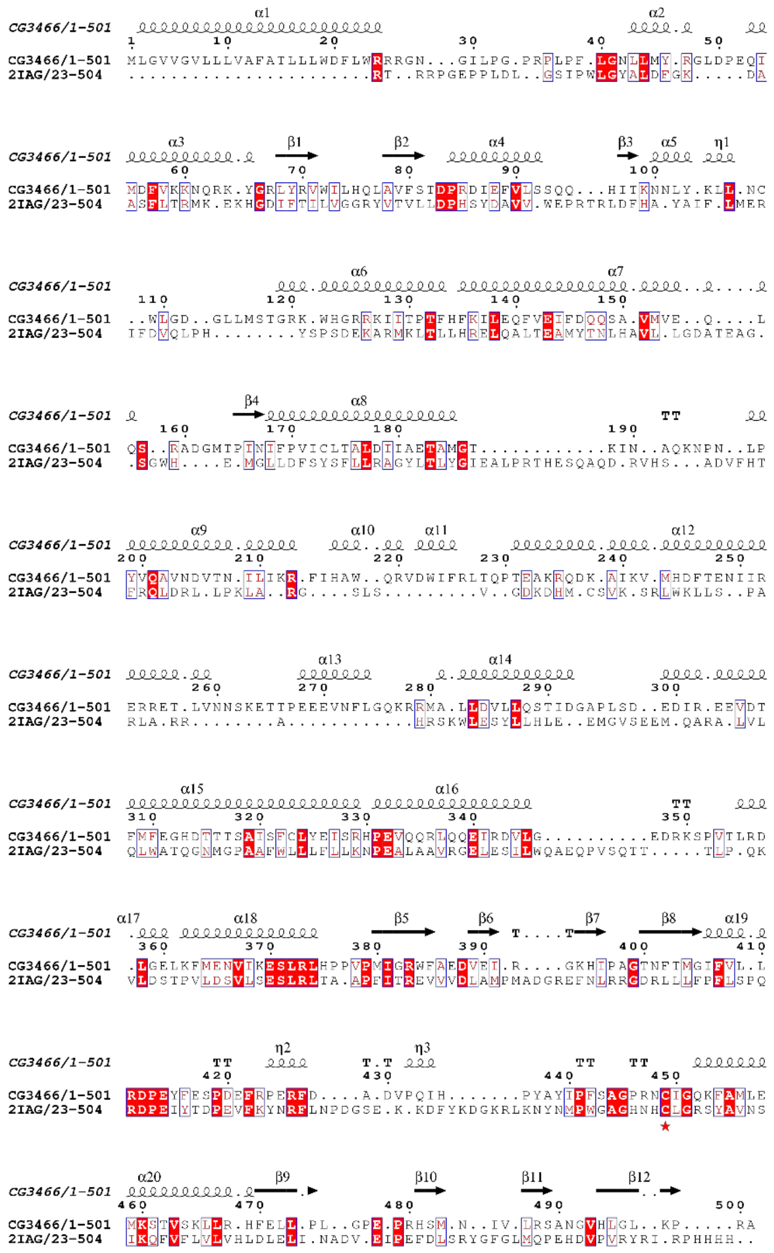
CG3466/1-501
2IAG/23-504

C.

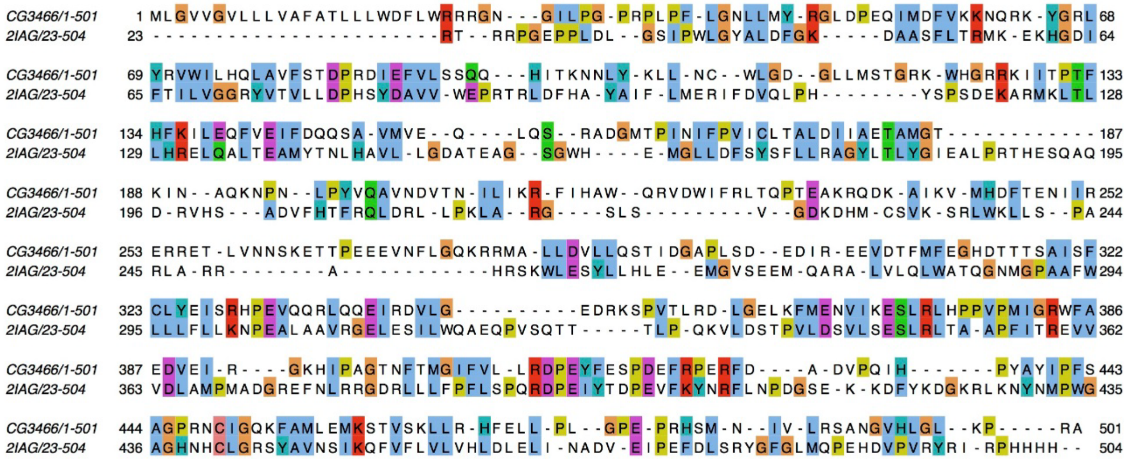

D.

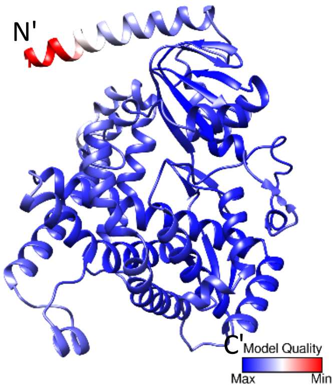

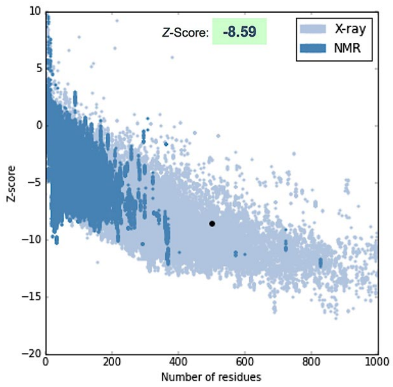

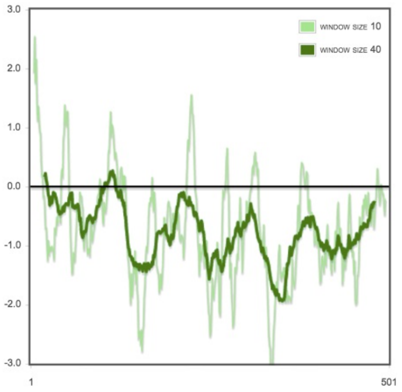

E.

PTGIS Structure	*D. melanogaster* Model	Superimposed
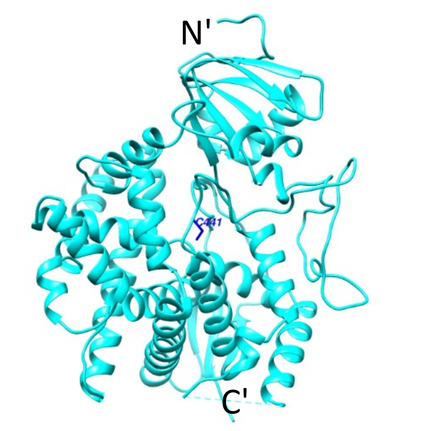	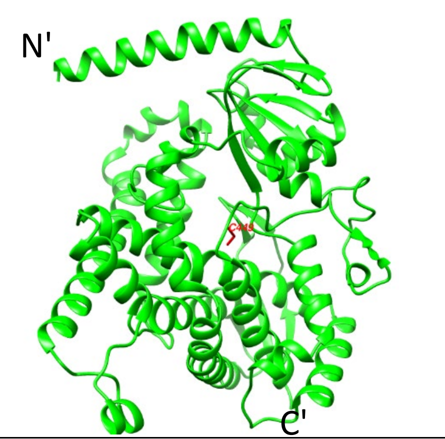	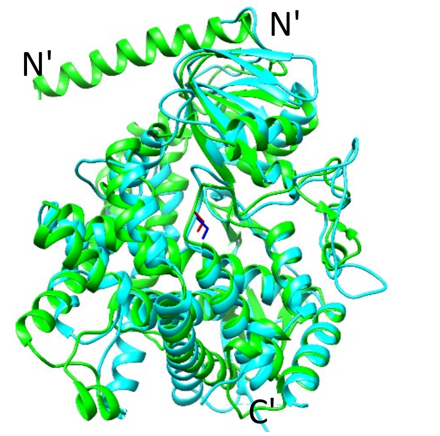

F.	Length (AA)	Domain Architecture (Pfam, range)	Functional Residues (aligned matches in *D. melanogaster*)	Sequence ID%	Structural Overlap (RMSD)
Prostacyclin synthase (PTGIS, NP_000952.1, PDB: 2IAG)	500	Cytochrome p450 family (PF00067) 30-494	C441	14% ID 30% SIM	1.213 Å
Cytochrome p450-4d2 (CG3466, NP_525043.1)	501	Cytochrome p450 family (PF00067) 31-495	C449		

S16 Fig. Sequence and structural details of the modeled fly PTGIS candidate. A. Domain architecture of PTG1S and CG3466 and known/predicted functional residues B. Pairwise alignment of CG34666 and 2IAG generated from structural superposition showing shared secondary structure elements and known/predicted functional residues (marked with a red asterisk) C. Pairwise alignment of CG3466 and 2IAG generated from structural superposition with conserved residues highlighted using the physiochemical color scheme (CLUSTALX) D. Validation of the CG3466 model: ProQ2 quality score mapped to a 3D model of CG3466 (left); ProSA global quality score ranking (middle) and per-residue quality graph (right) E. PTG1S (2IAG, cyan-blue) superimposed on the predicted structure of CG3466 (green-red) with potential matches for conserved functional residues highlighted F. Summary of features shared by PTG1S and potential *D. melanogaster* ortholog CG3466.
