# Supplementary material for: A combined computational strategy of sequence and structural analysis predicts the existence of a functional eicosanoid pathway in Drosophila melanogaster
Source: PLoS One. 2019 Feb 12;14(2):e0211897. doi: 10.1371/journal.pone.0211897 (PMC6372189; doi:10.1371/journal.pone.0211897)
Supplement: S17 Fig — A. Domain architecture of LTCS4 and CG33178 and known/predicted functional residues B. Pairwise alignment of CG33178 and 2PNO generated from structural superposition showing shared secondary structure elements and known/predicted functional residues (marked with red asterisks; the green asterisk denotes the analog for R31) C. Pairwise alignment of CG33178 and 2PNO generated from structural superposition with conserved residues highlighted using the physiochemical color scheme (CLUSTALX) D. Validation of the CG33178 model: ProQ2 quality score mapped to a 3D model of CG33178 (left); ProSA global quality score ranking (middle) and per-residue quality graph (right) E. LTCS4 (2PNO, cyan-blue) superimposed on the predicted structure of CG33178 (green-red) with potential matches for conserved functional residues highlighted F. Summary of features shared by LTCS4 and potential D. melanogaster ortholog CG33178. (PDF) [file pone.0211897.s017.pdf]

A.

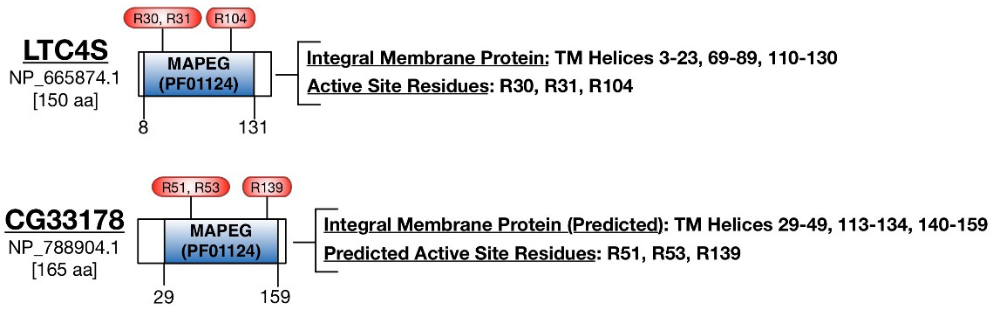
R30, R31
R104
LTC4S
NP_665874.1
[150 aa]
MAPEG (PF01124)
8
131
Integral Membrane Protein: TM Helices 3-23, 69-89, 110-130
Active Site Residues: R30, R31, R104
R51, R53
R139
CG33178
NP_788904.1
[165 aa]
MAPEG (PF01124)
29
159
Integral Membrane Protein (Predicted): TM Helices 29-49, 113-134, 140-159
Predicted Active Site Residues: R51, R53, R139

B.

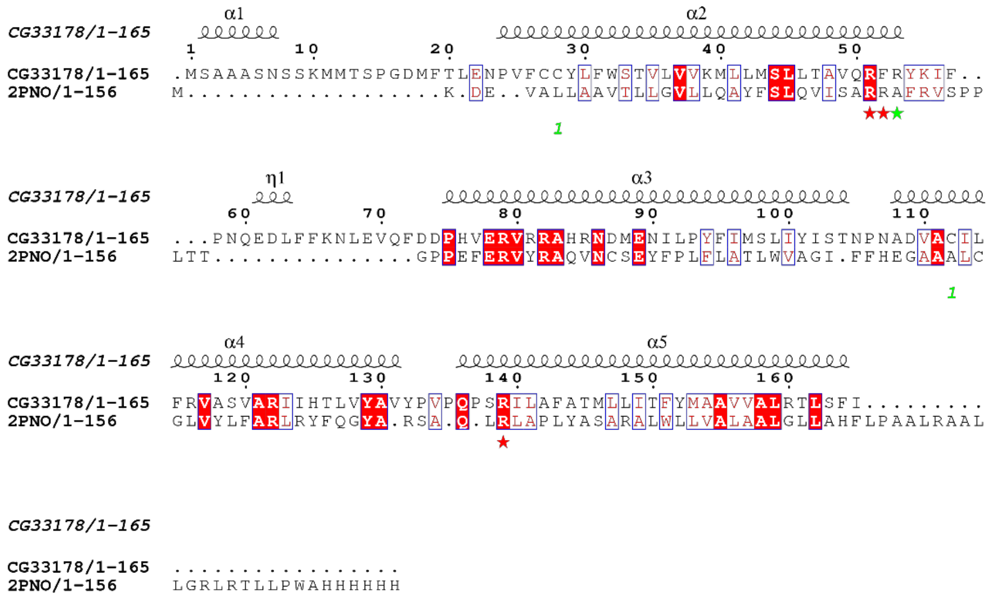
CG33178/1-165
α1
α2
CG33178/1-165 .MSAAASNSSKMMTSPGDMFTLENPVFCCYLFWSTVLVVKMLLMSLLTAVQRFRYKIF..
2PNO/1-156 M.................K.DE..VALLAAVTLLGVLLQAYFSLQVISARRAFRVSPP
η1
α3
CG33178/1-165 ...PNQEDLFFKNLEVQFDDPHVERVRRAHRNDMENILPYFIMSLIYISTNPNADVACIL
2PNO/1-156 LTT..............GPPEFERVYRAQVNCSEYFPLFLATLWVAGI.FFHEGAAALC
α4
α5
CG33178/1-165 FRVASVARIIHTLVYAVYPVPQPSRILAFATMLLITFYMAAVVALRTLSFI.........
2PNO/1-156 GLVYLFARLRYFQGYA.RSA.Q.LRLAPLYASARALWLLVALAALGLLAHFLPAALRAAL
CG33178/1-165 ....
2PNO/1-156 LGRLRTLLPWAHHHHHH

C.

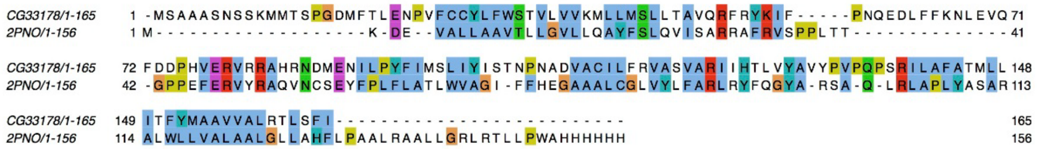
CG33178/1-165
2PNO/1-156

D.

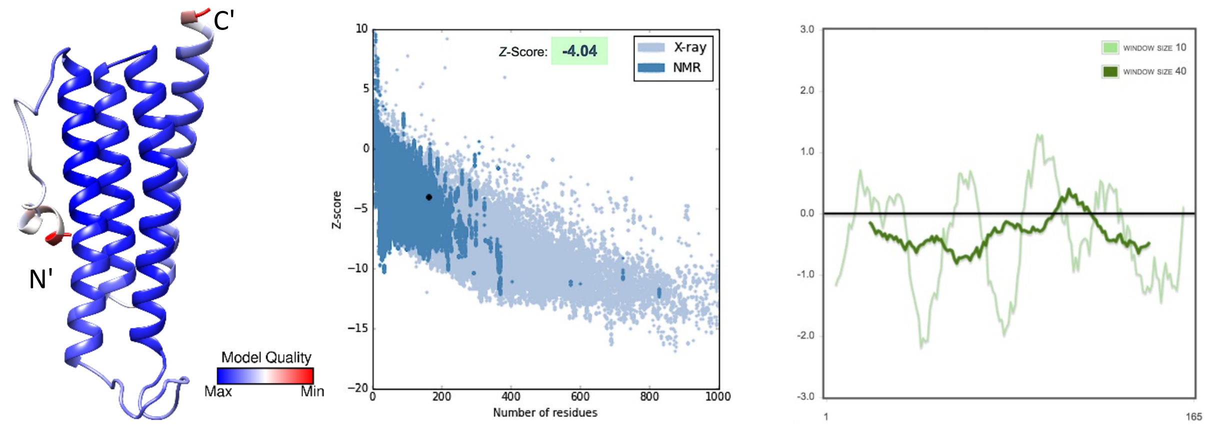

E.

LTC4S Structure	*D. melanogaster* Model	Superimposed
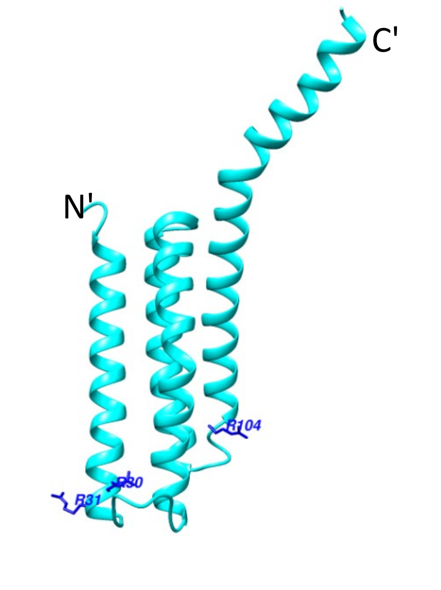	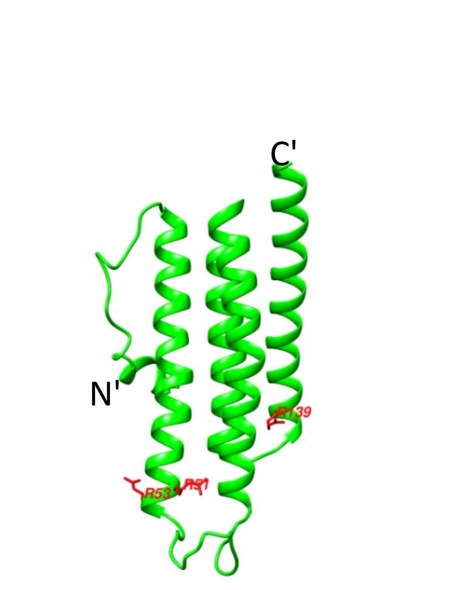	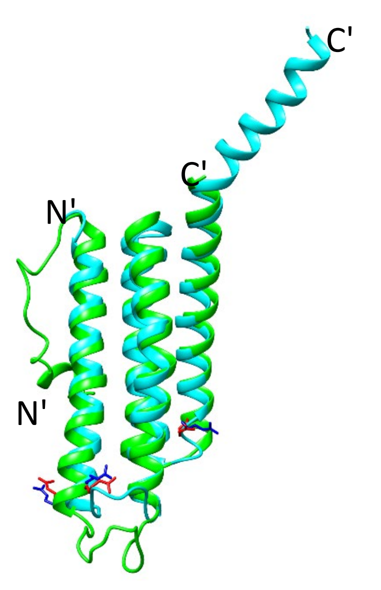

F.	Length (AA)	Domain Architecture (Pfam, range)	Functional Residues (aligned matches in *D. melanogaster*)	Sequence ID%	Structural Overlap (RMSD)
Leukotriene C4 synthase (LTC4S, NP_665874.1, PDB: 2PNO)	150	MAPEG domain (PF01124) 8-131	R30, R31 and R104	18% ID 31% SIM	1.257 Å
Uncharacterized protein (CG33178, NP_788904.1)	165	MAPEG domain (PF01124) 29-159	R51, R53 and R139		

S17 Fig. Sequence and structural details of the modeled fly LTC4S candidate. A. Domain architecture of LTCS4 and CG33178 and known/predicted functional residues B. Pairwise alignment of CG33178 and 2PNO generated from structural superposition showing shared secondary structure elements and known/predicted functional residues (marked with red asterisks; the green asterisk denotes the analog for R31) C. Pairwise alignment of CG33178 and 2PNO generated from structural superposition with conserved residues highlighted using the physiochemical color scheme (CLUSTALX) D. Validation of the CG33178 model: ProQ2 quality score mapped to a 3D model of CG33178 (left); ProSA global quality score ranking (middle) and per-residue quality graph (right) E. LTCS4 (2PNO, cyan-blue) superimposed on the predicted structure of CG33178 (green-red) with potential matches for conserved functional residues highlighted F. Summary of features shared by LTCS4 and potential *D. melanogaster* ortholog CG33178.
