# Supplementary material for: A combined computational strategy of sequence and structural analysis predicts the existence of a functional eicosanoid pathway in Drosophila melanogaster
Source: PLoS One. 2019 Feb 12;14(2):e0211897. doi: 10.1371/journal.pone.0211897 (PMC6372189; doi:10.1371/journal.pone.0211897)
Supplement: S18 Fig — A. Domain architecture of ALOX5AP and CG33177 and known/predicted functional residues B. Pairwise alignment of CG33177 and 2Q7M generated from structural superposition showing shared secondary structure elements C. Pairwise alignment of CG33177 and 2Q7M generated from structural superposition with conserved residues highlighted using the physiochemical color scheme (CLUSTALX) D. Validation of the CG33177 model: ProQ2 quality score mapped to a 3D model of CG33177 (left); ProSA global quality score ranking (middle) and per-residue quality graph (right) E. ALOX5AP (2Q7M, cyan-blue) superimposed on the predicted structure of CG33177 (green-red) F. Summary of features shared by ALOX5AP and potential D. melanogaster ortholog CG33177. (PDF) [file pone.0211897.s018.pdf]

A.

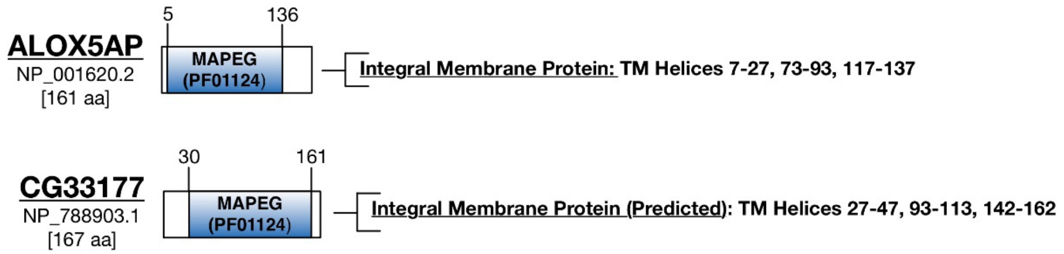

5
136
ALOX5AP
NP_001620.2
[161 aa]
MAPEG
(PF01124)
Integral Membrane Protein: TM Helices 7-27, 73-93, 117-137
30
161
CG33177
NP_788903.1
[167 aa]
MAPEG
(PF01124)
Integral Membrane Protein (Predicted): TM Helices 27-47, 93-113, 142-162

B.

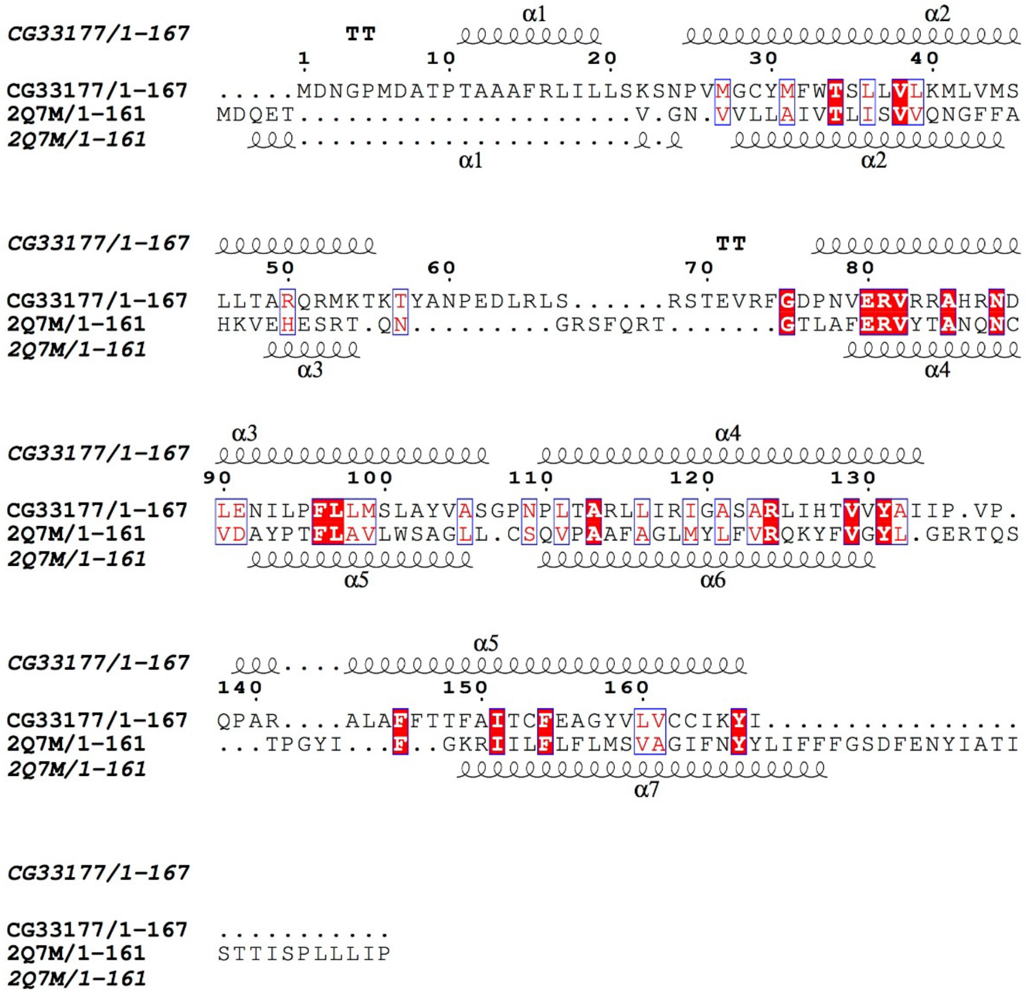

CG33177/1-167
TT
α1
α2
1 10 20 30 40
CG33177/1-167 .....MDNGPMDATPTAAAFRLILLSKSNPVMGCYMFWTSLLVLKMLVMS
2Q7M/1-161 MDQET.....................V.GN.VVLLAIVTLISVVQNGFFA
2Q7M/1-161
α1
α2
CG33177/1-167
TT
50 60 70 80
CG33177/1-167 LLTARQRMKTKTYANPEDLRLS......RSTEVRFGDPNVERVRRAHRND
2Q7M/1-161 HKVEHESRT.QN.........GRSFQRT.......GTLAFERVYTANQNC
2Q7M/1-161
α3
α4
α3
α4
CG33177/1-167
90 100 110 120 130
CG33177/1-167 LENILPFLLMSLAYVASGPNPLTARLLIRIGASARLIHTVVYAIIP.VP.
2Q7M/1-161 VDAYPTFLAVLWSAGLL.CSQVPAAFAGLMYLFVRQKYFVGYL.GERTQS
2Q7M/1-161
α5
α6
α5
CG33177/1-167
140 150 160
CG33177/1-167 QPAR....ALAFFTTFAITCFEAGYVLVCCIKYI................
2Q7M/1-161 ...TPGYI...F..GKRIILFLFLMSVAGIFNYYLIFFFGSDFENYIATI
2Q7M/1-161
α7
CG33177/1-167
CG33177/1-167 ....
2Q7M/1-161 STTISPLLLIP
2Q7M/1-161

C.

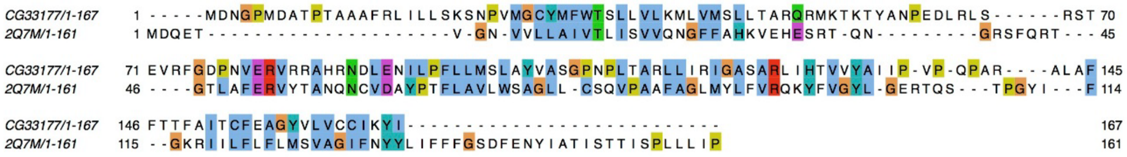

CG33177/1-167 1 -----MDNGPMDATPTAAAFRLILLSKSNPVMGCYMFWTSLLVLKMLVMSLLTARQRMKTKTYANPEDLRLS------RST 70
2Q7M/1-161 1 MDQET--------------------V-GN-VVLLAIVTLISVVQNGFFAHKVEHESRT-QN---------GRSFQRT--- 45
CG33177/1-167 71 EVRFGDPNVERVRRAHRNDLENILPFLLMSLAYVASGPNPLTARLLIRIGASARLIHTVVYAIIP-VP-QPAR----ALAF 145
2Q7M/1-161 46 ----GTLAFERVYTANQNCVDAYPTFLAVLWSAGLL-CSQVPAAFAGLMYLFVRQKYFVGYL-GERTQS---TPGYI---F 114
CG33177/1-167 146 FTTFAITCFEAGYVLVCCIKYI--------------------------- 167
2Q7M/1-161 115 --GKRIILFLFLMSVAGIFNYYLIFFFGSDFENYIATISTTISPLLLIP 161

D.

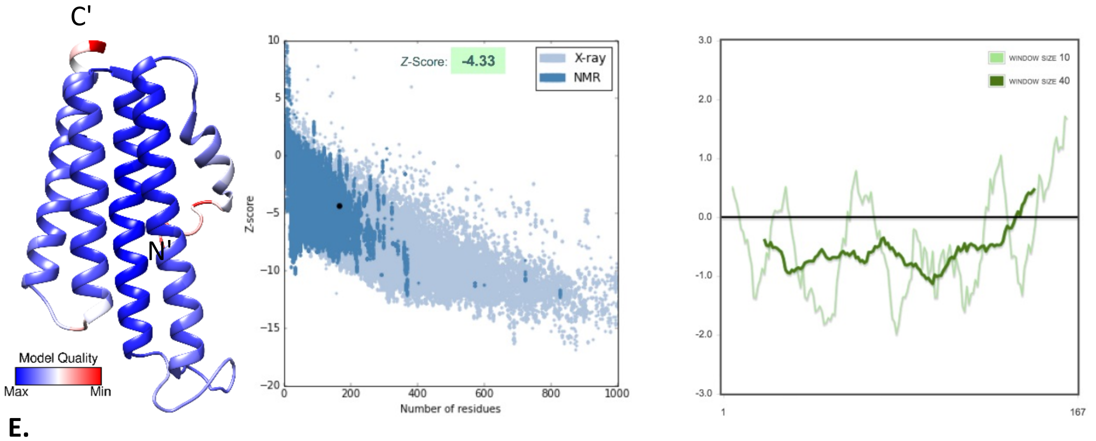

E.

ALOX5AP Structure	*D. melanogaster* Model	Superimposed
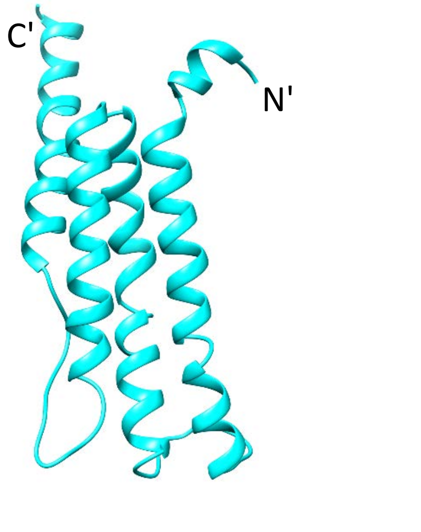	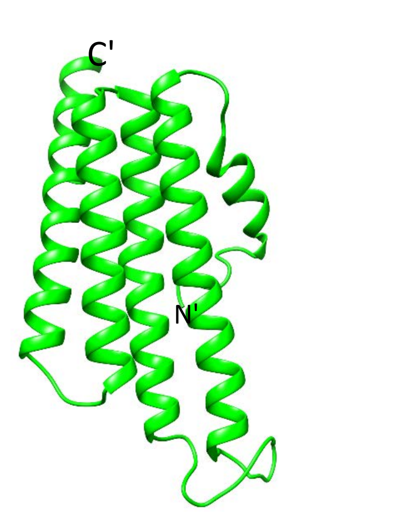	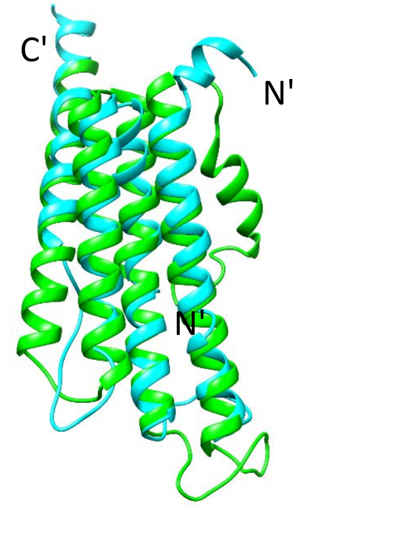

**F.**	Length (AA)	Domain Architecture (Pfam, range)	Functional Residues (aligned matches in *D. melanogaster*)	Sequence ID%	Structural Overlap (RMSD)
Arachidonate 5-lipoxygenase-activating protein (ALOX5AP, NP_001620.2, PDB: 2Q7M)	161	MAPEG domain (PF01124) 5-136	N/A	13% ID<br>28% SIM	1.024 Å
Uncharacterized protein CG33177, NP_788903.1)	167	MAPEG domain (PF01124) 30-161	N/A		

S18 Fig. Sequence and structural details of the modeled fly ALOX5AP candidate. A. Domain architecture of ALOX5AP and CG33177 and known/predicted functional residues B. Pairwise alignment of CG33177 and 2Q7M generated from structural superposition showing shared secondary structure elements C. Pairwise alignment of CG33177 and 2Q7M generated from structural superposition with conserved residues highlighted using the physiochemical color scheme (CLUSTALX) D. Validation of the CG33177 model: ProQ2 quality score mapped to a 3D model of CG33177 (left); ProSA global quality score ranking (middle) and per-residue quality graph (right) E. ALOX5AP (2Q7M, cyan-blue) superimposed on the predicted structure of CG33177 (green-red) F. Summary of features shared by ALOX5AP and potential *D. melanogaster* ortholog CG33177.
